# On the relevance of query definition in the performance of 3D ligand-based virtual screening

**DOI:** 10.1007/s10822-024-00561-5

**Published:** 2024-04-04

**Authors:** Javier Vázquez, Ricardo García, Paula Llinares, F. Javier Luque, Enric Herrero

**Affiliations:** 1 Pharmacelera, Parc Científic de Barcelona (PCB), C/ Baldiri Reixac 4-8, Barcelona, 08028 Spain; 2 Departament de Nutrició, Ciències de l’Alimentació i Gastronomia, Facultat de Farmàcia i Ciències de l’Alimentació, Institut de Química Teòrica I Computacional (IQTC-UB), Institut de Biomedicina (IBUB), University of Barcelona, Av. Prat de la Riba 171 , Santa Coloma de Gramenet, -08921 Spain

**Keywords:** Virtual screening, 3D similarity, Field-based alignment, Query conformation

## Abstract

Ligand-based virtual screening (LBVS) methods are widely used to explore the vast chemical space in the search of novel compounds resorting to a variety of properties encoded in 1D, 2D or 3D descriptors. The success of 3D-LBVS is affected by the overlay of molecular pairs, thus making selection of the template compound, search of accessible conformational space and choice of the query conformation to be potential factors that modulate the successful retrieval of actives. This study examines the impact of adopting different choices for the query conformation of the template, paying also attention to the influence exerted by the structural similarity between templates and actives. The analysis is performed using PharmScreen, a 3D LBVS tool that relies on similarity measurements of the hydrophobic/philic pattern of molecules, and Phase Shape, which is based on the alignment of atom triplets followed by refinement of the volume overlap. The study is performed for the original DUD-E^+^ database and a Morgan Fingerprint filtered version (denoted DUD-E^+^-Diverse; available in https://github.com/Pharmacelera/Query-models-to-3DLBVS), which was prepared to minimize the 2D resemblance between template and actives. Although in most cases the query conformation exhibits a mild influence on the overall performance, a critical analysis is made to disclose factors, such as the content of structural features between template and actives and the induction of conformational strain in the template, that underlie the drastic impact of the query definition in the recovery of actives for certain targets. The findings of this research also provide valuable guidance for assisting the selection of the query definition in 3D LBVS campaigns.

**Graphical Abstract:**

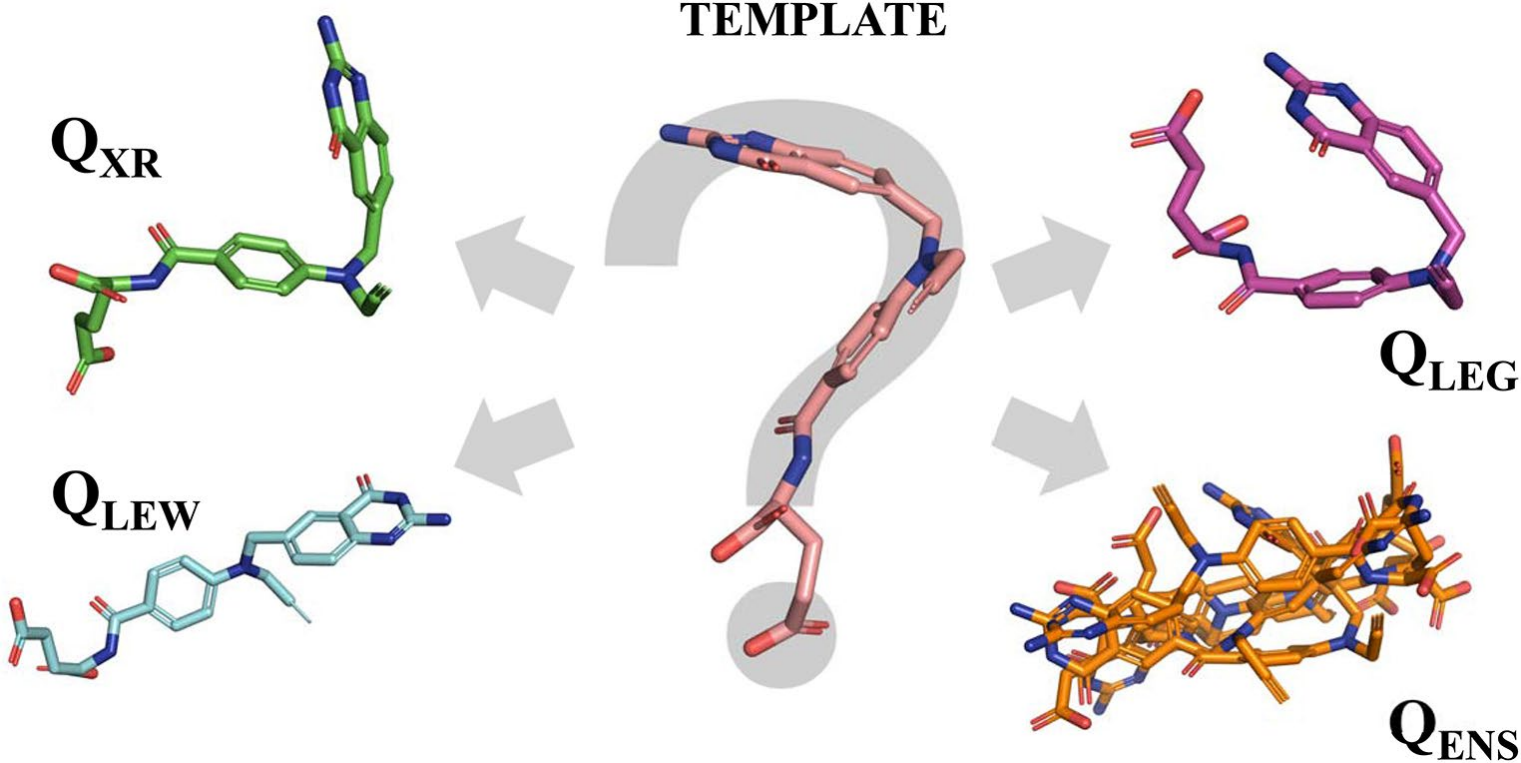

**Supplementary Information:**

The online version contains supplementary material available at 10.1007/s10822-024-00561-5.

## Introduction

Ligand-based virtual screening (LBVS) is widely used in computer-aided drug-discovery for multiple applications, such as hit identification, scaffold hopping, target fishing and drug repurposing, and is becoming increasingly popular due to the expansion of the chemical space available for screening studies [1–3]. The concept of LBVS encompasses a variety of computational methods that exploit the chemical information of known active molecules in the absence of the 3D structural knowledge of the target [4, 5]. However, they also are valuable to assist and complement the analysis of active compounds when used in conjunction with structure-based virtual screening methods [6–10].

LBVS techniques are generally categorized according to the descriptors used to collect the relevant features of the molecular representations selected for the comparison between molecules [11, 12]. 1D- and 2D-based methods rely on atomic properties and connectivity information, commonly computing similarities through the comparison of fingerprints, enabling a fast screening of the chemical space [13]. On the other hand, 3D-based techniques exploit conformation-dependent properties, aiming to gain insight into ligand-receptor interactions from the 3D-spatial distribution of properties such as shape, electrostatic distribution, and lipophilicity. 3D-LBVS relies on the bioactive conformation of the template compound, which reflects the pose of the ligand upon binding to the biological target complex, as observed in the X-ray structure of the ligand-target complex. When the bioactive conformation of the template is not available from the crystallographic structure of the target, it can be inferred from docking calculations, often guided with information about pharmacophore hot spots [14, 15].

In the absence of detailed structural information of the target LBVS faces a more challenging scenario. In this context, a low-energy conformation or a subset of accessible conformers of the template can be used. This option assumes that the bioactive conformation must be close to one of the conformational minima of the unbound ligand in aqueous solution, as this would imply a low conformational penalty for the transition from the unbound ligand in solution to the ligand-target complex [16, 17]. On the other hand, the use of ensembles of active analogs of the template compound can be adopted to improve the performance in the screening of chemical libraries [18, 19].

This context justifies the interest spent in examining the impact of how the selection of the template conformation can influence the performance of the 3D-LBVS, since factors such as the conformational sampling of the template or the choice of a specific conformation as a query or the use of multiple queries might influence the search of the chemical space. It is generally assumed that the choice of the query conformation has a modest impact on the enrichment rates of the LBVS [20–23]. This finding could be affected by the structural resemblance between the bioactive (i.e., crystallographic) conformation and the low-energy conformer obtained from the conformational sampling of the template [24]. Nevertheless, it is known that the bioactive conformation does not always correspond to the global energy minimum of the free (unbound) ligand nor even to a local minimum. Furthermore, the lower performance obtained for the crystallographic structure relative to other low-energy conformations might suggest that the molecular descriptors may have limited suitability to account for a fine 3D comparison between template and actives. Finally, the 2D bias between the template compound and actives included in the benchmarking datasets may also influence the outcome of the LBVS. Thus, a high percentage of hits with large 2D resemblance to the template compound in public benchmarks may implicitly convert the VS in a mapping process of common 2D patterns [25, 26], where 3D conformational data becomes less relevant.

The effect of these factors may remain unnoticed when attention is paid to the analysis of the global performance obtained for large ensembles of targets. However, they may have an impact in the outcome of LBVS studies for individual templates. Accordingly, choice of the appropriate query structure could lead to a sensible gain in the performance of the screening. In this context, the availability of guidelines to anticipate the suitability of distinct query definitions could be useful. To this end, this study examined the effect of the 2D bias in benchmarking datasets on the performance of 3D-LBVS methods when used in conjunction with distinct query conformations. Specifically, attention is paid to the impact of the 3D information of the query conformation on the performance of the screening through the analysis of the results obtained from distinct protocols designed to define the 3D structure of the query, including single and multiple conformational states of the template. The results are examined for selected representative cases. Finally, the potential factors that may be considered to guide the selection of the query conformation are discussed.

## Methods

**Fig. 1:**
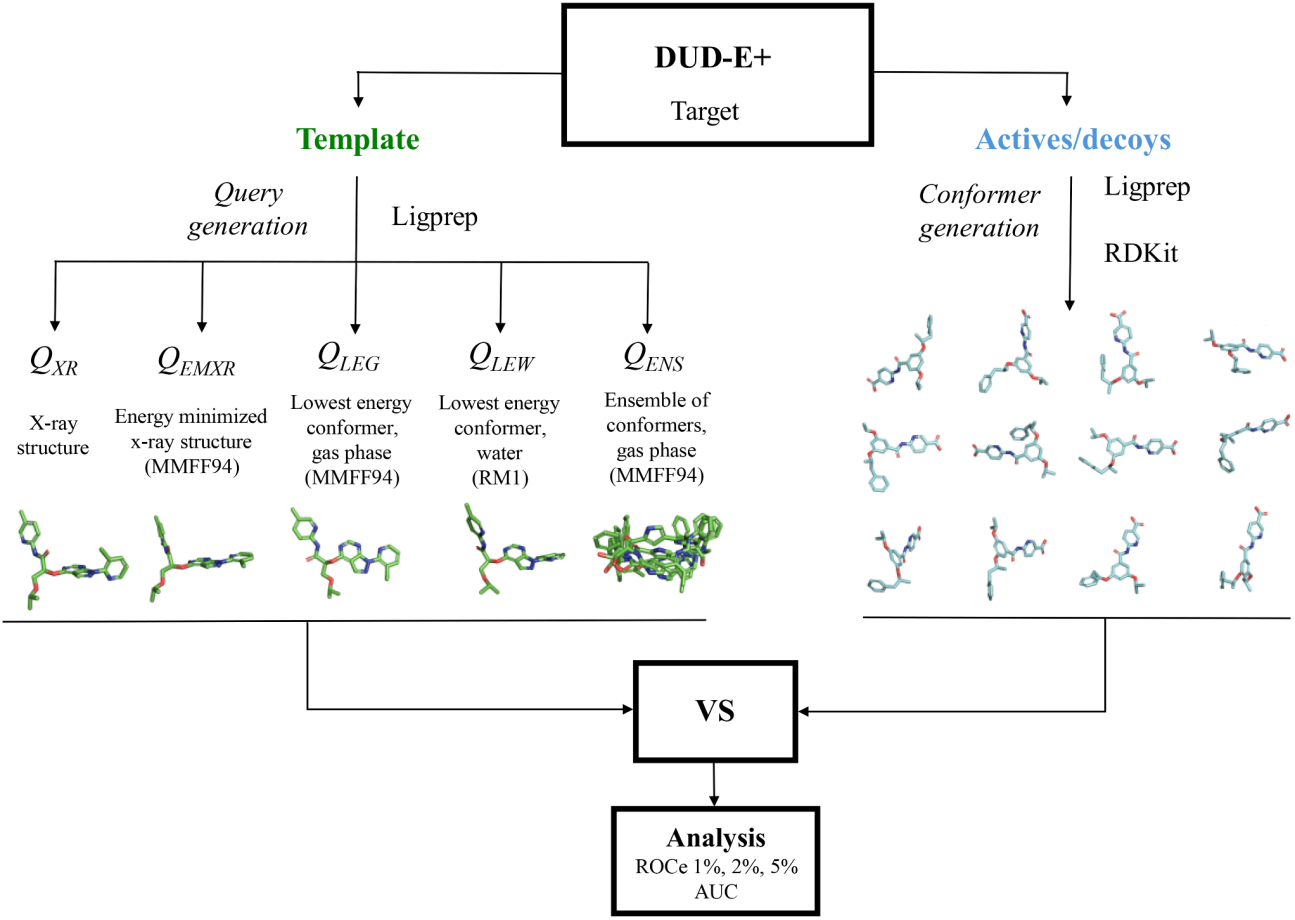
Schematic representation of the flowchart followed to examine the influence of the query conformation on the results of the VS

The general procedure adopted to calibrate the effect of the query conformation on the outcome of the LBVS is outlined in Fig. 1. A detailed description of the different steps is provided in the following sections.

### Test sets

DUD-E^+^ and DUD-E^+^-Diverse datasets have been used to examine how the 2D resemblance in benchmarking datasets may affect the outcome of 3D-LBVS.

DUD-E^+^ is a publicly available database (https://www.jainlab.org/downloads/) that contains 92 protein targets. Each target is associated to known actives mainly extracted from ChEMBL (https://www.ebi.ac.uk/chembl/), and mimetic decoys generated to model physical properties of the actives. For each molecule, the stereochemistry information of actives and decoys is provided (see Supporting Information Table S1 for a description of the dataset).

DUD-E^+^-Diverse is a subset of DUD-E^+^ that has been curated to reduce the influence of structural analogs of the template. To this end, a Morgan fingerprint (radius = 2; 2048 bits) [27] was applied to evaluate the 2D structural resemblance of actives and decoys to the template. Then, the selection of the targets retained in DUD-E^+^-Diverse was made according to two criteria:

i) for each target, the Tanimoto index of actives should be, on average, close to 0.1 and the distribution of values determined for actives and decoys should be comparable, which led to the removal of 82.5% of the actives, and.
ii) the relative population between actives and decoys, which amounts on average to 50 for the targets in the DUD-E^+^ dataset, should be equal or lower than 200.

Overall, 15 targets that fulfilled these criteria were retained for this study (see Supporting Information Table S2).

### Preparation of actives and decoys

In the DUD-E^+^ database, each active and decoy is represented by a single protomer defined by a unique SMILES code, specifying a distinct stereoisomer and tautomer. Accordingly, Ligprep 3.4 [28] was used to generate the 3D structures of the compounds. The protonation state of each compound was assigned at physiological pH, and each SMILES reported a single molecule (neither multiple tautomers nor alternative stereoisomers were generated). Conformers were generated using RDKit’s ETKDG method [29]. On average, 23 conformations were generated for each ligand (the number of conformers for each set is shown in Supporting Information Fig. S1).

### Preparation of conformational queries of the template ligand

In DUD-E^+^ each target is associated to five co-crystallized ligands. For our purposes in this study, the ligand with the highest number of rotatable bonds among the five cognates associated to a given target was chosen as template. This choice aims to maximize the complexity of the accessible conformational space, thus favoring the identification of structurally diverse low-energy conformers. In contrast, choice of a template with limited conformational flexibility would have minimized the differences between the distinct query definitions. A detailed list of the selected templates is provided in Supporting Information Tables S1 and S2. For each template, five query conformations were generated:

i) the X-ray structure (*Q*_*XR*_),
ii) the energy-minimized crystallographic structure (*Q*_*EMXR*_),
iii) the lowest energy conformer sampled for the free compound in the gas phase (*Q*_*LEG*_),
iv) the lowest energy conformer of the free compound in water (*Q*_*LEW*_), and.
v) an ensemble of accessible conformers in the gas phase (*Q*_*ENS*_).

The geometry of *Q*_*EMXR*_ was obtained upon minimization of the X-ray crystallographic ligand using the MMFF94 force-field [30], which is widely used in conformer generative methods [31, 32]. With regard to *Q*_*LEG*_, *Q*_*LEW*_ and *Q*_*ENS*_, the RDKit’s ETKDG method was used to generate multiple conformers from the SMILES of the template compound. For each ligand, the rotatable bonds were retrieved using a SMARTS pattern, and the dihedral angle (θ) of each rotatable bond (*i*) in the set of generated conformers was encoded into a trigonometric tuple {cos(θ_i_), sin(θ_i_)}. Then, Principal Component Analysis was used to reduce the dimensionality of the space defined by the dihedral vectors that characterize all the conformers, assuring that at least 75% of the variance is retained. Clustering of the PCA-projected dihedral space was performed using the *k*-means algorithm. For our purposes, the number of clusters was set to 5 according to the preliminary analysis performed for a small subset of chemically diverse ligands. Finally, the centroid structure of each cluster was energy-minimized with the MMFF94 force-field. The lowest energy conformation of the template in the gas phase was used for *Q*_*LEG*_.

Alternatively, the geometry of the centroids was optimized using the semiempirical RM1 Hamiltonian [33], and the relative stability in aqueous solution was determined by adding the solvation free energy calculated using the RM1-parametrized version of the IEFPCM/MST continuum solvation model [34]. The lowest energy conformation of the template was used for *Q*_*LEW*_.

Finally, the subset of energy-minimized conformational centroids obtained from the clustering of the MMFF94 conformational sampling was used to define *Q*_*ENS*_. Let us note that the clustering approach leads to positional root-mean square deviation (RMSD) distributions that span up to 5 Å (see Fig. S2 in Supporting Information), although the magnitude is largely affected by the number of rotatable bonds in the template (see for instance **prgr** and **urok**). The python script to perform the clustering in the ensemble query (*Q*_*ENS*_) is available in the public GitHub repository at https://github.com/Pharmacelera/Query-models-to-3DLBVS.

### Virtual screening tool

To examine the sensitivity of the analysis to the molecular features used in the similarity between query and hits, the screening was performed using two different LBVS tools, PharmScreen [35] and Phase Shape [21]. Pharmscreen relies on the 3D atomic hydrophobicity maps determined from Hyphar parameters [36], which are derived from self-consistent reaction field quantum mechanical computations of the ligand in water and *n*-octanol with the IEFPCM/MST model. In this study Hyphar parameters were derived using the MST version parametrized at the semiempirical RM1 level [30]. The similarity between molecules is determined by maximizing the 3D atomic hydrophobic contributions of the compounds, supplemented with information about the distribution of hydrogen-bond (HB) donor/acceptor groups along the molecular skeleton. In contrast, Phase is a pharmacophore/volume overlap modeling solution where each ligand is represented by a set of points that account for specific chemical features in 3D space. Phase Shape describes the ligand as a set of van der Waals spheres and the overlap between molecules A and B is computed as the sum of pairwise atomic overlaps. The superposition of molecules is based on the alignment of pairs of triplets of atoms with similar geometries.

### Data fusion

To combine the results obtained when multiple queries are used, three fusion algorithms were considered: sum rank, MAX score, and parallel selection [37, 38].

i) Sum rank. This method adds together the ranks using the arithmetic sum of the position from the different VS methods’ rank lists and reorders accordingly.
ii) MAX score. It collects the best similarity value that is achieved for each compound in the complete set of *n* similarity rankings to define its position in the final ranking.
iii) Parallel selection. The rank position of each compound is chosen from the best position of the n similarity rankings. Ties are broken using arithmetic sum of positions.

### Performance evaluation

Several metrics are commonly used to evaluate the quality of ranking methods in VS [39–41]. In this work, we have used the Receiver Operator Characteristic enrichment factor (ROCe) and the Area Under the Curve (AUC).

(i) ROCe. This metric (Eq. 1) is defined as the ratio of the true positive rate to the false positive rate, for a given proportion of the known decoys having been observed. ROCe values at 1.0, 2.0 and 5.0% are considered in this study.

1 $$ROCe \left(x\%\right)=\frac{\frac{{N}_{actives}^{x\%}}{{N}_{total}}}{\frac{{N}_{decoys}^{x\%}}{{N}_{total}}}$$

where ${N}_{actives}^{x\%}$ and ${N}_{decoys}^{x\%}$ stand for the number of actives and decoys retrieved at a specific x% rate, and *N*_*total*_ is the total number of compounds in the set.

(ii) AUC is a global measure of the performance of the VS methods across the entire ranked database.

## Results and discussion

### Chemical diversity in DUD-E^+^ and DUD-E^+^-diverse

Prior to the analysis of the impact of the query definition on the outcome of the VS, the degree of structural resemblance of the chemical space defined by actives and decoys in the DUD-E^+^ database was examined. To this end, for each target the similarity between actives and decoys relative to the template compound was estimated by means of the Tanimoto index using a 2D Morgan fingerprint (radius = 2; 2048 bits; see above).

**Fig. 2:**
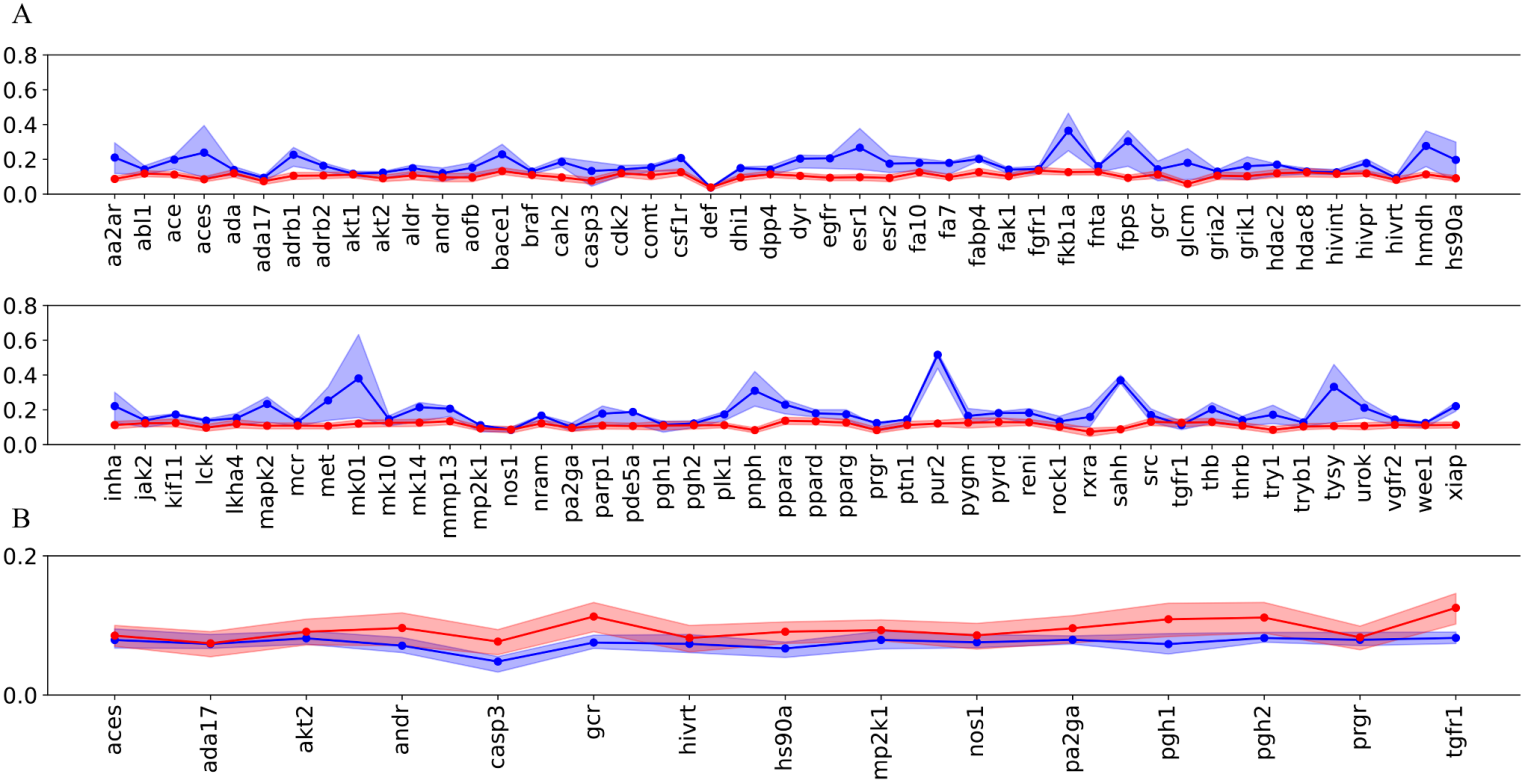
2D Structural similarity determined by using the Tanimoto index (Morgan fingerprint) for actives (blue) and decoys (red) relative to the template compound for the targets included in (**A**) DUD-E^+^ and (**B**) DUD-E^+^-Diverse datasets. Dots stand for the average value of actives and decoys in each dataset. The blue/red areas denote the similarity indexes corresponding to the first and forth quartile for each target

Figure 2A shows that the actives included in DUD-E^+^ generally exhibit a wider range of similarity to the template compound, which is in contrast with the rather uniform deviation obtained for the decoys. Compared to the template, the Tanimoto similarity of decoys is generally comprised between 0.03 and 0.14, and the global similarity determined for the whole set of 92 targets amounts, on average, to 0.11. The similarity index determined for the actives exhibit larger differences, and in 23 cases is ≥ 0.2. In light of these results, the DUD-E^+^-Diverse subset was generated as a curated dataset aimed to reduce the impact of structural analogs of the template compounds. To this end, only actives with low Tanimoto similarity ( ≤ 0.1) were retained to minimize the potential influence of the 2D bias on the results of the VS. As noted in Fig. 2B, both actives and decoys exhibit close similarity profiles to the template for the 15 datasets retained in in the DUD-E^+^-Diverse.

### Structural resemblance between distinct query definitions

As noted above, distinct protocols were considered to generate the query conformation of the template compound, including the preservation of the X-ray structure (*Q*_*XR*_) or its energy-minimized structure (*Q*_*EMXR*_), or alternatively the conformers corresponding to the lowest energy structures in the gas phase (*Q*_*LEG*_) and in aqueous solution (*Q*_*LEW*_), and finally an ensemble of conformations (*Q*_*ENS*_). The structural similarity between the query conformations relative to the crystallographic geometry was estimated from the RMSD determined for the heavy atoms of the template compound.

**Fig. 3:**
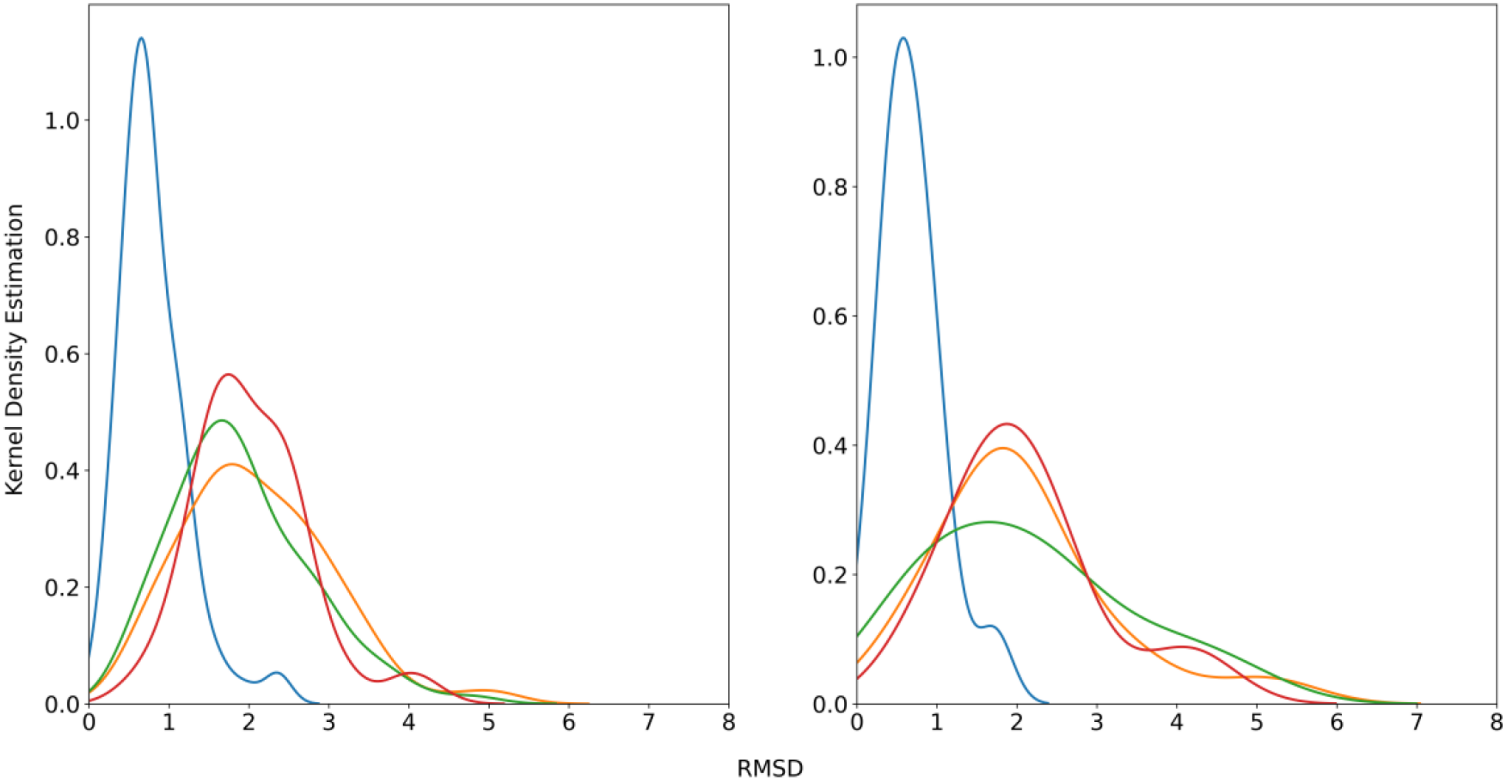
Symmetry-corrected heavy atom root-mean square deviation (RMSD; Å) profiles determined relative to the X-ray structure (*Q*_*XR*_) for the distinct query definitions (*Q*_*EMXR*_: blue; *Q*_*LEG*_: orange; *Q*_*LEW*_: green; *Q*_*ENS*_: red) of the template compounds included in the (left) DUD-E^+^ dataset and (right) the subset of targets of the DUD-E^+^-Diverse dataset

Compared to the X-ray conformation, the energy minimized structure leads to a narrow RMSD distribution with a peak centered at 0.7 Å (Fig. 3A). A wider distribution is obtained for the set of lowest-energy conformations (*Q*_*LEG*_ and *Q*_*LEW*_) and for the multiple query (*Q*_*ENS*_), which exhibit a similar profile characterized by a broader RMSD peak centered at 1.9–2.2 Å. Moreover, the tail of the RMSD distribution is extended up to 6 Å. These trends are preserved in the subset of 15 datasets included in the DUD-E^+^-Diverse (Fig. 3B). Overall, although sampling of the conformational space increases the dissimilarity between the conformations of the template, there is generally a large resemblance between the distinct definitions of the query conformer, and only 25–35% of the *Q*_*LEG*_, *Q*_*LEW*_ and *Q*_*ENS*_ conformers are characterized by RMSD values > 3 Å.

### Performance of a single query on the virtual screening with pharmscreen

The VS results obtained for the DUD-E^+^ dataset using the distinct query conformations are examined in this section. The discussion is generally focused on the analysis of the global trends, paying special attention to the ROCe (1%, 2% and 5%) values as long as no relevant differences were observed in the AUC curves (see Supporting Information Table S3 for information about the ROCe and AUC values for the entire set of targets, and results for individual sets in Tables S4 − S7).

**Fig. 4:**
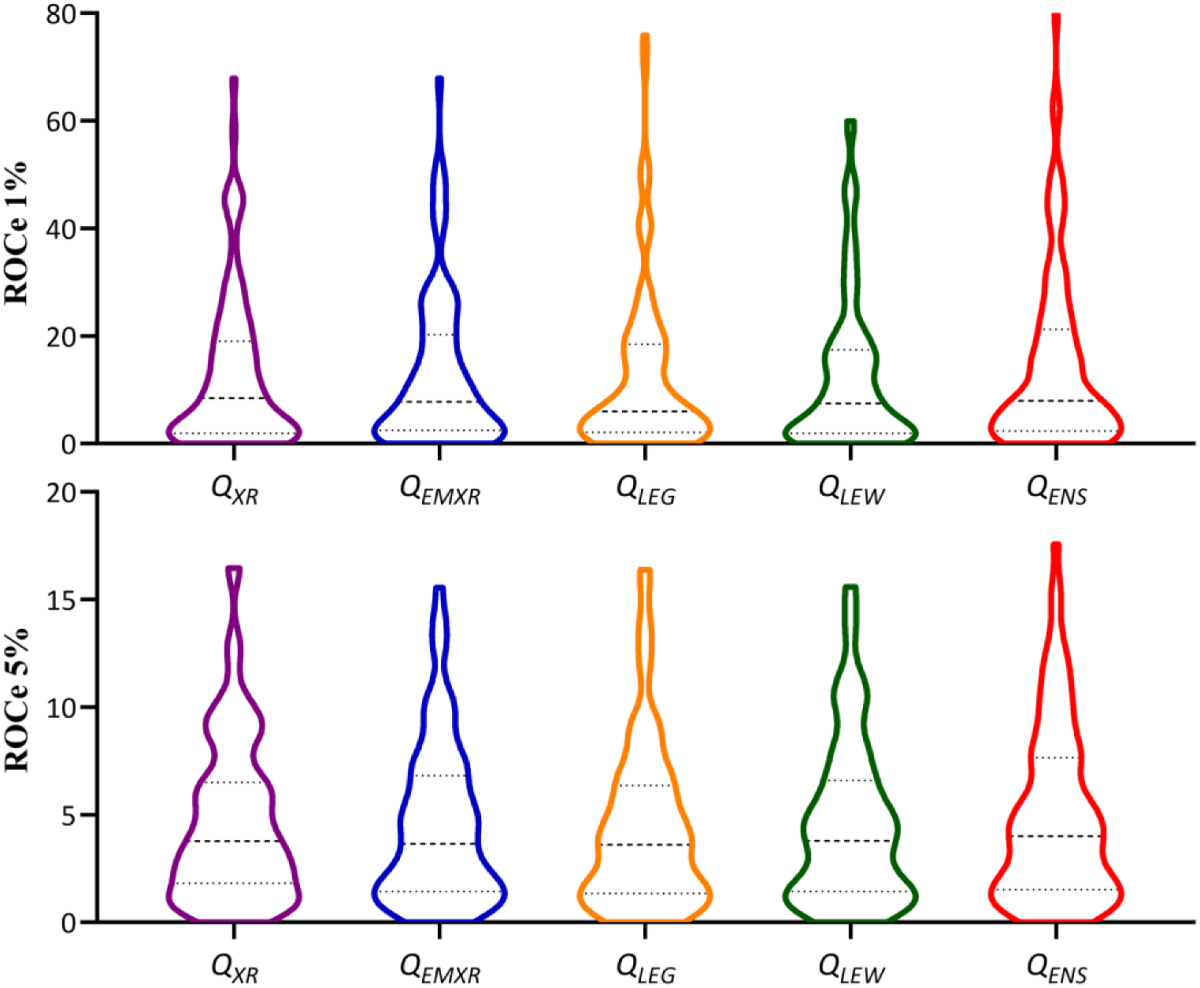
Distribution of ROCe values (top: 1%; bottom: 5%) determined for the set of targets included in DUD-E^+^ dataset using the distinct query definitions for the template

Figure 4 displays the distribution of ROCe (1% and 5%) values obtained for the whole set of targets using measurements of the PharmScreen similarity for the distinct query definitions. The distribution profiles are highly similar. Indeed, paired t-tests confirmed that no statistically significant differences between the ROCe distribution obtained for the distinct queries relative to the profile obtained for the *Q*_*XR*_ conformer were found. The similar performance obtained between *Q*_*XR*_ and *Q*_*EMXR*_ is not unexpected due to the close structural resemblance of the corresponding conformations, as 90% of the energy-minimized compounds have a RMSD deviation < 1 Å from the X-ray structure (Fig. 3). A similar performance is obtained for *Q*_*LEG*_, *Q*_*LEW*_ and *Q*_*ENS*_ despite the shift in the position of the peak and the larger tails found in the RMSD profiles relative to the X-ray conformation (Fig. 3).

**Fig. 5:**
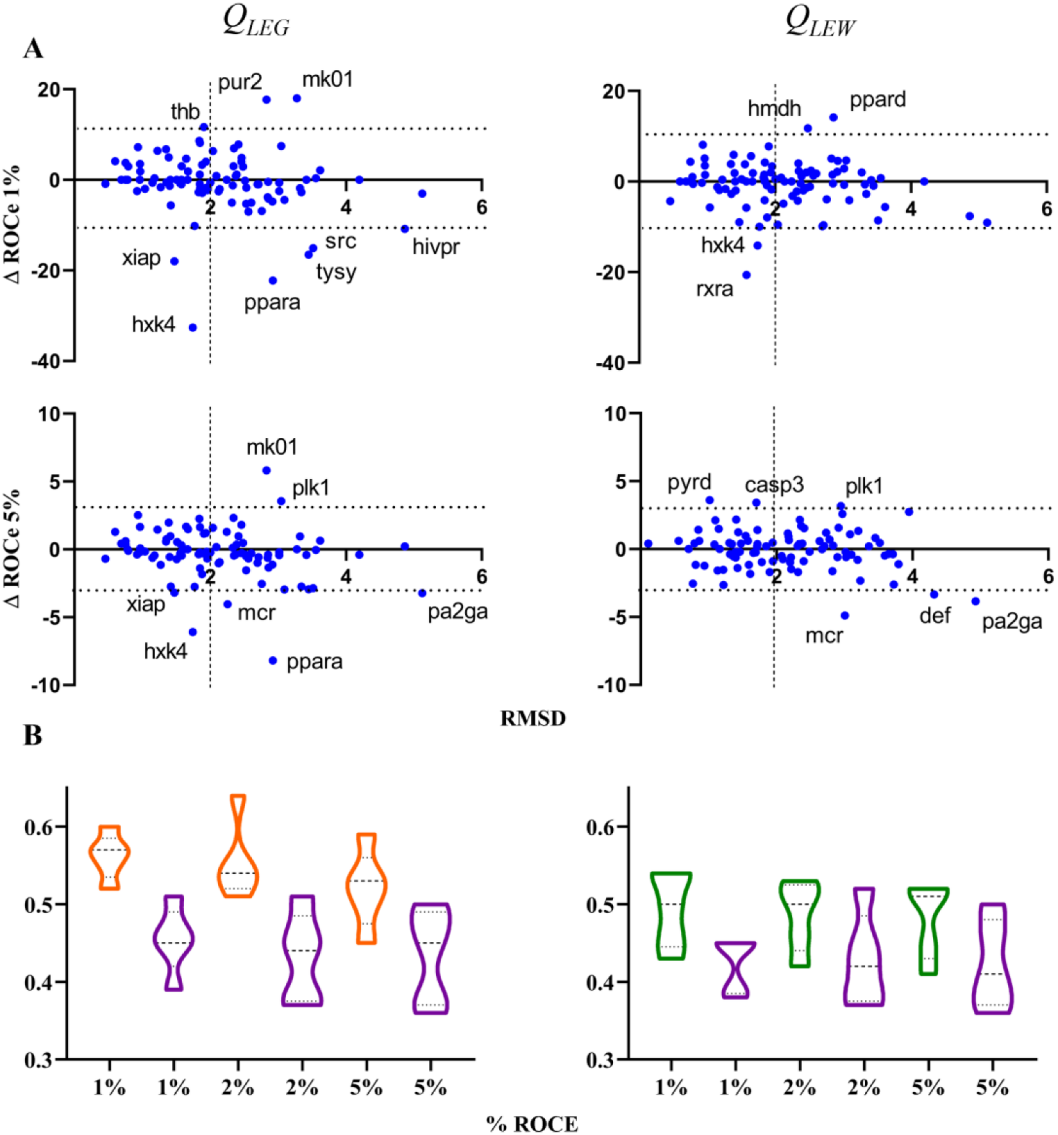
**A**) Representation of the difference in ROCe (ΔROCe) 1 and 5% versus the RMSD between query conformation obtained for (left) *Q*_*LEG*_ and (right) *Q*_*LEW*_ queries relative to the X-ray crystallographic one (*Q*_*XR*_) for the targets included in the DUD-E^+^ dataset. **B**) Distribution of the actives uniquely recovered at 1, 2 and 5% according to the structural overlap (0: minimum overlap; 1: maximum overlap) exhibited against the *Q*_*XR*_ (magenta), *Q*_*LEG*_ (orange) and *Q*_*LEW*_ (green) queries. The structural overlap was estimated from the Tanimoto coefficient obtained from the comparison of the projection of the van der Waals radii of atoms onto a grid (spacing of 0.5 Å) of the hydrophobicity-guided alignment of query and active. Dashed lines denote the average value, and upper/lower dotted lines stand for the 25%/75% of the cases

In agreement with previous studies [20–23], Fig. 4 suggests that the choice of the.

query conformation has *globally* a minor effect on the outcome of the VS. However, this does not imply that sensible differences might be found for individual targets. In fact, this can be noticed in Fig. 5A, which shows the representation of the difference in the ROCe values (ΔROCe) obtained for the *Q*_*LEG*_ and *Q*_*LEW*_ queries relative to the *Q*_*XR*_ in front of the RMSD determined between these conformations. Similar enrichments (|ΔROCe| < 1) are obtained for most of the targets. Nevertheless, there is a subset of targets where larger ΔROCe values are found, often revealing a better retrieval of actives when the *Q*_*LEG*_/*Q*_*LEW*_ conformers are used in the LBVS (positive values of ΔROCe), as noted for **mk01**, **hxha** and **pur2**, or alternatively when the *Q*_*XR*_ query is adopted as query conformations (negative values of ΔROCe), such as **ppara**, **mcr** and **xiap** (see Table 1). Noteworthy, this behavior is not strictly associated to the RMSD between the query conformations, since |ΔROCe| values larger/lower than 10 (ROCe 1%) and 3 (ROCe 5%) are observed for cases characterized with highly similar conformations (RMSD values between queries ≤ 2 Å). Furthermore, inspection of Fig. 5B reveals that the actives recovered exclusively in the VS performed for the *Q*_*LEG*_/*Q*_*LEW*_ queries (i.e., not found in the VS carried out against the *Q*_*XR*_ query) consistently exhibit a larger structural overlap with *Q*_*LEG*_/*Q*_*LEW*_ compared to the overlap obtained for the actives retrieved uniquely when the *Q*_*XR*_ query is used in the LBVS.

Overall, this analysis points out that for certain targets the recovery of actives can be largely affected by the choice of the query definition. This trait cannot be attributed to geometrical differences between query definitions, as they are found along the whole range of RMSD values. Rather, it can be ascribed to the chemical resemblance between actives and template, as long as the presence of a common structural motif would favor the generation of similar conformations upon energy minimization either in the gas phase or in solution. In turn, this would justify why the choice of *Q*_*LEG*_/*Q*_*LEW*_ queries favors the identification of actives with a large hydrophobic resemblance, since the chemical skeleton shared by template and actives would tend to maximize the overlap of the 3D hydrophobic distribution. In contrast, choice of the X-ray conformation may be better suited when there is low chemical resemblance between template and actives, as the crystallographic structure may be affected by factors that cannot be inherently associated to the conformational sampling, such as steric constraints imposed upon binding to the target, which would explain the lower degree of hydrophobic similarity shown in Fig. 5B.

**Table 1:** ROCe 1 and 5% values obtained for selected targets characterized by large |ΔROCe| values when *Q*_*XR*_, *Q*_*LEG*_ and *Q*_*LEW*_ queries are used in the screening of the DUD-E^+^ dataset

Target	Reference ID Target-Ligand	Q_XR_	Q_LEG_	ΔROCe	Q_LEW_	ΔROCe
ROCe 1%						
mk01	3I5Z-Z48	22.8	40.5	+ 17.7		
pur2	4EW3-DXZ	58.0	76.0	+ 18.0		
thb	1Q4X-G24	29.1	40.8	+ 11.7		
hivpr	1EBZ-BEC	28.7	17.9	-10.8		
hxk4	4IXC-1JD	35.9	3.3	-32.6		
ppara	1I7G-AZ2	23.3	1.1	-22.2		
src	2OIQ-STI	27.3	12.2	-15.1		
tysy	1TRG-CB3	41.3	24.8	-16.5		
xiap	4HY0-1AQ	25.0	7.0	-18.0		
hmdh	2R4F-RIE1	46.5			58.2	+ 11.7
ppard	5U3Z-7UA	15.8			30.0	+ 14.2
hxk4	4IXC-1JD	35.9			21.7	-16.2
rxra	4RMD-3SW	67.9			47.3	-20.6
ROCe 5%						
mk01	3I5Z-Z48	6.3	12.2	+ 5.9		
plk1	4A4L-939	1.1	4.7	+ 3.6		
hxh4	4IXC-1JD	10.2	4.1	-6.1		
mcr	5L7G-6QE	5.1	1.1	-4.0		
pa2ga	1AYP-INB	7.3	4.0	-3.3		
ppara	1I7G-AZ2	8.9	0.7	-8.2		
xiap	4HY0-1AQ	9.4	6.2	-3.2		
casp3	4QU9-ACE	2.8			6.2	+ 3.4
plk1	4A4L-939	1.1			4.3	+ 3.2
pyrd	3KVJ-1 × 5	1.8			5.4	+ 3.6
def	1ICJ-2PE	3.5			0.2	-3.3
mcr	5L7G-6QE	5.1			0.9	-4.2
pa2ga	1AYP-INB	7.3			4.2	-3.1

**Fig. 6:**
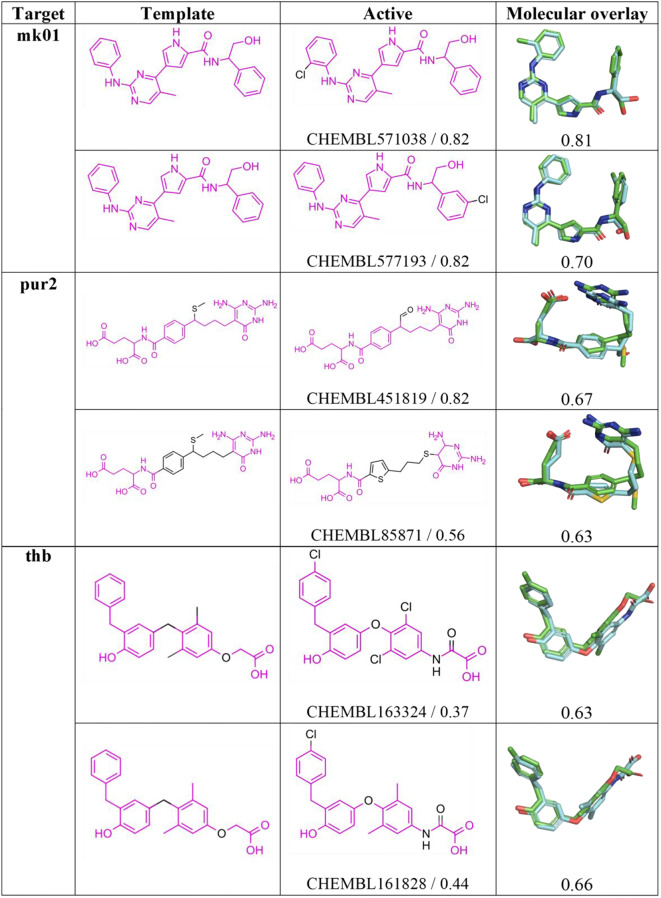
2D Structure of query and top-ranked actives for selected targets where choice of the *Q*_*LEG*_ query leads to a better performance in the VS relative to the X-ray structure. For each active compound, the structural resemblance (Tanimoto index using Morgan Fingerprint, radius 2) with the template and the structural overlap between the overlaid *Q*_*LEG*_ query and active are indicated

To test the preceding hypothesis, Fig. 6 compares the 2D structures of template and top-ranked actives retrieved using the *Q*_*LEG*_ query for **mk01**, **pur2** and **thb** and the 3D overlaid structures of both query and active. Noteworthy, the *N*-(2-hydroxy-1-phenylethyl)-1*H*-pyrrole-2-carboxamide moiety present in the template of **mk01** is shared with the top-ranked actives (Tanimoto index of 0.82), reflecting the large chemical similarity found for the actives in this target (see Fig. 2A). Therefore, the resemblance of the chemical scaffold should favor the generation of conformations for the actives exhibiting a pronounced overlap with the template *Q*_*LEG*_ query, as noted in the 3D structural overlap between the volumes of query and active (≥ 0.70). A similar behavior is expected to occur in targets **pur2** and **thb** due to the preservation of a common chemical scaffold in the 2D structures of template and top-ranked actives, which facilitates a large 3D overlap between the volumes of the aligned compounds (close to 0.65; see Fig. 6).

**Fig. 7:**
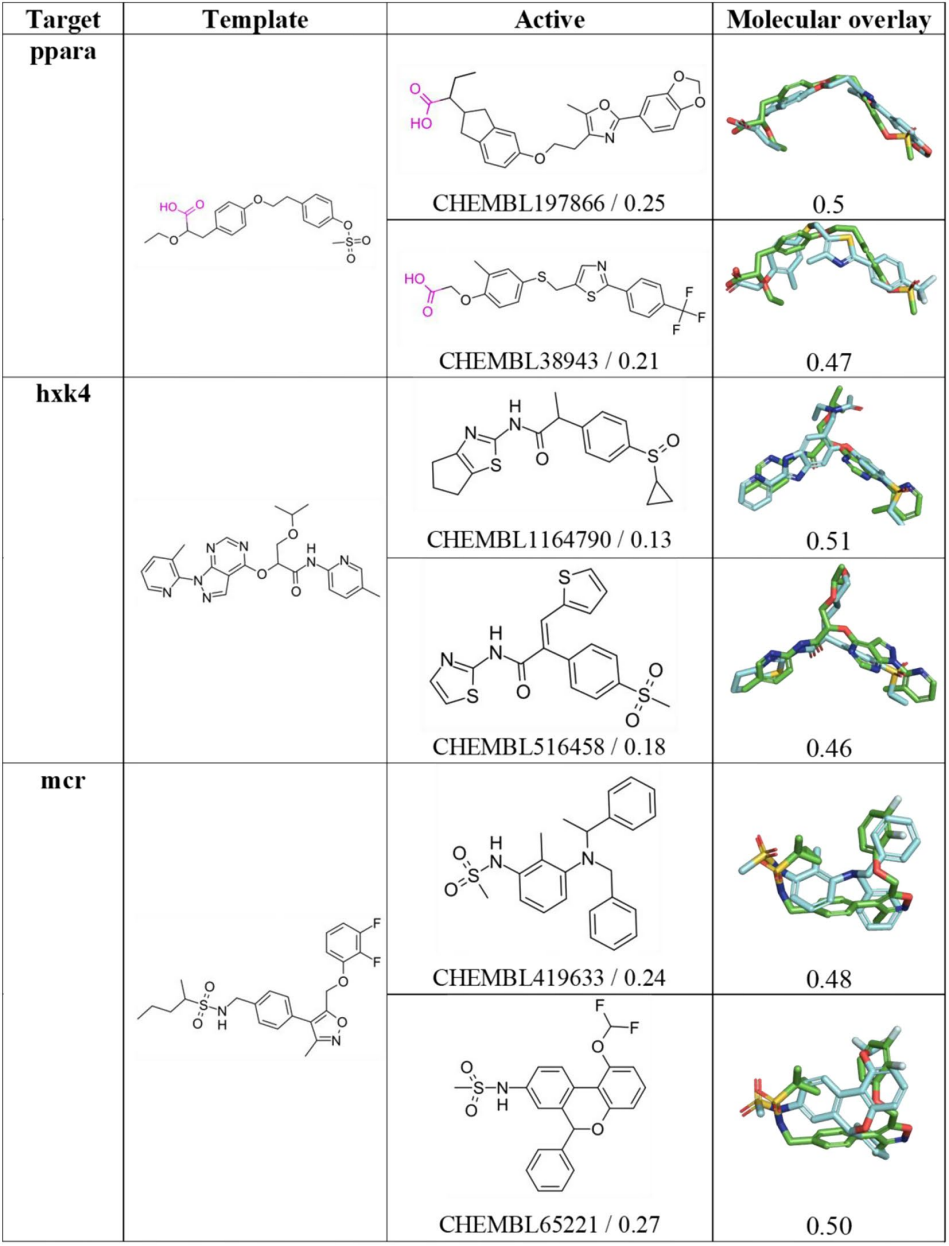
2D Structure of query and top-ranked actives for selected targets where choice of the *Q*_*XR*_. query leads to a better performance in the VS relative to the lowest energy-minimized conformer. For each active compound, the structural resemblance (Tanimoto index using Morgan Fingerprint, radius 2) with the template and the structural overlap between the overlaid *Q*_*LEG*_ query and active are indicated

**Fig. 8:**
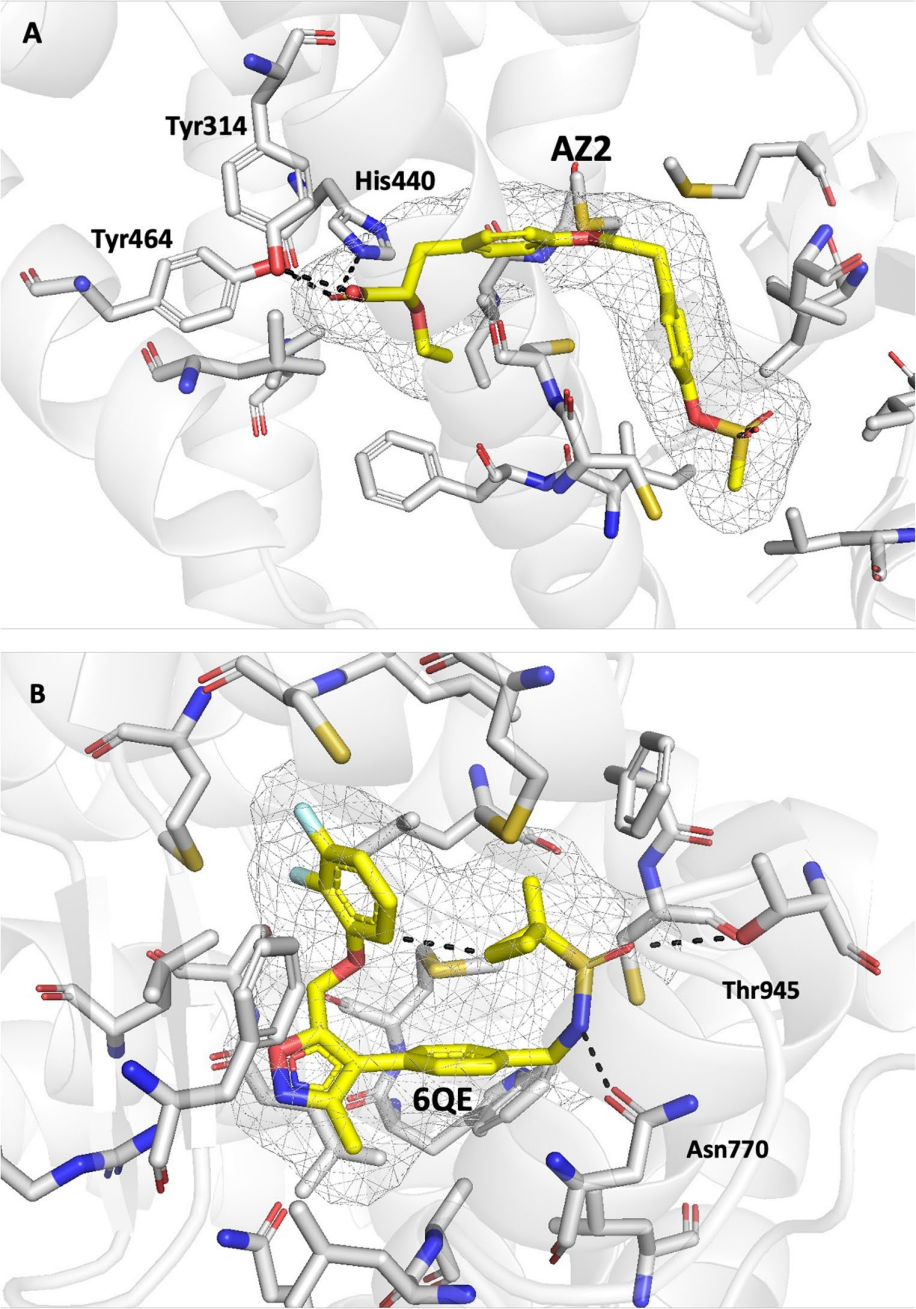
Representation of the bioactive conformation of ligand (**A**) AZ2 and (**B**) 6QE (yellow sticks) found in the X-ray structure of the complex with the binding pocket of the peroxisome proliferator-activated receptors alpha (**ppara**; PDB ID 1I7G) and mineralocorticoid receptor (**mcr**; PDB ID 5L7G), respectively. The shape of the pocket filled by the ligand is shown as a gray wireframe, and selected protein residues are highlighted as sticks

In contrast, the existence of a common scaffold is less evident in the comparison of the chemical skeleton of template and top-ranked actives in cases where the performance of the VS is much larger for the *Q*_*XR*_ query, as noted in the examples shown in Fig. 7. Despite the chemical dissimilarity between template and actives (Tanimoto index < 0.27), the retrieved actives exhibit a notable 3D overlap (close to 0.50) with *Q*_*XR*_. In certain cases, this may be attributed to the adoption of a conformationally strained fold adopted by a flexible template to fit the topological constraints of the binding pocket, as noticed in **ppara**. Here the ligand (AZ2) is anchored through the hydrogen bonds formed by the terminal carboxylate group with residues Tyr314, Tyr464 and His440 in the interior of the binding pocket. The folded bioactive conformation, which is characterized by the normal arrangement of the two benzene rings, reflects the need to fill the L-shaped pocket, which is facilitated by the flexibility of the ethoxy tether (PDB ID 1I7G; see Fig. 8A). A similar situation can be noticed in **mcr**, where the hydrogen bonds formed by the sulfonamide group of the ligand (6QE) with residues Asn770 and Thr945 force the terminal cyclopropyl ring to be oriented toward the difluorobenzene ring located at the other end of the molecule (closest C-C distance of 3.2 Å), thus leading to the strained structure observed in the bioactive conformation (PDB ID 5L7G; see Fig. 8B).

**Fig. 9:**
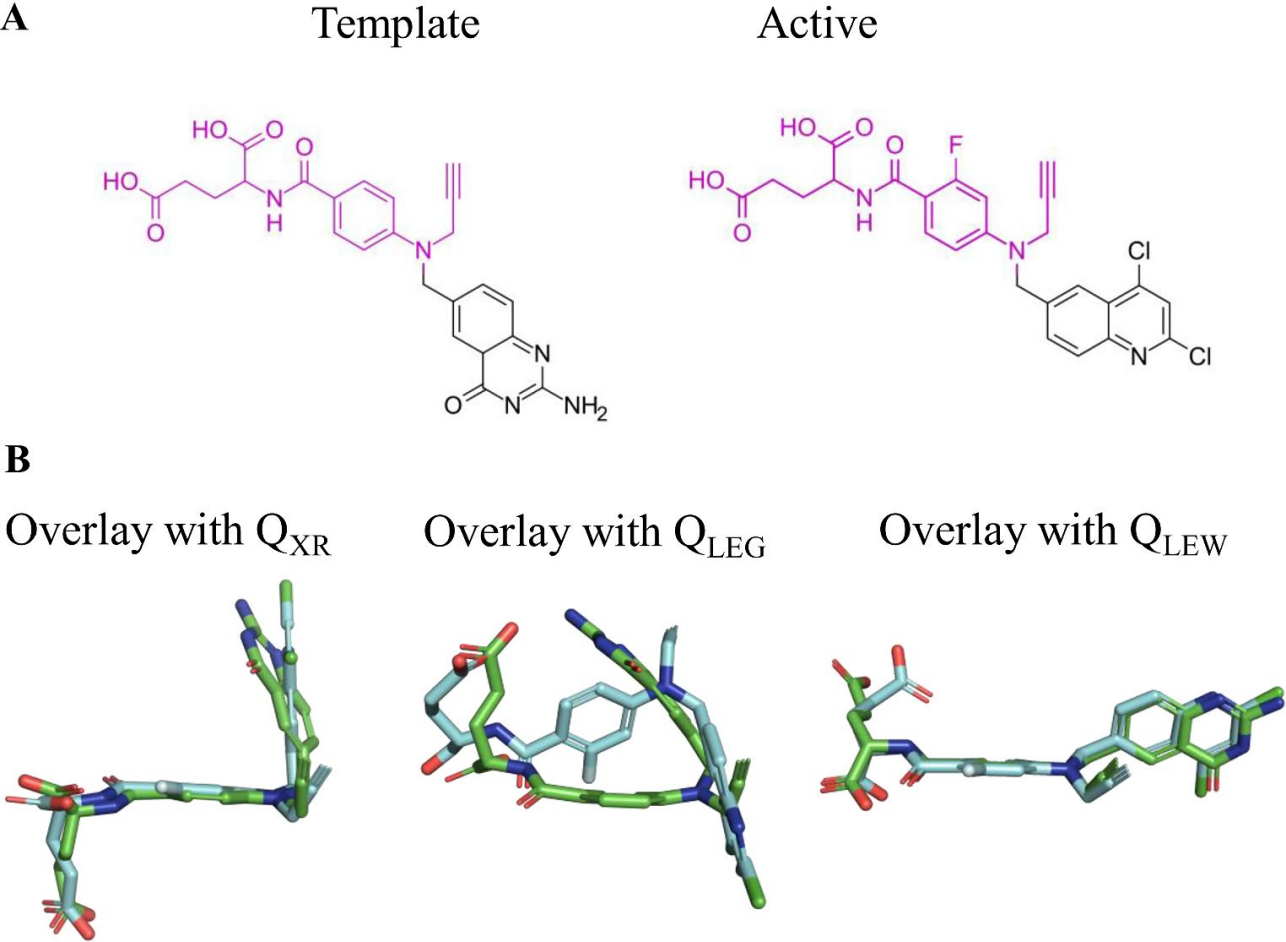
**A**) 2D Structure of template and top-ranked active (CHEMBL-93,048) reported by *Q*_*XR*_ in **tysy**. **B**) Molecular overlay obtained by using *Q*_*XR*_, *Q*_*LEG*_, and *Q*_*LEW*_

Finally, the better performance obtained with *Q*_*XR*_ can be attributed to the bias of the energy minimization in the gas phase to favor the adoption of intramolecular interactions, which are nevertheless absent in the bioactive conformation. This is illustrated by **tysy**: although the template and the top-ranked active share a significant fraction of the chemical scaffold (Fig. 9A), the lowest-energy conformation of the template is remarkably different from the bioactive one due to the stabilization afforded by the electrostatic interaction between the carboxylate group and the exocyclic amino group located at the ends of the compound (Fig. 9B). It is worth noting that this latter case is corrected when the lowest-energy conformation of the template in aqueous solution is considered (Fig. 9B). The almost perfect overlay observed in this latter case reflects the fact that the formation of such an intramolecular contact is penalized by hydration effects, which favor the adoption of an extended conformation that enables the solvent exposure of both carboxylate and amino groups. This finding agrees with earlier studies that noticed the relevance of accounting for solvation effects in the conformational search (see for instance [42]).

Overall, these results indicate that the degree of scaffold similarity between template and actives can modulate the performance of the LBVS depending on the choice of the query conformation. When the template and the actives share a large structural motif in their chemical scaffolds, it is reasonable to expect that the compounds will populate similar conformational spaces, enhancing the chance of finding conformers able to overlap with the template. In this context, the usage of the low-energy query may be better suited for the VS. However, when the structural diversity between template and actives increases, or alternatively when the query conformation is influenced by the formation of specific interactions or steric effects, the 3D information encoded in the X-ray structure may be more adequate for early recovery of the actives.

**Fig. 10:**
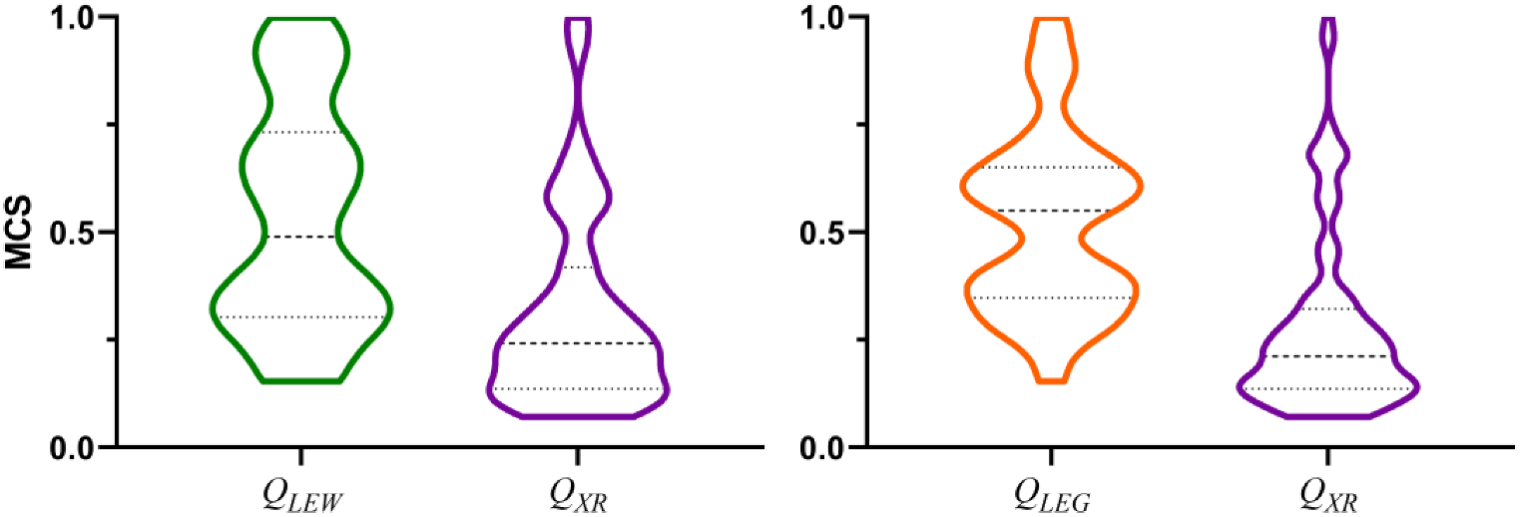
Distribution of the actives recovered in the ROCe 5% for targets **sahh**, **pur2**, **mk01**, **hivrt**, **wee1** and **pa2ga** when either *Q*_*XR*_ (magenta), *Q*_*LEG*_ (orange) or *Q*_*LEW*_ (green) are used as queries. The profiles show the distribution according to the MCS of the actives found uniquely in the VS against *Q*_*LEG*_ */Q*_*LEW*_ but not in *Q*_*XR*_, and viceversa

This interpretation is supported from the analysis of the maximum common substructure (MCS) as a metric to quantify the degree of identity between the chemical scaffolds of template and top-ranked actives. The results obtained for a subset of six targets (**sahh**, **pur2**, **mk01, hivrt**, **wee1** and **pa2ga**) are displayed in Fig. 10. Targets **sahh**, **pur2** and **mk01** were selected since they are characterized by a high 2D similarity between template and top-ranked actives in conjunction with a notable bias in 2D similarity between actives and decoys (Fig. 2A). In contrast, **hivrt**, **wee1** and **pa2ga** were selected because they exhibit low 2D similarity between template and top-ranked actives and a narrow gap between actives and decoys (Fig. 2A). To compare the relation between the query selection and the 2D hit similarity, we analyzed the characteristics of those hits present in the ROCe 5% that were found by only one of the two queries (*Q*_*XR*_ or *Q*_*LEG*_/*Q*_*LEW*_). As discussed above, choice of *Q*_*LEG*_ leads to a better performance in the VS for **sahh**, **pur2** and **mk01**, reflecting the higher MCS found between template and actives (Fig. 10). On the other hand, the outcome of the VS is enhanced when *Q*_*XR*_ is chosen as query for **hivrt**, **wee1** and **pa2ga**, and the actives exhibit a lower MCS with the template (Fig. 10).

### Performance of multiple queries on the virtual screening outcome with pharmScreen

Can the use of multiple queries modulate the influence of the chemical resemblance between template and actives? To answer this question, the previous analysis was extended to the results obtained when an ensemble of conformers of the template are used as query in the VS. Hereafter, the discussion is focused on the results derived using the Parallel fusion algorithm, which led to a slightly better performance (comparison of the results derived for other fusion techniques is provided in Supporting Information Table S7-S9).

**Fig. 11:**
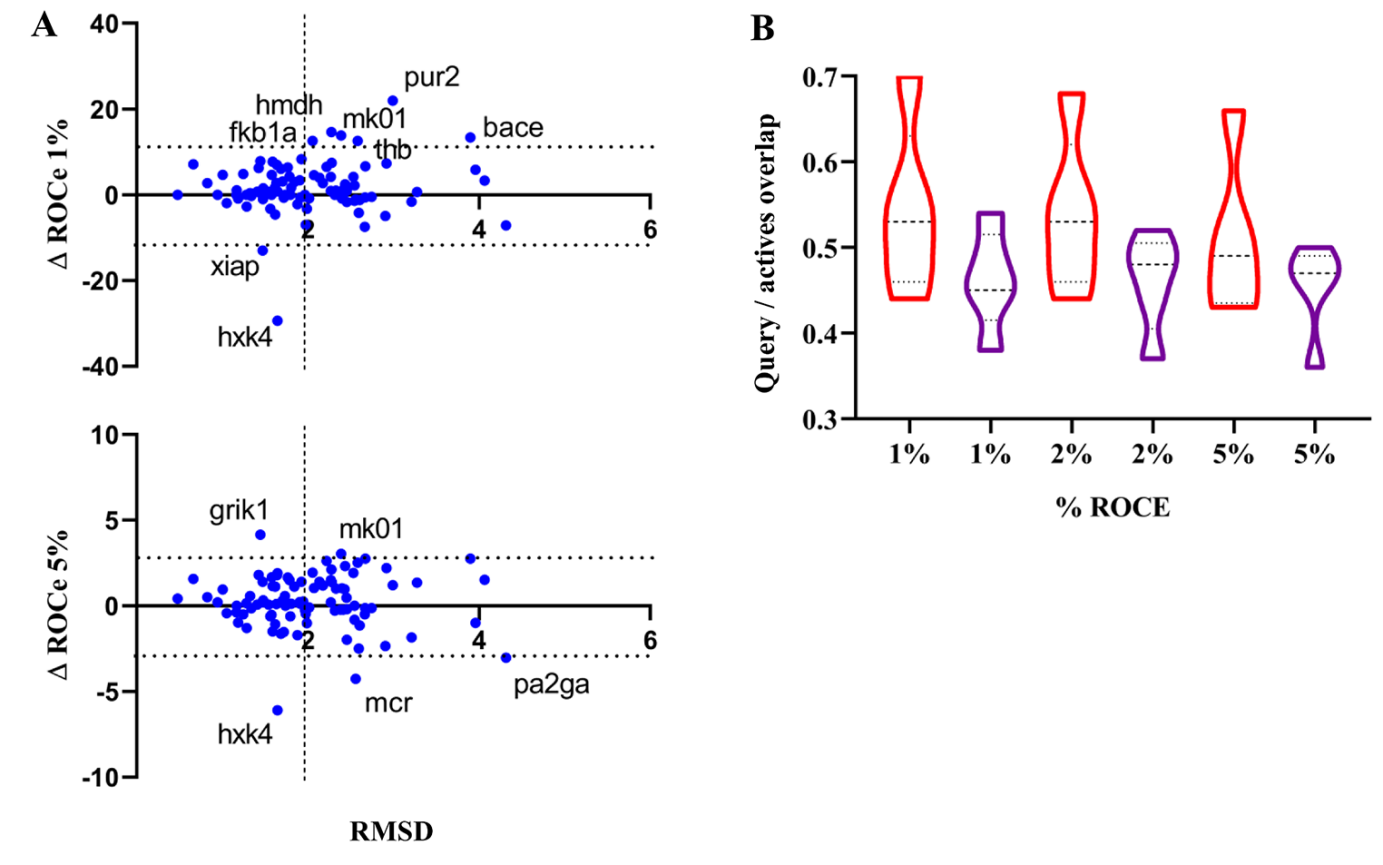
**A**) Representation of the difference in ROCe (ΔROCe) 1% and 5% versus the RMSD averaged for the ensemble of queries (*Q*_*ENS*_) relative to the X-ray structure (*Q*_*XR*_) of the template for the targets in the DUD-E^+^ dataset. **B**) Distribution of the actives uniquely recovered at 1, 2 and 5% according to the structural overlap (0: minimum overlap; 1: maximum overlap) exhibited against the *Q*_*XR*_ (magenta) and *Q*_*ENS*_ (orange) queries. The structural overlap was estimated from the Tanimoto coefficient obtained from the comparison of the projection of the van der Waals radii of atoms onto a grid (spacing of 0.5 Å) of the hydrophobicity-guided alignment of query and actives. Dashed lines denote the average value, and upper/lower dotted lines stand for the 25%/75% of the cases

Although the use of multiple queries has *globally* a minor effect on the outcome of the VS (note the similar ROCe distribution relative to the use of *Q*_*XR*_ in Fig. 3), the representation of the ΔROCe (1% and 5%) values determined relative to the *Q*_*XR*_ conformation versus the RMSD (Fig. 11A) mimics the trends discussed above (see Fig. 5A). The ΔROCe shows similar values (|ΔROCe| < 1) for most of the targets, but differences larger than 10% (ROCe 1%) and 3% (ROCe 5%) are often found, reflecting a better performance when *Q*_*XR*_ is used as query (negative ΔROCe values), as noted for **xiap**, **hxh4**, **mcr** and **pa2ga**, or *Q*_*ENS*_ is otherwise considered (positive ΔROCe values), as observed for **mk01**, **pur2**, **grik1**, and **hmdh** (Table 2). Furthermore, inspection of the distribution of actives recovered at 1, 2 and 5% for this subset of targets consistently exhibits a larger hydrophobic similarity to *Q*_*ENS*_ compared to the hydrophobic overlap determined for the actives retrieved when *Q*_*XR*_ is used in the VS (Fig. 11B).

**Table 2:** ROCe 1 and 5% values obtained for selected targets characterized by large |ΔROCe| values when *Q*_*XR*_ and *Q*_*ENS*_ queries are used in the screening of the DUD-E^+^ dataset

Target	Reference ID Target-Ligand	Q_XR_	Q_ENS_	ΔROCe
ROCe 1%				
bace	2QMD-CS7	18.0	31.5	+ 13.5
fkb1a	1FKH-SBX	31.5	44.1	+ 12.6
hmdh	2R4F-RIE1	46.5	61.2	+ 14.7
mk01	3I5Z-Z48	22.8	36.7	+ 13.9
pur2	4EW3-DXZ	58.0	80.0	+ 22.0
thb	1Q4X-G24	29.1	41.8	+ 12.7
hxk4	4IXC-1JD	35.9	6.5	-33.0
xiap	4HY0-1AQ	25.0	12.0	-13.0
ROCe 5%				
grik1	1VSO-AT11	1.0	5.2	+ 4.2
mk01	3I5Z-Z48	6.3	9.4	+ 3.1
hxh4	4IXC-1JD	10.2	4.1	-6.1
pa2ga	1AYP-INB	7.3	4.2	-3.1
mcr	5L7G-6QE	5.1	0.9	-4.2

**Fig. 12:**
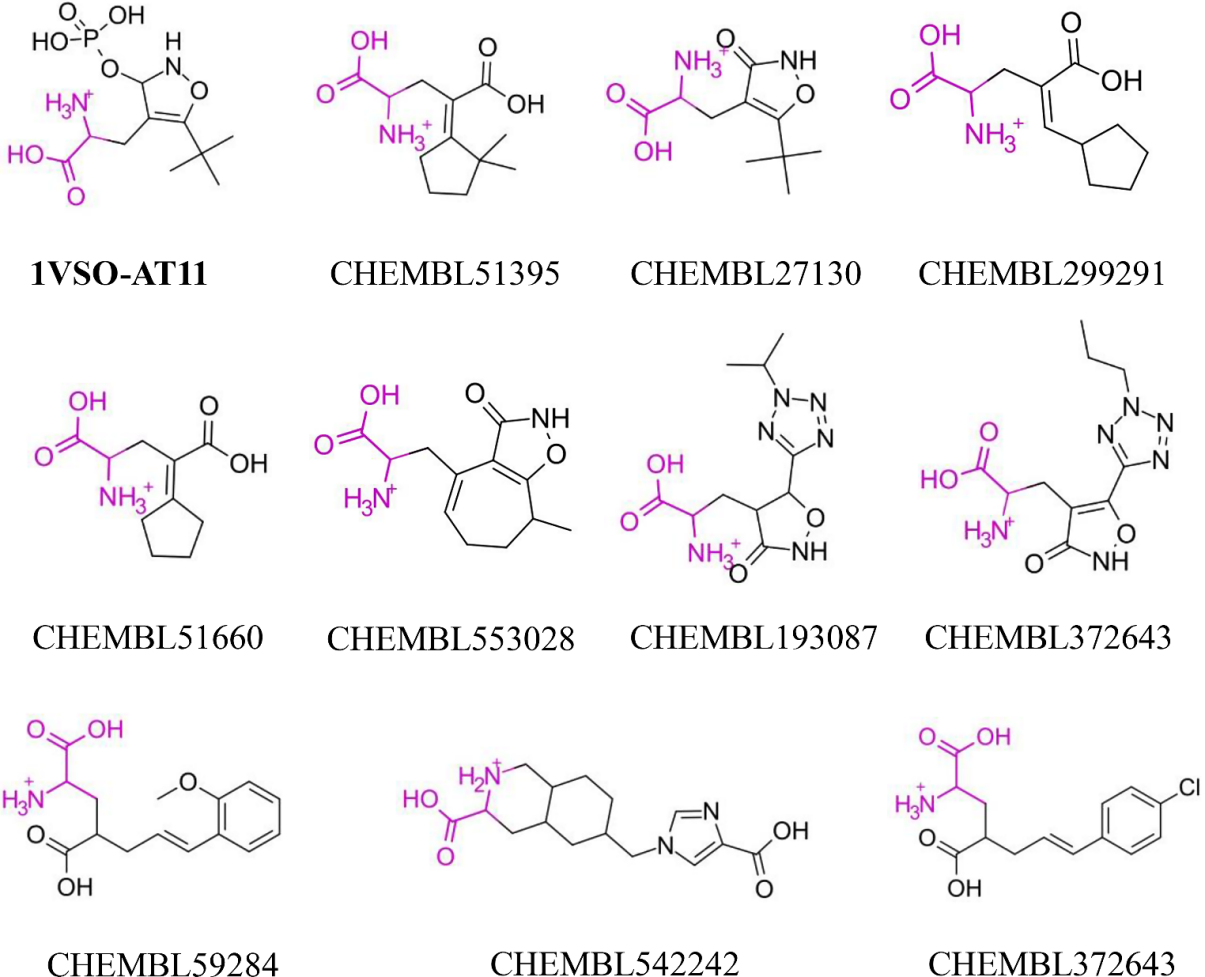
2D structure of query (bold) and top-ranked actives selected upon comparison against *Q*_*ENS*_ for **grik1**

**Fig. 13:**
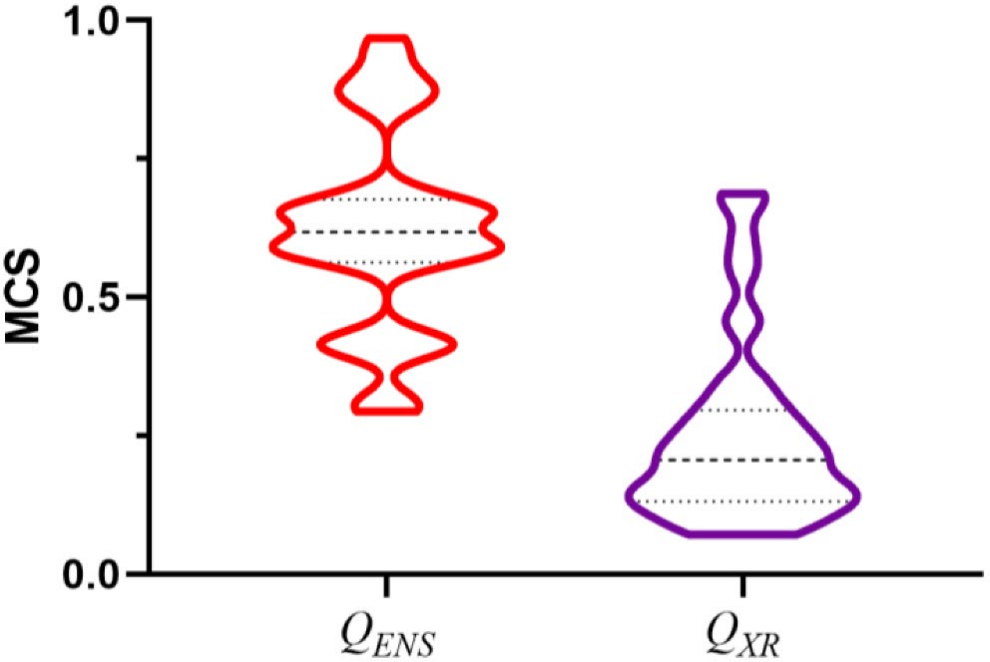
Distribution of the actives recovered in the ROCe 5% for targets **sahh**, **pur2**, **mk01**, **hivrt**, **wee1** and **pa2ga** when either *Q*_*ENS*_ (red) or *Q*_*XR*_ (magenta) are used as queries. The profiles show the distribution obtained according to the MCS of the actives found uniquely in the VS against *Q*_*ENS*_ but not in *Q*_*XR*_, and viceversa

As an example, the template of **grik1** includes an alanine group, which is present in the 10 top-ranked actives recovered in the VS performed using the *Q*_*ENS*_ query (Fig. 12). In fact, the VS results suggest that the performance obtained using *Q*_*XR*_ or *Q*_*ENS*_ is affected by the degree of scaffold similarity between template and actives (Fig. 13). When the 2D similarity is high, the actives adopt conformations that resemble the conformers included in the template’s ensemble. However, if the common structure shared between template and actives is low, the X-ray structure may be better suited for early recovery of the actives.

As a final remark, let us note a distinctive trait observed in the comparison of the *Q*_*ENS*_ query and the lowest-energy conformation (*Q*_*ENS*_; see Fig. S3 in Supporting Information). A similar performance is generally found for most targets. Nevertheless, in few cases, especially **ppara** and **tysy**, differences larger than 10% (ROCe 1%) and 3% (ROCe 5%) are found with the use of *Q*_*ENS*_. Thus, the choice of the ensemble query may be valuable to correct the limitations found for *Q*_*LEG*_ (see discussion above).

### Impact of query conformation on the virtual screening outcome with phase shape

The performance of the VS may be influenced by the nature of the molecular descriptors used to measure the similarity between template and actives/decoys. Whereas the preceding discussion relies on the usage of the QM-derived Hyphar descriptors from PharmScreen, a complementary analysis was performed resorting to Phase-based shape descriptors. For the sake of comparison with the results shown above, this analysis is limited to *Q*_*XR*_ and *Q*_*LEG*_. The summary of the AUC and ROCe results obtained for the whole set of targets is provided in Supporting Information Table S10, and results for individual sets in Supporting Information Table S11.

**Fig. 14:**
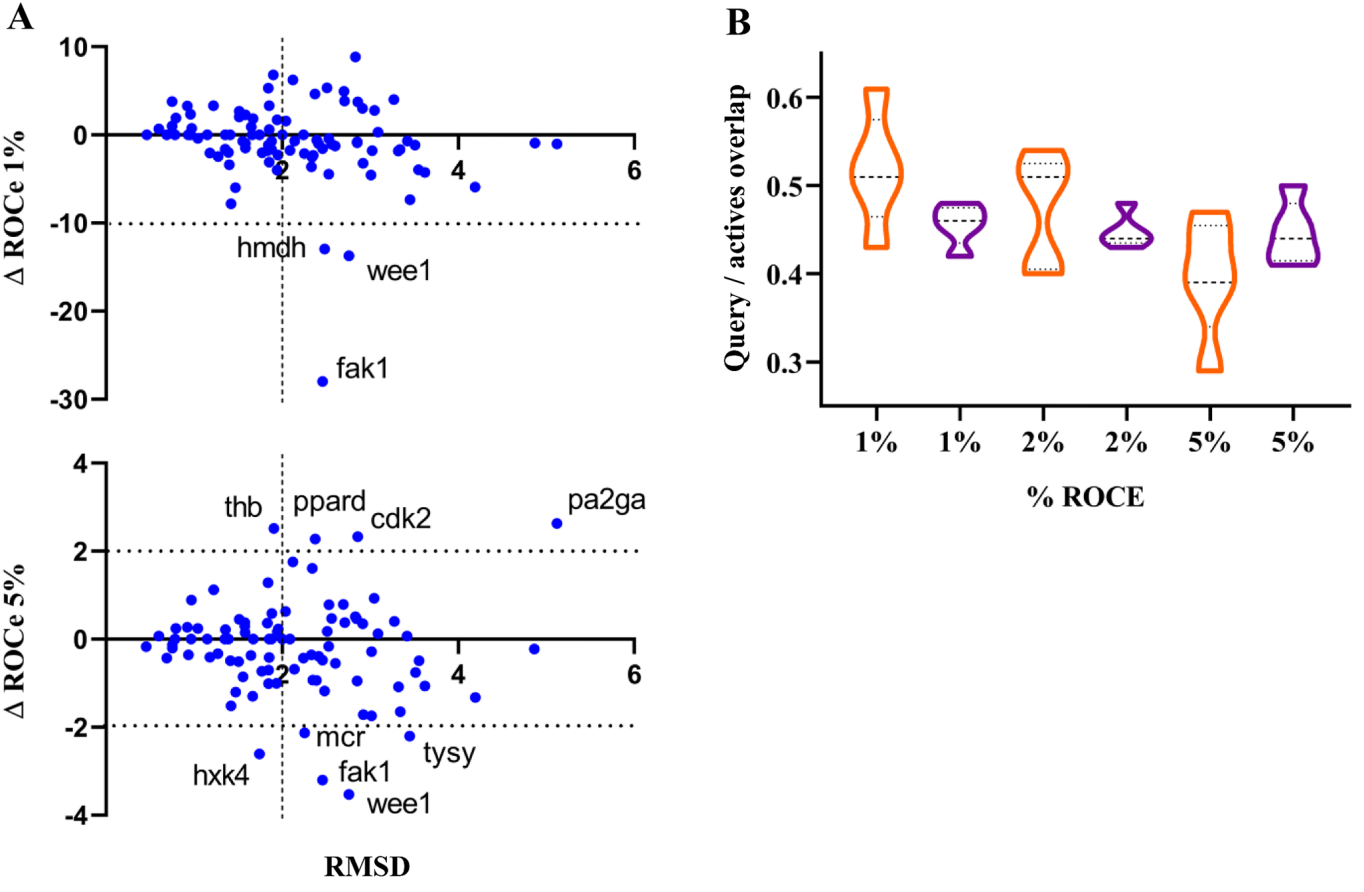
**A**) Representation of the difference in ROCe (ΔROCe) 1% and 5% versus the RMSD between the *Q*_*LEG*_ query conformation relative to the X-ray crystallographic one (*Q*_*XR*_) for the targets included in the DUD-E^+^ dataset. **B**) Distribution of the actives uniquely recovered at 1, 2 and 5% according to the structural overlap (0: minimum overlap; 1: maximum overlap) exhibited against the *Q*_*XR*_ (magenta) and *Q*_*LEG*_ (orange) queries. The structural overlap was estimated from the Tanimoto coefficient obtained from the comparison of the projection of the van der Waals radii of atoms onto a grid (spacing of 0.5 Å) of the Phase shape-guided alignment of query and active. Dashed lines denote the average value, and upper/lower dotted lines stand for the 25%/75% of the cases

**Fig. 15:**
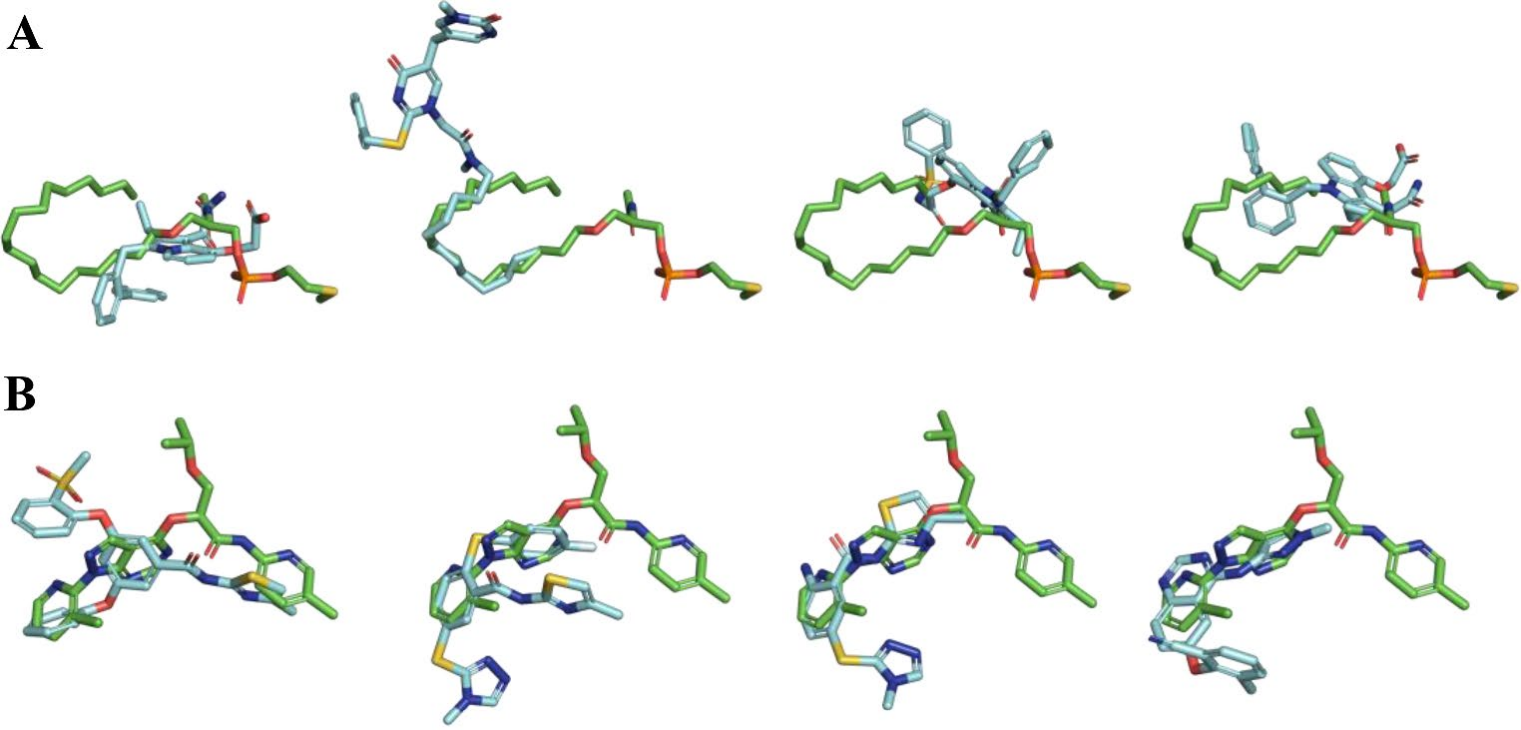
Alignment between *Q*_*XR*_ and a subset of chemically diverse actives selected using Phase Shape and characterized by low overlap with *Q*_*XR*_. **A**) Target **pa2ga** (from left to right): CHEMBL356606, CHEMBL158383, CHEMBL514692, and CHEMBL347957. **B**) Target **hxk4** (from left to right): CHEMBL6475058, CHEMBL551445, CHEMBL48514, and CHEMBL572840

Inspection of the distribution profiles shown in Fig. 14A, which represents the ΔROCe (1% and 5%) values determined between *Q*_*LEG*_ and *Q*_*XR*_ queries versus the RMSD between these conformations, reproduces the general trends obtained with the Hyphar hydrophobic descriptors. Positive and negative ΔROCe values denote targets where the use of *Q*_*LEG*_ or *Q*_*XR*_ leads to a better performance. The former case is observed for targets such as **thb**, **cdk2**, **ppard** and **pa2ga**, whereas the latter case is found for **wee1**, **fak1**, **mcr**, **tysy**, **hxh4** and **hmdh** (Table 3). Furthermore, the distribution of actives recovered at 1% and 2% for this subset of targets exhibits a larger shape-based similarity to *Q*_*LEG*_ compared to the shape overlap determined for the actives retrieved when *Q*_*XR*_ is used as query (Fig. 14B). Note, however, that this trait is not reflected in the distribution obtained at ROCe 5%. This can be ascribed to the fact that the alignments between template and actives exhibit a low 3D overlap for certain actives with a high degree of chemical diversity relative to the template, as noticed for targets **pa2ga** (Fig. 15A) and **hxk4** (Fig. 15B).

**Table 3:** ROCe 1 and 5% values obtained for selected targets characterized by large |ΔROCe| values when *Q*_*XR*_ and *Q*_*LEG*_ queries are used in the screening of the DUD-E^+^ dataset

Target	Reference ID Target-Ligand	Q_XR_	Q_LEG_	ΔROCe
ROCe 1%				
fak1	4GU6-10N1	47.0	19.0	-28.0
hmdh	2R4F-RIE1	53.5	40.6	-12.9
wee1	5VD4-99 J	31.1	23.2	-7.9
ROCe 5%				
cdk2	1OGU-ST8	2.9	5.2	+ 3.3
pa2ga	1AYP-INB	0.6	3.2	+ 2.6
ppard	5U3Z-7UA	0.5	2.8	+ 2.3
thb	1Q4X-G24	4.5	7.0	+ 3.5
fak1	4GU6-10N1	14.0	10.8	-3.2
hxh4	4IXC-1JD	5.0	2.4	-2.6
mcr	5L7G-6QE	4.3	2.1	-2.2
tysy	1TRG-CB3	13.4	11.2	-2.2
wee1	5VD4-99 J	5.7	2.2	-3.5

### Impact of query conformation in finding novel scaffolds (DUD-E^+^-diverse)

To further check the influence of the structural bias between actives and decoys relative to the template, the DUD-E^+^-Diverse database was designed to select targets where (i) actives have low structural resemblance to the template, (ii) both actives and decoys have comparable 2D structural similarities to the template (Fig. 2B), and ii) there is an appropriate ratio between the number of decoys and actives in the dataset (Supporting Information Table S2). The AUC and ROCe values obtained for the results of the 15 targets included in DUD-E^+^-Diverse are shown in Supporting Information Table S12 (results for individual targets are given in Supporting Information Tables S13 and S14).

**Fig. 16:**
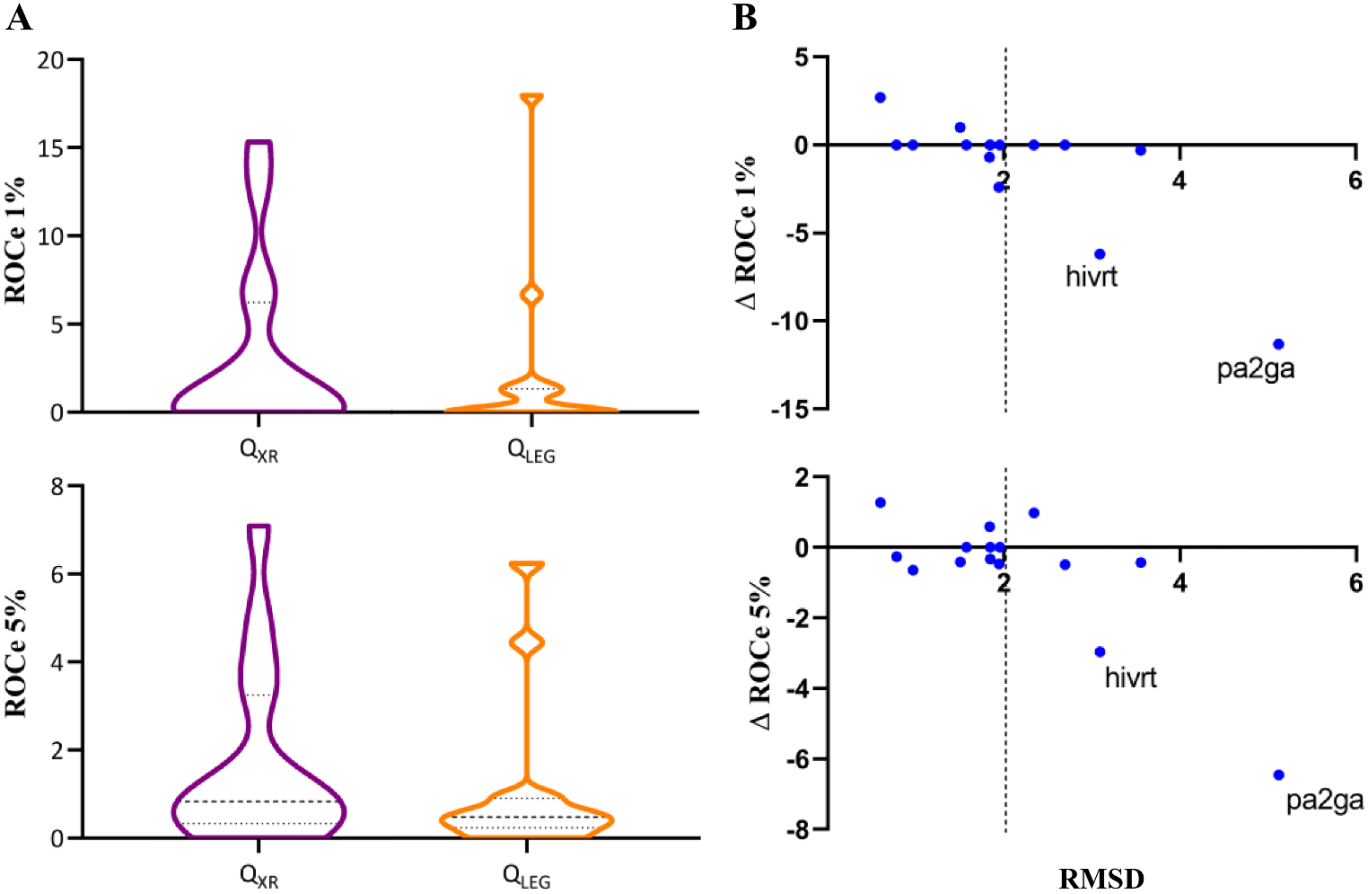
**A**) Distribution of ROCe values (top: 1%; bottom: 5%) determined for the set of targets included in DUD-E^+^-Diverse dataset using *Q*_*XR*_ and *Q*_*LEG*_ query definitions for the template. **B**) Representation of the difference in ROCe (ΔROCe) 1% and 5% versus the RMSD between the *Q*_*LEG*_ query conformation relative to the X-ray crystallographic one (*Q*_*XR*_) for the targets included in the DUD-E^+^ dataset

Compared to Figs. 4 and 16A shows larger differences in the shape of the ROCe 1% and 5% distribution profiles, which are however not statistically significant because of the low number of targets with high RMSD differences. Figure 16B shows that similar ROCe values are obtained for all the targets but **hivrt** and **pa2ga**, where the use of *Q*_*XR*_ gives a better performance.

**Fig. 17:**
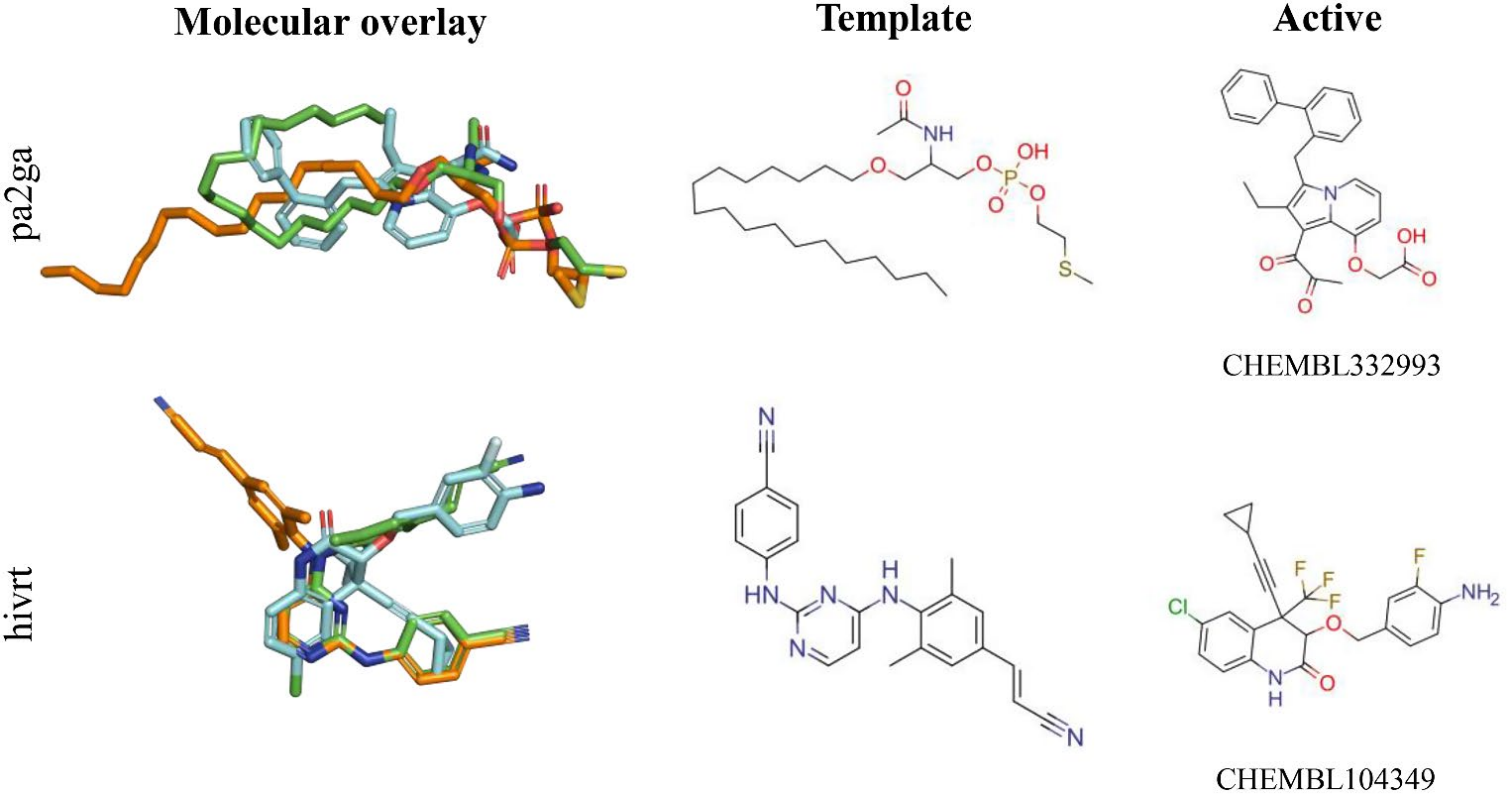
PharmScreen alignment of *Q*_*XR*_ /*Q*_*LEG*_ (green/orange) with a top-ranked active (cyan) for **pa2ga** and **hivrt**

With regard to **pa2ga**, the ROCe values obtained using *Q*_*XR*_ (ROCe 1%: 12.9; 5%: 7.1) outperforms those obtained with *Q*_*LEG*_ (ROCe 1%: 1.6; 5%: 0.7). This can be related to the adoption of a folded structure of the long, flexible aliphatic chain in the binding pocket, which favors the retrieval of actives such as CHEMBL332993 (Fig. 17). In **hivrt** (ROCe 1% 6.2; 5%: 3.3 for *Q*_*XR*_, and 0.0 and 0.3, respectively, for *Q*_*LEG*_) the bioactive structure shows a closed structure, which enables the recovery of CHEMBL104349, whereas *Q*_*LEG*_ fails in finding active compounds (Fig. 17). Overall, these results support the better performance of the *Q*_*XR*_ query in cases where the bioactive conformation of the template can be altered upon binding to the protein target, as noticed for **pa2ga** and **hivrt**.

### Final remarks

The global analysis of the results reveals that the performance, on average, of the 3D LBVS has generally a mild dependence on the choice of the template’s query conformation, which agrees with the results reported in previous works [20–23]. This tendency can be attributed to a large extent to the fact that the RMSD between the bioactive (X-ray) structure of the ligand and low-energy conformers is in most cases lower than 2.5 Å for around 70% of the targets. Nonetheless, the results obtained for specific targets also reveals noticeable differences in the performance of the screening depending on the template’s conformation used as query, underscoring the significance of 3D data.

As a general trait, in cases where the actives exhibit a notable structural similarity with the chemical scaffold of the template, choice of the energy-minimized conformation can be better suited. This can be attributed to the presence of a common chemical scaffold between template and active, which should favor the adoption of similar conformational spaces, and hence the recovery of actives that exhibit a significant 3D structural overlap with the template. Nevertheless, this scenario may be challenged by different factors, such as the introduction of conformational strain in the template upon binding to the protein target, either arising from the steric constraints imposed by the topology of the binding pocket, or the formation of specific interactions with target residues that compensate the energetic penalty required for adopting a conformation distinct from the lowest-energy conformer, as discussed before for **ppara** and **mcr**.

When these factors are important in the screening against templates endowed with large conformational flexibility, the usage of the X-ray structure can be expected to be better suited than the lowest-energy conformer to enhance the early recovery of actives, since the bioactive conformation encodes information relative to the molecular determinants that must be fulfilled by the ligands to achieve a proper binding in the target pocket, as discussed for **pa2ga** and **hivrt**.

An alternative scenario that may affect the appropriate choice of the query conformation is the reliability of the computational protocol adopted to generate the query conformation. Computational efficiency may favor the implementation of energy minimization in the gas phase, but this may lead to the formation of artifactual intramolecular interactions due to the neglect of the solvation effects, which may bias the outcome of VS, as noted in **tysy**. In this case, comparison of the energy-minimized structures in the gas phase and in aqueous solution may shed light into the robustness of the query conformer, favoring the choice of the experimental conformation when notable discrepancies are observed between the low-energy structures in gas phase and in solution.

Finally, the usage of an ensemble of conformers as a multiple query does not seem *a priori* to be an efficient approach to alleviate the bias originated from the structural similarity between template and actives, especially keeping in mind the concomitant increase in the computational cost. Nevertheless, a careful analysis of (i) the constraints posed by neighboring residues in the binding pocket on the structure of the template, and (ii) the influence of hydration on the population of conformational families may be valuable to envisage the definition of a multiple query that combines specific (*Q*_*XR*_, *Q*_*LEG*_ and *Q*_*LEW*_) conformations to enhance the retrieval of actives in the VS. This issue will be analyzed in future studies.

Overall, this study shows that the choice of the query may affect the recovery of actives for certain targets, especially when there is large chemical diversity between templates and actives, in order to tune the merits of novel 3D-based methodologies to screening campaigns.

## Electronic supplementary material

Below is the link to the electronic supplementary material.

Supplementary Material 1

## Data Availability

Data availability All data and material are available in the supplementary information.DUD-E+-Diverse is available in https://github.com/Pharmacelera/Query-models-to-3DLBVS.

## Declarations

### Employment

JV and EH are employees of Pharmacelera S.L.

### Supporting Information

The python script to perform the clustering in the ensemble query (*Q*_*ENS*_) and the DUD-E^+^-Diverse set (SMILES codes for the actives, decoys, and query) are available in the public GitHub repository at https://github.com/Pharmacelera/Query-models-to-3DLBVS.
