# Supplementary material for: On the relevance of query definition in the performance of 3D ligand-based virtual screening
Source: J Comput Aided Mol Des. 2024 Apr 4;38(1):18. doi: 10.1007/s10822-024-00561-5 (PMC10995064; doi:10.1007/s10822-024-00561-5)
Supplement: Supplementary file 1 — Supplementary Material 1 [file 10822_2024_561_MOESM1_ESM.docx]

**Supporting Information**

**On the relevance of query definition in the performance of 3D ligand-based virtual screening**

Javier Vázquez,^*, 1, 2^ Ricardo García,^1^ Paula Llinares,^1, 2^ F. Javier Luque^2^ and Enric Herrero^1^

1 Pharmacelera, Parc Científic de Barcelona (PCB), C/ Baldiri Reixac 4-8, Barcelona E-08028, Spain

2 Departament de Nutrició, Ciències de l’Alimentació i Gastronomia, Facultat de Farmàcia i Ciències de l’Alimentació, Institut de Biomedicina (IBUB) and Institut de Química Teòrica i Computacional (IQTC-UB), University of Barcelona, Av. Prat de la Riba 171, Santa Coloma de Gramenet E-08921, Spain


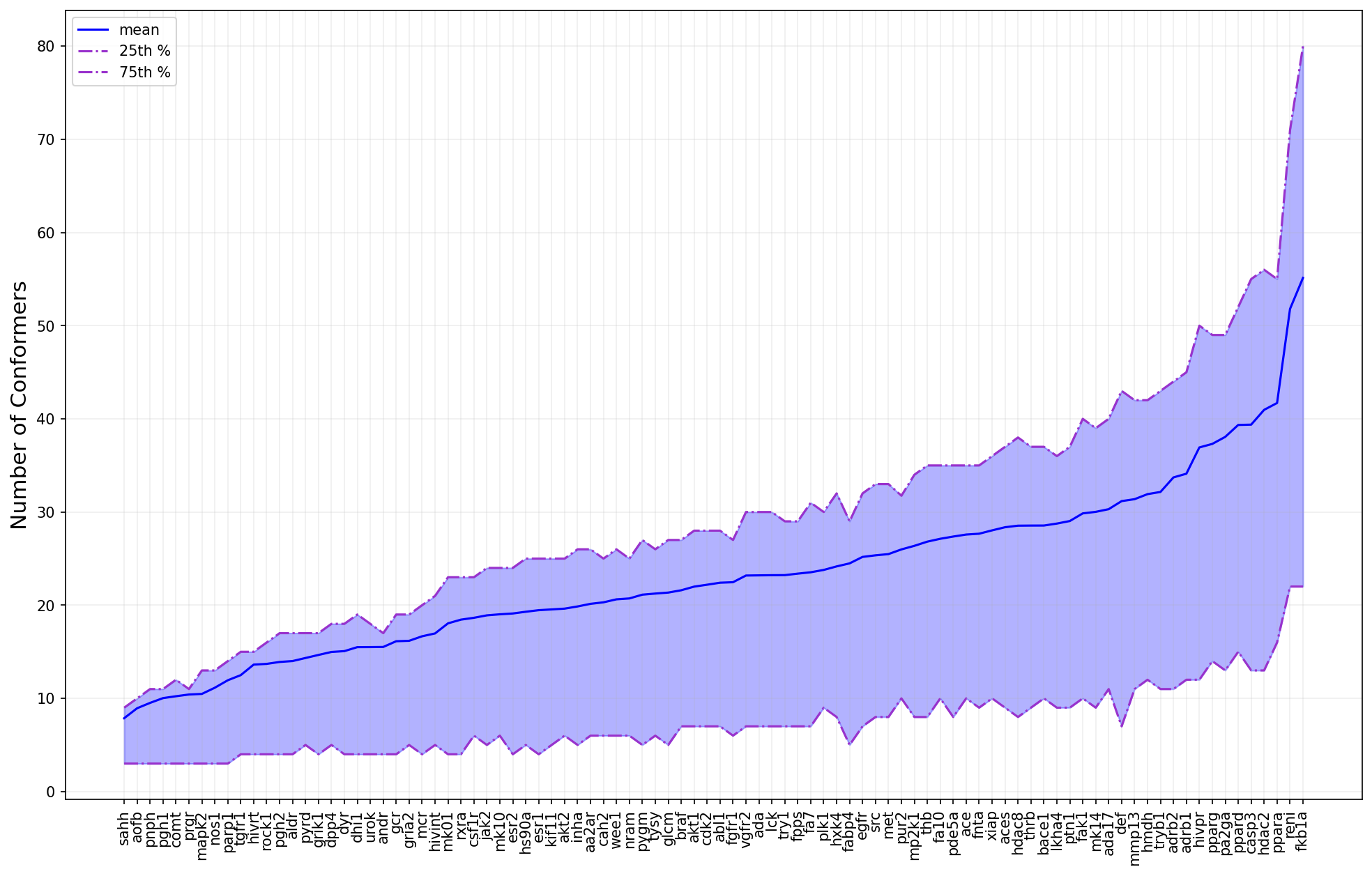


**Fig. S1** Distribution (mean: solid line; 25th and 75th percentile: lower and upper lines) of the number of 3D conformers generated for the set of targets included in the DUD-E^+^ library.

**
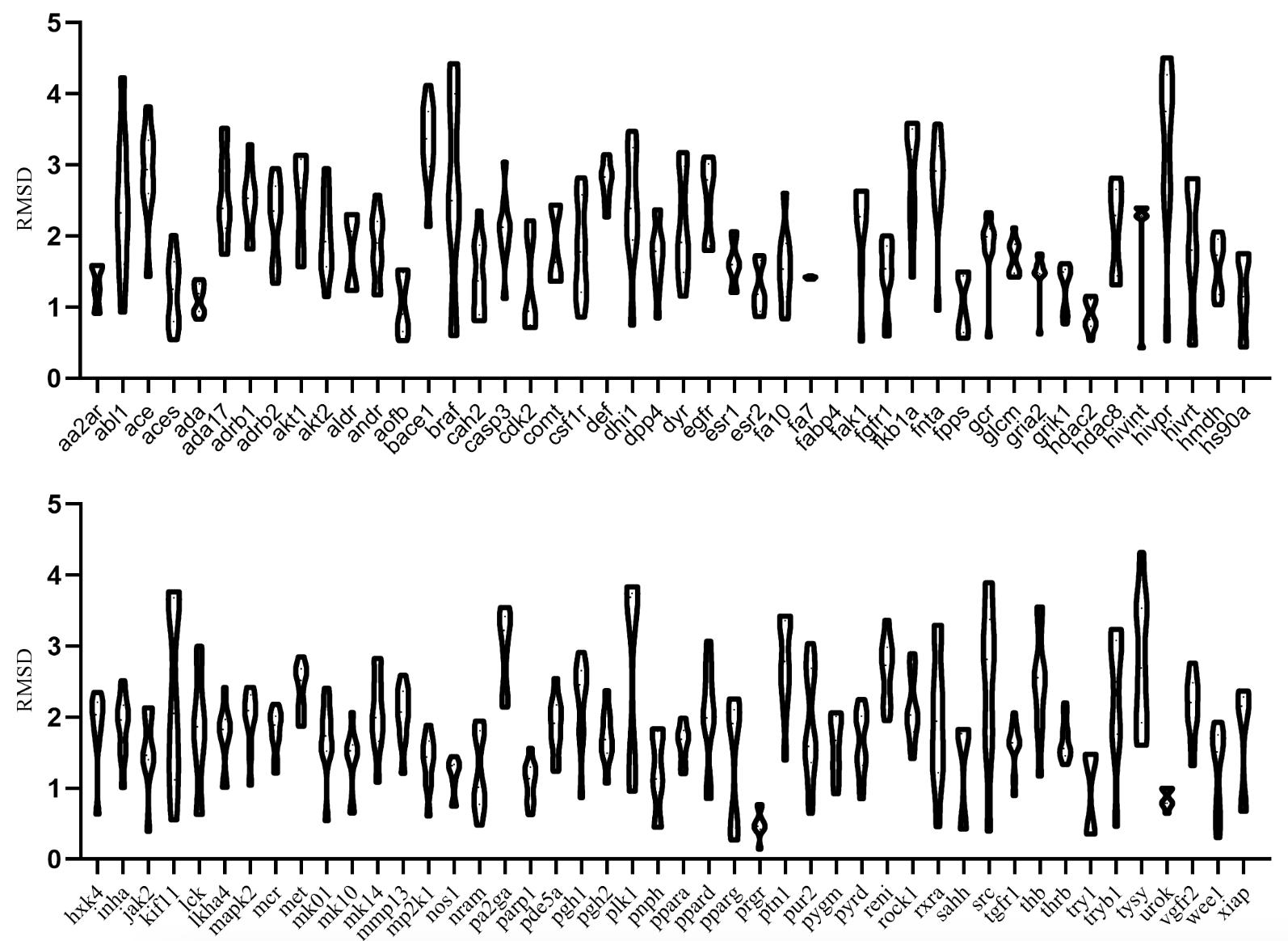
**

| **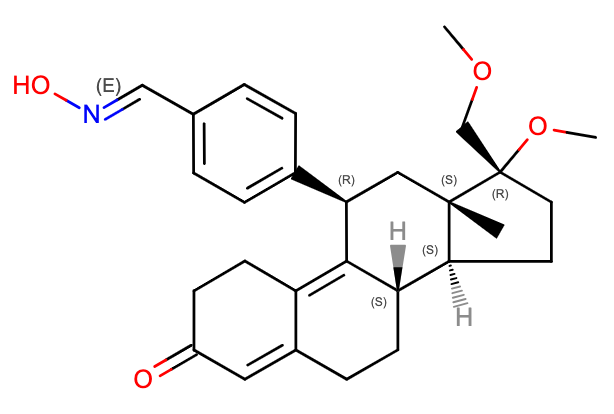** | **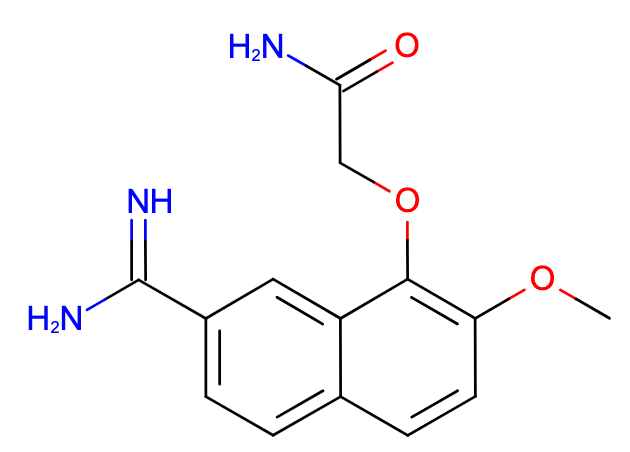** |
| --- | --- |
| AS0 | 1UP |

**Fig. S2** (Top) Distribution of the RMSD values determined between the distinct conformations included in the *Q_ENS_* query generated for the template in each target. (Bottom) representation of ligands with limited conformational flexibility: AS0 (**prgr**) and 1UP (**urok**).

**
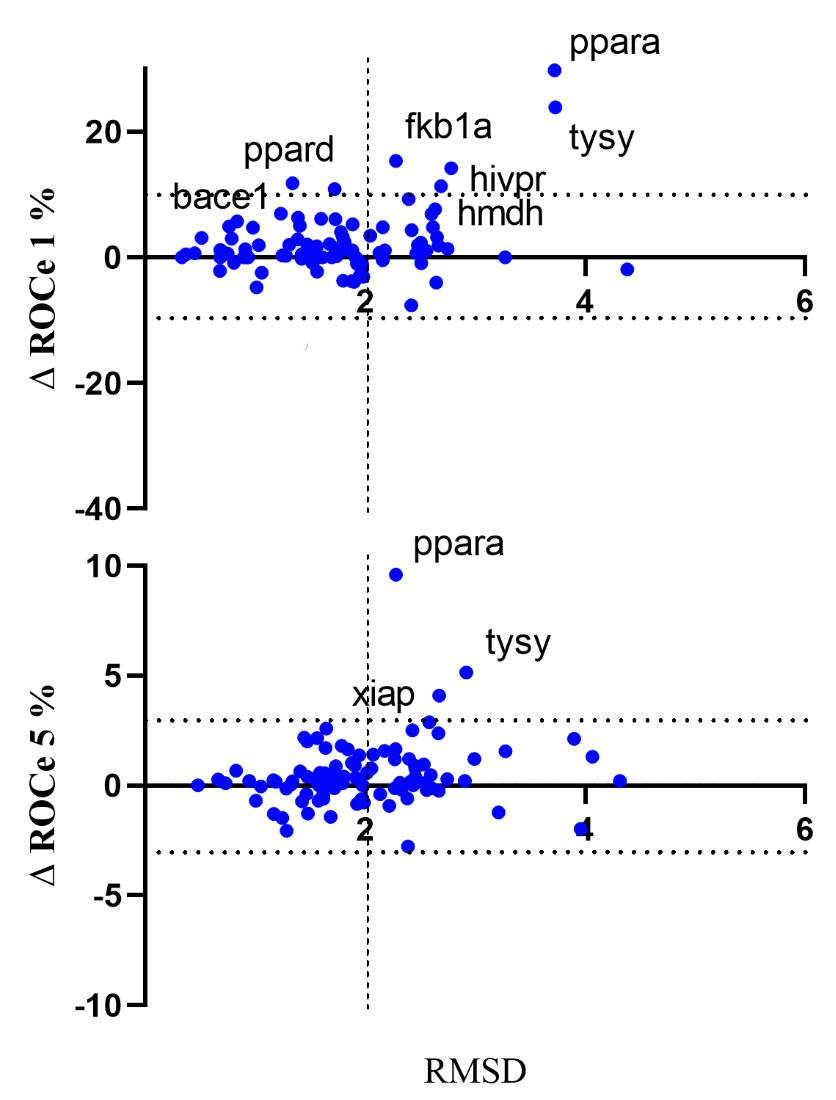
**

**Fig. S3** Representation of the difference in ROCe (ΔROCe) 1% and 5% versus the RMSD averaged for the ensemble of queries (*Q_ENS_*) relative to the low-energy conformation (*Q_LEG_*) of the template for the targets in the DUD-E^+^ dataset.

**Table S1.** List of selected DUD-E^+^ queries for single compound evaluation.

| **Target** | **Reference ID**  Target - Ligand | **Number of actives** | **Decoy-to-active ratio** |
| --- | --- | --- | --- |
| **aa2ar** | 5K2D-ZMA | 482 | 65 |
| **abl1** | 2F4J-VX6 | 182 | 59 |
| **ace** | 6F9U-D0W | 282 | 60 |
| **aces** | 2CMF-F11 | 453 | 58 |
| **ada** | 1WXY-FRK | 93 | 59 |
| **ada17** | 3EDZ-INN | 532 | 67 |
| **adrb1** | 4AMJ-CVD | 247 | 64 |
| **adrb2** | 3P0G-P0G | 231 | 65 |
| **akt1** | 3QKL-SMR | 293 | 56 |
| **akt2** | 2JDO-I5S | 117 | 59 |
| **aldr** | 4LAZ-1WW | 159 | 57 |
| **andr** | 2AXA-FHM | 269 | 53 |
| **aofb** | 2V5Z-SAG | 122 | 57 |
| **bace1** | 2QMD-CS7 | 283 | 64 |
| **braf** | 3OG7-032 | 152 | 65 |
| **cah2** | 1BN4-AL9 | 492 | 63 |
| **casp3** | 4QU9-ACE | 199 | 54 |
| **cdk2** | 1OGU-ST8 | 474 | 59 |
| **comt** | 4XUD-43H | 41 | 94 |
| **csf1r** | 3DPK-8C5 | 166 | 73 |
| **def** | 1ICJ-2PE | 102 | 56 |
| **dhi1** | 3FRJ-A491 | 330 | 59 |
| **dpp4** | 2HHA-3TP | 533 | 77 |
| **dyr** | 3FS6-DH1 | 231 | 74 |
| **egfr** | 3BEL-POX | 542 | 65 |
| **esr1** | 2R6Y-LLC1 | 383 | 54 |
| **esr2** | 2FSZ-OHT | 367 | 55 |
| **fa10** | 2XBX-RR8 | 537 | 53 |
| **fa7** | 2F9B-N1H | 114 | 55 |
| **fabp4** | 5HZ8-65Z | 47 | 59 |
| **fak1** | 4GU6-10N1 | 100 | 54 |
| **fgfr1** | 4RWJ-66T | 139 | 63 |
| **fkb1a** | 1FKH-SBX | 111 | 52 |
| **fnta** | 2IEJ-S48 | 592 | 87 |
| **fpps** | 1YV5-RIS1 | 85 | 104 |
| **gcr** | 3E7C-866 | 258 | 58 |
| **glcm** | 2V3E-NND | 54 | 70 |
| **gria2** | 3H03-UBP1 | 158 | 75 |
| **grik1** | 1VSO-AT11 | 101 | 65 |
| **hdac2** | 4LY1-20Y | 185 | 56 |
| **hdac8** | 3SFF-0DI | 170 | 61 |
| **hivint** | 5KGW-7SK | 100 | 67 |
| **hivpr** | 1EBZ-BEC | 536 | 67 |
| **hivrt** | 6ELI-T27 | 338 | 56 |
| **hmdh** | 2R4F-RIE1 | 170 | 51 |
| **hs90a** | 1UY9-PU6 | 88 | 55 |
| **hxk4** | 4IXC-1JD | 92 | 51 |
| **inha** | 5CPF-53K2 | 43 | 53 |
| **jak2** | 5WIM-35R | 107 | 61 |
| **kif11** | 4A5Y-G7X | 116 | 59 |
| **lck** | 2OF2-547 | 420 | 65 |
| **lkha4** | 3FTX-BES | 171 | 55 |
| **mapk2** | 2PZY-B18 | 101 | 61 |
| **mcr** | 5L7G-6QE | 94 | 55 |
| **met** | 5HTI-66L | 166 | 68 |
| **mk01** | 3I5Z-Z48 | 79 | 58 |
| **mk10** | 4Y5H-519 | 104 | 63 |
| **mk14** | 3D83-GK6 | 578 | 62 |
| **mmp13** | 3ELM-24F | 572 | 65 |
| **mp2k1** | 3DV3-MEK1 | 121 | 67 |
| **nos1** | 1QWC-H4B | 100 | 81 |
| **nram** | 1B9V-RA2 | 98 | 63 |
| **pa2ga** | 1AYP-INB | 99 | 52 |
| **parp1** | 4R6E-3JD | 508 | 59 |
| **pde5a** | 3TGE-TGE | 398 | 69 |
| **pgh1** | 2OYE-IM8 | 195 | 55 |
| **pgh2** | 5W58-FF81 | 435 | 53 |
| **plk1** | 4A4L-939 | 107 | 64 |
| **pnph** | 4EAR-IM52 | 103 | 67 |
| **ppara** | 1I7G-AZ2 | 373 | 52 |
| **ppard** | 5U3Z-7UA | 240 | 51 |
| **pparg** | 5YCP-BRL | 484 | 52 |
| **prgr** | 2OVM-AS0 | 293 | 53 |
| **ptn1** | 1NL9-989 | 130 | 56 |
| **pur2** | 4EW3-DXZ | 50 | 54 |
| **pygm** | 1WV0-BN41 | 77 | 51 |
| **pyrd** | 3KVJ-1X5 | 111 | 58 |
| **reni** | 2V16-C472 | 104 | 67 |
| **rock1** | 5KKS-6U1 | 100 | 63 |
| **rxra** | 4RMD-3SW | 131 | 53 |
| **sahh** | 3NJ4-AFX3 | 63 | 55 |
| **src** | 2OIQ-STI | 524 | 66 |
| **tgfr1** | 2X7O-ZOP | 133 | 64 |
| **thb** | 1Q4X-G24 | 103 | 72 |
| **thrb** | 2C8Y-C3M | 461 | 59 |
| **try1** | 1O3D-780 | 449 | 58 |
| **tryb1** | 5F03-5TA | 148 | 52 |
| **tysy** | 1TRG-CB3 | 109 | 62 |
| **urok** | 4FU7-1UP | 162 | 61 |
| **vgfr2** | 2XIR-00J | 409 | 60 |
| **wee1** | 5VD4-99J | 102 | 60 |
| **xiap** | 4HY0-1AQ | 100 | 52 |

**Table S2.** Detailed list of filtered targets (Morgan-based Tanimoto index ≤ 0.1).

| **Target** | **Reference ID**  Target-Ligand | **Number of actives** | **Decoy-to-hit ratio** |
| --- | --- | --- | --- |
| **aces** | 2CMF-F11 | 135 | 194 |
| **ada17** | 3EDZ-INN | 377 | 95 |
| **akt2** | 2JDO-I5S | 42 | 164 |
| **andr** | 2AXA-FHM | 123 | 117 |
| **casp3** | 4QU9-ACE | 82 | 130 |
| **gcr** | 3E7C-866 | 123 | 122 |
| **hivrt** | 6ELI-T27 | 209 | 90 |
| **hs90a** | 1UY9-PU6 | 33 | 147 |
| **mp2k1** | 3DV3-MEK1 | 82 | 99 |
| **nos1** | 1QWC-H4B | 75 | 107 |
| **pa2ga** | 1AYP-INB | 62 | 83 |
| **pgh1** | 2OYE-IM8 | 96 | 113 |
| **pgh2** | 5W58-FF81 | 120 | 193 |
| **prgr** | 2OVM-AS0 | 189 | 83 |
| **tgfr1** | 2X7O-ZOP | 59 | 144 |

**Table S3.** Statistical summary of VS metrics for the different conformational queries. The AUC and ROCe values (mean ± standard deviation) correspond to the average determined for the set of targets included in the DUD-E^+^ dataset (see individual values in Tables S4-S9). For Q_ENS_, the results are provided using the Parallel algorithm.

| Query | **AUC** | **ROCe 1%** | **ROCe 2%** | **ROCe 5%** |
| --- | --- | --- | --- | --- |
| Q_XR_ | 0.64 ± 0.15 | 13.0 ± 14.2 | 8.1 ± 8.1 | 4.7 ± 3.7 |
| Q_EMXR_ | 0.63 ± 0.15 | 12.9 ± 14.2 | 8.0 ± 7.9 | 4.6 ± 3.7 |
| Q_LEG_ | 0.62 ± 0.15 | 12.5 ± 15.3 | 7.7 ± 8.4 | 4.4 ± 3.8 |
| Q_LEW_ | 0.64 ± 0.14 | 15.3 ± 17.2 | 8.0 ± 7.9 | 4.7 ± 3.8 |
| Q_ENS_ | 0.65 ± 0.14 | 14.7 ± 16.5 | 9.0 ± 8.9 | 4.9 ± 4.0 |

**Table S4.** AUC values obtained for the different queries and the hydrophobicity (Hyphar)-based descriptors for the targets in the DUD-E^+^ dataset.

| **Target** | **Reference ID**  Target-Ligand | ***Q_XR_*** | ***Q_EMRX_*** | ***Q_LEG_*** | ***Q_LEW_*** | ***Q_ENS_*** |
| --- | --- | --- | --- | --- | --- | --- |
| **aa2ar** | 5K2D-ZMA | 0.61 | 0.63 | 0.6 | 0.62 | 0.64 |
| **abl1** | 2F4J-VX6 | 0.67 | 0.59 | 0.34 | 0.38 | 0.47 |
| **ace** | 6F9U-D0W | 0.69 | 0.73 | 0.72 | 0.67 | 0.73 |
| **aces** | 2CMF-F11 | 0.69 | 0.72 | 0.8 | 0.73 | 0.76 |
| **ada** | 1WXY-FRK | 0.59 | 0.57 | 0.65 | 0.50 | 0.52 |
| **ada17** | 3EDZ-INN | 0.66 | 0.54 | 0.52 | 0.58 | 0.55 |
| **adrb1** | 4AMJ-CVD | 0.65 | 0.59 | 0.56 | 0.68 | 0.64 |
| **adrb2** | 3P0G-P0G | 0.6 | 0.61 | 0.57 | 0.62 | 0.61 |
| **akt1** | 3QKL-SMR | 0.52 | 0.42 | 0.51 | 0.57 | 0.55 |
| **akt2** | 2JDO-I5S | 0.35 | 0.32 | 0.44 | 0.50 | 0.47 |
| **aldr** | 4LAZ-1WW | 0.57 | 0.58 | 0.59 | 0.59 | 0.58 |
| **andr** | 2AXA-FHM | 0.59 | 0.56 | 0.55 | 0.58 | 0.63 |
| **aofb** | 2V5Z-SAG | 0.71 | 0.66 | 0.66 | 0.68 | 0.68 |
| **bace1** | 2QMD-CS7 | 0.70 | 0.70 | 0.70 | 0.64 | 0.75 |
| **braf** | 3OG7-032 | 0.50 | 0.42 | 0.36 | 0.34 | 0.46 |
| **cah2** | 1BN4-AL9 | 0.66 | 0.65 | 0.59 | 0.65 | 0.68 |
| **casp3** | 4QU9-ACE | 0.66 | 0.68 | 0.69 | 0.69 | 0.71 |
| **cdk2** | 1OGU-ST8 | 0.54 | 0.58 | 0.65 | 0.59 | 0.69 |
| **comt** | 4XUD-43H | 0.63 | 0.64 | 0.62 | 0.62 | 0.63 |
| **csf1r** | 3DPK-8C5 | 0.70 | 0.75 | 0.68 | 0.68 | 0.73 |
| **def** | 1ICJ-2PE | 0.69 | 0.63 | 0.65 | 0.34 | 0.39 |
| **dhi1** | 3FRJ-A491 | 0.69 | 0.67 | 0.75 | 0.74 | 0.72 |
| **dpp4** | 2HHA-3TP | 0.60 | 0.58 | 0.57 | 0.61 | 0.61 |
| **dyr** | 3FS6-DH1 | 0.58 | 0.65 | 0.46 | 0.65 | 0.65 |
| **egfr** | 3BEL-POX | 0.62 | 0.66 | 0.55 | 0.64 | 0.64 |
| **esr1** | 2R6Y-LLC1 | 0.68 | 0.67 | 0.67 | 0.67 | 0.68 |
| **esr2** | 2FSZ-OHT | 0.70 | 0.70 | 0.70 | 0.70 | 0.71 |
| **fa10** | 2XBX-RR8 | 0.52 | 0.56 | 0.51 | 0.51 | 0.55 |
| **fa7** | 2F9B-N1H | 0.75 | 0.78 | 0.74 | 0.61 | 0.80 |
| **fabp4** | 5HZ8-65Z | 0.65 | 0.64 | 0.64 | 0.65 | 0.63 |
| **fak1** | 4GU6-10N1 | 0.41 | 0.46 | 0.70 | 0.51 | 0.58 |
| **fgfr1** | 4RWJ-66T | 0.70 | 0.70 | 0.70 | 0.66 | 0.70 |
| **fkb1a** | 1FKH-SBX | 0.79 | 0.8 | 0.79 | 0.74 | 0.82 |
| **fnta** | 2IEJ-S48 | 0.68 | 0.68 | 0.63 | 0.62 | 0.65 |
| **fpps** | 1YV5-RIS1 | 0.87 | 0.88 | 0.89 | 0.88 | 0.89 |
| **gcr** | 3E7C-866 | 0.53 | 0.52 | 0.58 | 0.49 | 0.54 |
| **glcm** | 2V3E-NND | 0.62 | 0.68 | 0.64 | 0.69 | 0.62 |
| **gria2** | 3H03-UBP1 | 0.51 | 0.47 | 0.49 | 0.48 | 0.52 |
| **grik1** | 1VSO-AT11 | 0.58 | 0.61 | 0.63 | 0.64 | 0.64 |
| **hdac2** | 4LY1-20Y | 0.62 | 0.58 | 0.66 | 0.58 | 0.59 |
| **hdac8** | 3SFF-0DI | 0.48 | 0.49 | 0.51 | 0.51 | 0.45 |
| **hivint** | 5KGW-7SK | 0.41 | 0.42 | 0.42 | 0.48 | 0.41 |
| **hivpr** | 1EBZ-BEC | 0.78 | 0.77 | 0.82 | 0.80 | 0.81 |
| **hivrt** | 6ELI-T27 | 0.67 | 0.65 | 0.42 | 0.40 | 0.55 |
| **hmdh** | 2R4F-RIE1 | 0.87 | 0.87 | 0.86 | 0.88 | 0.89 |
| **hs90a** | 1UY9-PU6 | 0.77 | 0.76 | 0.73 | 0.75 | 0.74 |
| **hxk4** | 4IXC-1JD | 0.89 | 0.83 | 0.84 | 0.87 | 0.80 |
| **inha** | 5CPF-53K2 | 0.77 | 0.78 | 0.81 | 0.78 | 0.80 |
| **jak2** | 5WIM-35R | 0.58 | 0.57 | 0.60 | 0.63 | 0.65 |
| **kif11** | 4A5Y-G7X | 0.61 | 0.62 | 0.63 | 0.61 | 0.70 |
| **lck** | 2OF2-547 | 0.29 | 0.27 | 0.26 | 0.52 | 0.26 |
| **lkha4** | 3FTX-BES | 0.37 | 0.33 | 0.43 | 0.47 | 0.37 |
| **mapk2** | 2PZY-B18 | 0.81 | 0.78 | 0.75 | 0.77 | 0.79 |
| **mcr** | 5L7G-6QE | 0.76 | 0.69 | 0.60 | 0.48 | 0.54 |
| **met** | 5HTI-66L | 0.77 | 0.76 | 0.76 | 0.75 | 0.76 |
| **mk01** | 3I5Z-Z48 | 0.77 | 0.76 | 0.82 | 0.74 | 0.84 |
| **mk10** | 4Y5H-519 | 0.54 | 0.5 | 0.53 | 0.51 | 0.55 |
| **mk14** | 3D83-GK6 | 0.62 | 0.62 | 0.62 | 0.66 | 0.65 |
| **mmp13** | 3ELM-24F | 0.72 | 0.73 | 0.74 | 0.70 | 0.71 |
| **mp2k1** | 3DV3-MEK1 | 0.51 | 0.55 | 0.54 | 0.48 | 0.56 |
| **nos1** | 1QWC-H4B | 0.46 | 0.47 | 0.44 | 0.46 | 0.44 |
| **nram** | 1B9V-RA2 | 0.88 | 0.89 | 0.87 | 0.88 | 0.86 |
| **pa2ga** | 1AYP-INB | 0.78 | 0.74 | 0.62 | 0.63 | 0.70 |
| **parp1** | 4R6E-3JD | 0.74 | 0.74 | 0.72 | 0.70 | 0.74 |
| **pde5a** | 3TGE-TGE | 0.48 | 0.5 | 0.56 | 0.61 | 0.68 |
| **pgh1** | 2OYE-IM8 | 0.48 | 0.48 | 0.46 | 0.54 | 0.51 |
| **pgh2** | 5W58-FF81 | 0.50 | 0.53 | 0.54 | 0.41 | 0.51 |
| **plk1** | 4A4L-939 | 0.51 | 0.51 | 0.73 | 0.67 | 0.69 |
| **pnph** | 4EAR-IM52 | 0.69 | 0.68 | 0.68 | 0.71 | 0.70 |
| **ppara** | 1I7G-AZ2 | 0.82 | 0.85 | 0.32 | 0.86 | 0.88 |
| **ppard** | 5U3Z-7UA | 0.86 | 0.62 | 0.83 | 0.87 | 0.89 |
| **pparg** | 5YCP-BRL | 0.48 | 0.33 | 0.28 | 0.69 | 0.38 |
| **prgr** | 2OVM-AS0 | 0.76 | 0.80 | 0.77 | 0.75 | 0.78 |
| **ptn1** | 1NL9-989 | 0.66 | 0.67 | 0.65 | 0.73 | 0.58 |
| **pur2** | 4EW3-DXZ | 0.97 | 0.94 | 0.89 | 0.96 | 0.95 |
| **pygm** | 1WV0-BN41 | 0.40 | 0.41 | 0.43 | 0.36 | 0.44 |
| **pyrd** | 3KVJ-1X5 | 0.67 | 0.71 | 0.69 | 0.75 | 0.6 |
| **reni** | 2V16-C472 | 0.65 | 0.63 | 0.6 | 0.59 | 0.6 |
| **rock1** | 5KKS-6U1 | 0.52 | 0.52 | 0.54 | 0.52 | 0.53 |
| **rxra** | 4RMD-3SW | 0.94 | 0.92 | 0.92 | 0.89 | 0.92 |
| **sahh** | 3NJ4-AFX3 | 0.82 | 0.84 | 0.87 | 0.83 | 0.88 |
| **src** | 2OIQ-STI | 0.74 | 0.74 | 0.70 | 0.73 | 0.74 |
| **tgfr1** | 2X7O-ZOP | 0.48 | 0.48 | 0.49 | 0.46 | 0.47 |
| **thb** | 1Q4X-G24 | 0.86 | 0.86 | 0.84 | 0.86 | 0.88 |
| **thrb** | 2C8Y-C3M | 0.56 | 0.55 | 0.43 | 0.46 | 0.45 |
| **try1** | 1O3D-780 | 0.5 | 0.51 | 0.5 | 0.51 | 0.52 |
| **tryb1** | 5F03-5TA | 0.53 | 0.29 | 0.28 | 0.62 | 0.49 |
| **tysy** | 1TRG-CB3 | 0.71 | 0.71 | 0.59 | 0.71 | 0.77 |
| **urok** | 4FU7-1UP | 0.53 | 0.43 | 0.43 | 0.48 | 0.47 |
| **vgfr2** | 2XIR-00J | 0.77 | 0.77 | 0.72 | 0.75 | 0.75 |
| **wee1** | 5VD4-99J | 0.66 | 0.66 | 0.47 | 0.64 | 0.47 |
| **xiap** | 4HY0-1AQ | 0.83 | 0.84 | 0.79 | 0.83 | 0.87 |

**Table S5.** ROCe 1% values obtained for the different queries and the hydrophobicity (Hyphar)-based descriptors for the targets in the DUD-E^+^ dataset.

| **Target** | **Reference ID**  Target-Ligand | ***Q_XR_*** | ***Q_EMRX_*** | ***Q_LEG_*** | ***Q_LEW_*** | ***Q_ENS_*** |
| --- | --- | --- | --- | --- | --- | --- |
| **aa2ar** | 5K2D-ZMA | 10.4 | 11.6 | 16.8 | 10.8 | 16.8 |
| **abl1** | 2F4J-VX6 | 3.3 | 2.8 | 0.6 | 0.6 | 1.7 |
| **ace** | 6F9U-D0W | 8.5 | 5.0 | 6.0 | 7.8 | 9.2 |
| **aces** | 2CMF-F11 | 19.0 | 21.0 | 27.6 | 20.5 | 27.4 |
| **ada** | 1WXY-FRK | 0.0 | 0.0 | 0.0 | 0.0 | 1.1 |
| **ada17** | 3EDZ-INN | 2.3 | 2.6 | 2.6 | 3.0 | 4.5 |
| **adrb1** | 4AMJ-CVD | 13.4 | 11.3 | 8.1 | 15.8 | 15.0 |
| **adrb2** | 3P0G-P0G | 15.6 | 13.9 | 10.8 | 17.8 | 15.2 |
| **akt1** | 3QKL-SMR | 0.0 | 0.0 | 0.0 | 0.3 | 1.0 |
| **akt2** | 2JDO-I5S | 3.4 | 3.4 | 1.7 | 1.7 | 2.6 |
| **aldr** | 4LAZ-1WW | 12.0 | 12.0 | 15.7 | 16.4 | 12.0 |
| **andr** | 2AXA-FHM | 1.1 | 3.4 | 3.0 | 2.6 | 8.2 |
| **aofb** | 2V5Z-SAG | 16.4 | 14.8 | 14.8 | 14.8 | 15.6 |
| **bace1** | 2QMD-CS7 | 18.0 | 18.7 | 20.1 | 12.4 | 31.5 |
| **braf** | 3OG7-032 | 0.0 | 0.0 | 0.0 | 0.0 | 0.0 |
| **cah2** | 1BN4-AL9 | 19.1 | 15.5 | 8.9 | 9.2 | 15.9 |
| **casp3** | 4QU9-ACE | 5.5 | 13.6 | 8.5 | 9.6 | 8.0 |
| **cdk2** | 1OGU-ST8 | 4.6 | 7.0 | 5.5 | 4.2 | 7.4 |
| **comt** | 4XUD-43H | 2.4 | 2.4 | 2.4 | 2.4 | 7.3 |
| **csf1r** | 3DPK-8C5 | 24.7 | 29.5 | 22.9 | 24.1 | 28.9 |
| **def** | 1ICJ-2PE | 2.0 | 2.0 | 9.8 | 0.0 | 1.0 |
| **dhi1** | 3FRJ-A491 | 10.9 | 8.2 | 19.1 | 15.2 | 15.2 |
| **dpp4** | 2HHA-3TP | 3.2 | 1.5 | 1.9 | 5.1 | 3.9 |
| **dyr** | 3FS6-DH1 | 5.2 | 6.9 | 3.5 | 7.4 | 8.2 |
| **egfr** | 3BEL-POX | 8.7 | 11.6 | 8.5 | 7.6 | 13.3 |
| **esr1** | 2R6Y-LLC1 | 45.2 | 45.4 | 38.1 | 46.5 | 44.4 |
| **esr2** | 2FSZ-OHT | 44.7 | 42.5 | 42.2 | 44.4 | 42.8 |
| **fa10** | 2XBX-RR8 | 3.7 | 7.1 | 4.1 | 1.9 | 6.5 |
| **fa7** | 2F9B-N1H | 5.3 | 2.6 | 6.1 | 6.1 | 5.3 |
| **fabp4** | 5HZ8-65Z | 0.0 | 0.0 | 0.0 | 0.0 | 0.0 |
| **fak1** | 4GU6-10N1 | 1.0 | 8.0 | 5.0 | 3.0 | 5.0 |
| **fgfr1** | 4RWJ-66T | 21.6 | 20.1 | 18.7 | 13.7 | 21.6 |
| **fkb1a** | 1FKH-SBX | 31.5 | 29.7 | 28.8 | 32.4 | 44.1 |
| **fnta** | 2IEJ-S48 | 6.9 | 6.6 | 4.4 | 2.0 | 5.7 |
| **fpps** | 1YV5-RIS1 | 45.9 | 42.4 | 49.4 | 49.4 | 50.6 |
| **gcr** | 3E7C-866 | 3.5 | 3.5 | 4.7 | 3.9 | 4.3 |
| **glcm** | 2V3E-NND | 14.8 | 20.4 | 16.7 | 16.7 | 16.7 |
| **gria2** | 3H03-UBP1 | 1.9 | 0.0 | 0.0 | 1.9 | 1.3 |
| **grik1** | 1VSO-AT11 | 0.0 | 3.0 | 5.0 | 5.9 | 7.9 |
| **hdac2** | 4LY1-20Y | 4.9 | 6.0 | 4.3 | 2.2 | 2.2 |
| **hdac8** | 3SFF-0DI | 0.0 | 0.6 | 1.2 | 1.2 | 0.0 |
| **hivint** | 5KGW-7SK | 0.0 | 0.0 | 0.0 | 0.0 | 0.0 |
| **hivpr** | 1EBZ-BEC | 28.7 | 26.7 | 17.9 | 21.1 | 32.1 |
| **hivrt** | 6ELI-T27 | 4.4 | 0.9 | 0.0 | 0.3 | 0.3 |
| **hmdh** | 2R4F-RIE1 | 46.5 | 51.8 | 49.4 | 58.2 | 61.2 |
| **hs90a** | 1UY9-PU6 | 29.6 | 29.6 | 27.3 | 33.0 | 33.0 |
| **hxk4** | 4IXC-1JD | 35.9 | 15.2 | 3.3 | 21.7 | 6.5 |
| **inha** | 5CPF-53K2 | 9.3 | 9.3 | 16.3 | 11.6 | 2.3 |
| **jak2** | 5WIM-35R | 0.9 | 1.9 | 5.6 | 6.5 | 5.6 |
| **kif11** | 4A5Y-G7X | 6.9 | 6.9 | 6.0 | 2.6 | 7.8 |
| **lck** | 2OF2-547 | 1.2 | 0.7 | 0.5 | 5.0 | 1.0 |
| **lkha4** | 3FTX-BES | 0.6 | 0.0 | 0.0 | 1.2 | 0.0 |
| **mapk2** | 2PZY-B18 | 22.8 | 23.8 | 21.8 | 12.9 | 23.8 |
| **mcr** | 5L7G-6QE | 3.2 | 3.2 | 3.2 | 0.0 | 2.1 |
| **met** | 5HTI-66L | 20.5 | 20.5 | 19.3 | 16.3 | 19.9 |
| **mk01** | 3I5Z-Z48 | 22.8 | 26.6 | 40.5 | 27.9 | 36.7 |
| **mk10** | 4Y5H-519 | 5.8 | 4.8 | 4.8 | 0.0 | 4.8 |
| **mk14** | 3D83-GK6 | 10.0 | 7.6 | 14.9 | 8.7 | 11.8 |
| **mmp13** | 3ELM-24F | 15.7 | 15.4 | 18.7 | 15.2 | 18.9 |
| **mp2k1** | 3DV3-MEK1 | 2.5 | 3.3 | 5.8 | 1.7 | 3.3 |
| **nos1** | 1QWC-H4B | 0.0 | 0.0 | 0.0 | 1.0 | 0.0 |
| **nram** | 1B9V-RA2 | 14.3 | 19.4 | 14.3 | 19.4 | 20.4 |
| **pa2ga** | 1AYP-INB | 10.1 | 10.1 | 7.1 | 1.0 | 3.0 |
| **parp1** | 4R6E-3JD | 21.5 | 23.6 | 19.5 | 15.8 | 24.2 |
| **pde5a** | 3TGE-TGE | 10.3 | 10.8 | 5.3 | 12.1 | 14.6 |
| **pgh1** | 2OYE-IM8 | 1.0 | 0.5 | 1.0 | 1.5 | 1.0 |
| **pgh2** | 5W58-FF81 | 4.1 | 5.8 | 3.2 | 0.0 | 0.9 |
| **plk1** | 4A4L-939 | 1.9 | 2.8 | 9.4 | 6.5 | 8.4 |
| **pnph** | 4EAR-IM52 | 16.5 | 16.5 | 23.3 | 20.4 | 24.3 |
| **ppara** | 1I7G-AZ2 | 23.3 | 27.6 | 1.1 | 27.9 | 30.8 |
| **ppard** | 5U3Z-7UA | 15.8 | 4.6 | 11.7 | 30.0 | 22.5 |
| **pparg** | 5YCP-BRL | 1.5 | 0.6 | 0.8 | 4.3 | 1.0 |
| **prgr** | 2OVM-AS0 | 18.4 | 23.9 | 22.5 | 18.4 | 25.6 |
| **ptn1** | 1NL9-989 | 8.5 | 9.2 | 4.6 | 10.0 | 6.9 |
| **pur2** | 4EW3-DXZ | 58.0 | 48.0 | 76.0 | 60.0 | 80.0 |
| **pygm** | 1WV0-BN41 | 0.0 | 0.0 | 0.0 | 0.0 | 0.0 |
| **pyrd** | 3KVJ-1X5 | 0.9 | 3.6 | 8.1 | 9.0 | 7.2 |
| **reni** | 2V16-C472 | 13.5 | 9.6 | 12.5 | 3.9 | 14.4 |
| **rock1** | 5KKS-6U1 | 0.0 | 1.0 | 4.0 | 2.0 | 1.0 |
| **rxra** | 4RMD-3SW | 67.9 | 67.9 | 71.0 | 47.3 | 63.4 |
| **sahh** | 3NJ4-AFX3 | 25.4 | 39.7 | 58.7 | 36.5 | 66.7 |
| **src** | 2OIQ-STI | 27.3 | 26.9 | 12.2 | 18.7 | 19.9 |
| **tgfr1** | 2X7O-ZOP | 0.8 | 0.8 | 0.8 | 0.8 | 0.8 |
| **thb** | 1Q4X-G24 | 29.1 | 26.2 | 40.8 | 36.9 | 41.8 |
| **thrb** | 2C8Y-C3M | 0.4 | 0.0 | 0.0 | 0.0 | 0.4 |
| **try1** | 1O3D-780 | 1.8 | 2.2 | 1.8 | 2.2 | 2.2 |
| **tryb1** | 5F03-5TA | 4.1 | 1.4 | 2.0 | 4.1 | 2.7 |
| **tysy** | 1TRG-CB3 | 41.3 | 39.5 | 24.8 | 40.4 | 48.6 |
| **urok** | 4FU7-1UP | 4.3 | 3.7 | 3.7 | 3.1 | 4.3 |
| **vgfr2** | 2XIR-00J | 10.3 | 11.3 | 4.7 | 8.3 | 8.1 |
| **wee1** | 5VD4-99J | 8.8 | 8.8 | 2.0 | 4.9 | 3.9 |
| **xiap** | 4HY0-1AQ | 25.0 | 26.0 | 7.0 | 16.0 | 12.0 |

**Table S6.** ROCe 2% values obtained for the different queries and the hydrophobicity (Hyphar)-based descriptors for the targets in the DUD-E^+^ dataset.

| **Target** | **Reference ID**  Target-Ligand | ***Q_XR_*** | ***Q_EMRX_*** | ***Q_LEG_*** | ***Q_LEW_*** | ***Q_ENS_*** |
| --- | --- | --- | --- | --- | --- | --- |
| **aa2ar** | 5K2D-ZMA | 6.5 | 6.6 | 9.4 | 6.5 | 10.5 |
| **abl1** | 2F4J-VX6 | 4.1 | 2.2 | 0.6 | 0.6 | 0.8 |
| **ace** | 6F9U-D0W | 6.7 | 6.0 | 3.9 | 5.9 | 7.3 |
| **aces** | 2CMF-F11 | 11.6 | 12.1 | 16.1 | 12.9 | 15.1 |
| **ada** | 1WXY-FRK | 0.5 | 0.5 | 0.5 | 0.0 | 0.5 |
| **ada17** | 3EDZ-INN | 2.3 | 2.4 | 2.4 | 2.1 | 2.8 |
| **adrb1** | 4AMJ-CVD | 9.1 | 8.1 | 5.7 | 11.1 | 10.5 |
| **adrb2** | 3P0G-P0G | 8.9 | 8.9 | 6.9 | 9.3 | 9.1 |
| **akt1** | 3QKL-SMR | 0.2 | 0.0 | 0.0 | 1.2 | 1.2 |
| **akt2** | 2JDO-I5S | 1.7 | 2.1 | 1.3 | 1.3 | 2.6 |
| **aldr** | 4LAZ-1WW | 8.5 | 8.2 | 9.1 | 8.8 | 7.9 |
| **andr** | 2AXA-FHM | 1.5 | 2.0 | 1.9 | 2.2 | 5.4 |
| **aofb** | 2V5Z-SAG | 11.1 | 8.2 | 8.6 | 9.0 | 9.8 |
| **bace1** | 2QMD-CS7 | 11.5 | 11.5 | 12.4 | 7.6 | 18.9 |
| **braf** | 3OG7-032 | 0.3 | 0.0 | 0.0 | 0.7 | 0.3 |
| **cah2** | 1BN4-AL9 | 12.0 | 10.5 | 5.4 | 6.6 | 10.4 |
| **casp3** | 4QU9-ACE | 5.0 | 10.1 | 6.0 | 9.6 | 7.0 |
| **cdk2** | 1OGU-ST8 | 4.3 | 4.9 | 4.3 | 3.6 | 5.5 |
| **comt** | 4XUD-43H | 3.7 | 2.4 | 1.2 | 2.4 | 3.7 |
| **csf1r** | 3DPK-8C5 | 13.0 | 16.3 | 13.6 | 12.7 | 15.7 |
| **def** | 1ICJ-2PE | 4.9 | 2.9 | 6.4 | 0.0 | 0.5 |
| **dhi1** | 3FRJ-A491 | 7.9 | 5.9 | 11.7 | 10.8 | 9.1 |
| **dpp4** | 2HHA-3TP | 2.6 | 1.2 | 1.4 | 4.3 | 2.6 |
| **dyr** | 3FS6-DH1 | 3.9 | 5.0 | 2.4 | 5.6 | 5.6 |
| **egfr** | 3BEL-POX | 6.8 | 7.6 | 4.8 | 5.2 | 8.5 |
| **esr1** | 2R6Y-LLC1 | 23.1 | 23.8 | 20.8 | 24.4 | 23.2 |
| **esr2** | 2FSZ-OHT | 23.0 | 22.3 | 22.8 | 23.3 | 22.6 |
| **fa10** | 2XBX-RR8 | 3.5 | 4.8 | 2.6 | 1.7 | 4.3 |
| **fa7** | 2F9B-N1H | 3.9 | 4.4 | 3.9 | 4 | 3.5 |
| **fabp4** | 5HZ8-65Z | 0.0 | 0.0 | 0.0 | 0.0 | 0.0 |
| **fak1** | 4GU6-10N1 | 1.0 | 4.5 | 4.5 | 2.0 | 3.0 |
| **fgfr1** | 4RWJ-66T | 13.7 | 12.2 | 10.4 | 9.4 | 12.6 |
| **fkb1a** | 1FKH-SBX | 17.6 | 16.2 | 16.2 | 19.4 | 23.4 |
| **fnta** | 2IEJ-S48 | 5.7 | 5.0 | 3.2 | 1.7 | 3.7 |
| **fpps** | 1YV5-RIS1 | 23.5 | 23.5 | 27.7 | 28.2 | 28.2 |
| **gcr** | 3E7C-866 | 2.5 | 1.7 | 2.5 | 2.3 | 2.1 |
| **glcm** | 2V3E-NND | 7.4 | 10.2 | 8.3 | 9.3 | 11.1 |
| **gria2** | 3H03-UBP1 | 1.9 | 0.3 | 0.6 | 1.3 | 1.3 |
| **grik1** | 1VSO-AT11 | 1.0 | 5.0 | 5.0 | 4.5 | 5.0 |
| **hdac2** | 4LY1-20Y | 3.0 | 4.9 | 3.8 | 2.7 | 1.6 |
| **hdac8** | 3SFF-0DI | 0.3 | 0.6 | 0.9 | 0.9 | 0.6 |
| **hivint** | 5KGW-7SK | 0.0 | 0.0 | 0.0 | 0.5 | 0.5 |
| **hivpr** | 1EBZ-BEC | 17.1 | 16.5 | 13.3 | 15.5 | 21.0 |
| **hivrt** | 6ELI-T27 | 3.4 | 1.2 | 0.4 | 0.6 | 0.9 |
| **hmdh** | 2R4F-RIE1 | 27.4 | 30.9 | 27.4 | 30.9 | 34.7 |
| **hs90a** | 1UY9-PU6 | 15.9 | 15.9 | 15.3 | 17.1 | 17.1 |
| **hxk4** | 4IXC-1JD | 18.5 | 12.5 | 2.7 | 14.7 | 7.1 |
| **inha** | 5CPF-53K2 | 8.1 | 7.0 | 10.5 | 10.5 | 4.7 |
| **jak2** | 5WIM-35R | 0.9 | 0.9 | 4.7 | 3.3 | 5.6 |
| **kif11** | 4A5Y-G7X | 3.5 | 3.5 | 3.0 | 3.9 | 5.6 |
| **lck** | 2OF2-547 | 0.7 | 0.6 | 0.6 | 3.9 | 0.8 |
| **lkha4** | 3FTX-BES | 0.6 | 0.0 | 0.0 | 0.9 | 0.0 |
| **mapk2** | 2PZY-B18 | 13.4 | 14.4 | 11.9 | 11.4 | 13.9 |
| **mcr** | 5L7G-6QE | 3.7 | 2.1 | 1.6 | 0.0 | 1.6 |
| **met** | 5HTI-66L | 12.4 | 12.1 | 13.6 | 12.7 | 13.3 |
| **mk01** | 3I5Z-Z48 | 13.3 | 15.2 | 24.7 | 15.2 | 20.9 |
| **mk10** | 4Y5H-519 | 2.9 | 2.9 | 2.4 | 1.4 | 2.9 |
| **mk14** | 3D83-GK6 | 7.1 | 7.2 | 8.7 | 6.4 | 7.8 |
| **mmp13** | 3ELM-24F | 10.1 | 8.7 | 11.1 | 8.8 | 10.8 |
| **mp2k1** | 3DV3-MEK1 | 2.9 | 2.5 | 2.9 | 1.2 | 2.5 |
| **nos1** | 1QWC-H4B | 0.0 | 0.5 | 0.0 | 1.0 | 0.0 |
| **nram** | 1B9V-RA2 | 11.7 | 16.3 | 13.3 | 14.8 | 13.3 |
| **pa2ga** | 1AYP-INB | 12.1 | 8.1 | 5.6 | 2.5 | 3.5 |
| **parp1** | 4R6E-3JD | 12.9 | 13.4 | 12.5 | 9.4 | 13.9 |
| **pde5a** | 3TGE-TGE | 5.8 | 6.0 | 3.3 | 7.0 | 8.2 |
| **pgh1** | 2OYE-IM8 | 1.0 | 0.8 | 0.8 | 0.8 | 0.5 |
| **pgh2** | 5W58-FF81 | 3.1 | 3.9 | 2.6 | 0.5 | 1.4 |
| **plk1** | 4A4L-939 | 0.9 | 1.4 | 7.5 | 5.6 | 6.1 |
| **pnph** | 4EAR-IM52 | 9.7 | 10.2 | 13.1 | 11.2 | 13.1 |
| **ppara** | 1I7G-AZ2 | 16.0 | 18.5 | 1.1 | 19.7 | 20.2 |
| **ppard** | 5U3Z-7UA | 11.7 | 4.6 | 9.2 | 21.3 | 17.5 |
| **pparg** | 5YCP-BRL | 1.0 | 0.4 | 0.4 | 3.9 | 0.6 |
| **prgr** | 2OVM-AS0 | 11.4 | 13.7 | 14.2 | 12.6 | 15.0 |
| **ptn1** | 1NL9-989 | 5.8 | 7.3 | 5.0 | 6.9 | 5.4 |
| **pur2** | 4EW3-DXZ | 34.0 | 30.0 | 40.0 | 33.0 | 41.0 |
| **pygm** | 1WV0-BN41 | 0.0 | 0.0 | 0.0 | 0.0 | 0.0 |
| **pyrd** | 3KVJ-1X5 | 0.5 | 3.2 | 6.3 | 8.6 | 5.4 |
| **reni** | 2V16-C472 | 7.7 | 5.8 | 6.7 | 1.9 | 7.7 |
| **rock1** | 5KKS-6U1 | 0.0 | 0.5 | 3.5 | 1.5 | 2.5 |
| **rxra** | 4RMD-3SW | 38.2 | 35.9 | 37.0 | 30.2 | 35.1 |
| **sahh** | 3NJ4-AFX3 | 15.1 | 23.0 | 32.5 | 20.6 | 35.7 |
| **src** | 2OIQ-STI | 17.1 | 16.0 | 10.5 | 13.0 | 15.0 |
| **tgfr1** | 2X7O-ZOP | 1.1 | 1.5 | 1.1 | 1.5 | 1.9 |
| **thb** | 1Q4X-G24 | 18.9 | 19.4 | 24.3 | 20.9 | 25.2 |
| **thrb** | 2C8Y-C3M | 0.3 | 0.2 | 0.0 | 0.0 | 0.2 |
| **try1** | 1O3D-780 | 1.1 | 1.8 | 1.7 | 1.9 | 2.2 |
| **tryb1** | 5F03-5TA | 2.0 | 1.4 | 1.0 | 3.0 | 2.4 |
| **tysy** | 1TRG-CB3 | 22.0 | 21.1 | 12.8 | 22.0 | 26.2 |
| **urok** | 4FU7-1UP | 3.1 | 2.5 | 2.2 | 2.2 | 2.8 |
| **vgfr2** | 2XIR-00J | 7.5 | 7.7 | 4.0 | 6.4 | 6.0 |
| **wee1** | 5VD4-99J | 4.9 | 6.9 | 2.5 | 4.9 | 3.9 |
| **xiap** | 4HY0-1AQ | 16.5 | 16.0 | 10.0 | 11.0 | 15.0 |

**Table S7.** ROCe 5% values obtained for the different queries and the hydrophobicity (Hyphar)-based descriptors for the targets in the DUD-E^+^ dataset.

| **Target** | **Reference ID**  Target-Ligand | ***Q_XR_*** | ***Q_EMRX_*** | ***Q_LEG_*** | ***Q_LEW_*** | ***Q_ENS_*** |
| --- | --- | --- | --- | --- | --- | --- |
| **aa2ar** | 5K2D-ZMA | 3.4 | 4.1 | 4.9 | 3.8 | 5.1 |
| **abl1** | 2F4J-VX6 | 3.1 | 1.8 | 0.3 | 0.4 | 1.1 |
| **ace** | 6F9U-D0W | 4.0 | 5.0 | 3.8 | 3.8 | 5.3 |
| **aces** | 2CMF-F11 | 6.3 | 6.9 | 8.5 | 6.9 | 7.7 |
| **ada** | 1WXY-FRK | 0.4 | 1.3 | 1.7 | 0.6 | 0.4 |
| **ada17** | 3EDZ-INN | 2.0 | 2.0 | 2.0 | 1.7 | 2.0 |
| **adrb1** | 4AMJ-CVD | 5.2 | 4.4 | 3.6 | 6.7 | 6.2 |
| **adrb2** | 3P0G-P0G | 4.8 | 4.9 | 3.6 | 5.0 | 5.0 |
| **akt1** | 3QKL-SMR | 0.6 | 0.2 | 0.2 | 1.4 | 1.1 |
| **akt2** | 2JDO-I5S | 0.9 | 1.4 | 0.7 | 0.9 | 1.9 |
| **aldr** | 4LAZ-1WW | 3.9 | 4.2 | 4.3 | 4.3 | 3.7 |
| **andr** | 2AXA-FHM | 1.3 | 1.3 | 1.3 | 1.5 | 3.1 |
| **aofb** | 2V5Z-SAG | 6.2 | 4.8 | 5.1 | 4.8 | 5.3 |
| **bace1** | 2QMD-CS7 | 6.6 | 6.8 | 7.2 | 4.2 | 9.3 |
| **braf** | 3OG7-032 | 0.5 | 0.4 | 0.1 | 0.3 | 0.3 |
| **cah2** | 1BN4-AL9 | 6.3 | 5.9 | 3.6 | 4.7 | 5.7 |
| **casp3** | 4QU9-ACE | 2.8 | 6.2 | 5.1 | 6.2 | 5.1 |
| **cdk2** | 1OGU-ST8 | 3.0 | 3.4 | 2.6 | 2.4 | 4.1 |
| **comt** | 4XUD-43H | 3.9 | 3.9 | 4.9 | 4.9 | 3.4 |
| **csf1r** | 3DPK-8C5 | 6.3 | 8.1 | 7.2 | 5.9 | 8.2 |
| **def** | 1ICJ-2PE | 3.5 | 2.2 | 4.5 | 0.2 | 0.6 |
| **dhi1** | 3FRJ-A491 | 4.9 | 4.2 | 6.6 | 6.5 | 6.4 |
| **dpp4** | 2HHA-3TP | 2.2 | 1.7 | 1.7 | 3.5 | 2.3 |
| **dyr** | 3FS6-DH1 | 2.6 | 3.4 | 2.1 | 3.8 | 3.7 |
| **egfr** | 3BEL-POX | 3.7 | 4.6 | 3.3 | 3.2 | 4.7 |
| **esr1** | 2R6Y-LLC1 | 9.9 | 10.2 | 9.2 | 10.1 | 9.8 |
| **esr2** | 2FSZ-OHT | 10.1 | 9.7 | 9.7 | 9.7 | 9.7 |
| **fa10** | 2XBX-RR8 | 2.4 | 2.5 | 1.9 | 1.5 | 2.5 |
| **fa7** | 2F9B-N1H | 3.2 | 2.1 | 3.3 | 3.3 | 2.6 |
| **fabp4** | 5HZ8-65Z | 0.9 | 0.9 | 1.3 | 1.3 | 1.3 |
| **fak1** | 4GU6-10N1 | 1.0 | 2.2 | 2.8 | 1.6 | 2.4 |
| **fgfr1** | 4RWJ-66T | 6.3 | 6.6 | 6.0 | 5.2 | 6.9 |
| **fkb1a** | 1FKH-SBX | 8.8 | 8.5 | 8.5 | 11.0 | 11.4 |
| **fnta** | 2IEJ-S48 | 3.5 | 3.2 | 2.4 | 1.8 | 2.3 |
| **fpps** | 1YV5-RIS1 | 11.8 | 12.9 | 13.4 | 13.9 | 12.7 |
| **gcr** | 3E7C-866 | 1.3 | 1.2 | 1.1 | 1.1 | 1.4 |
| **glcm** | 2V3E-NND | 4.1 | 5.6 | 5.6 | 5.6 | 5.2 |
| **gria2** | 3H03-UBP1 | 2.4 | 0.6 | 1.0 | 1.5 | 0.9 |
| **grik1** | 1VSO-AT11 | 1.0 | 3.2 | 3.0 | 3.2 | 5.2 |
| **hdac2** | 4LY1-20Y | 2.8 | 2.5 | 3.6 | 2.3 | 1.5 |
| **hdac8** | 3SFF-0DI | 0.8 | 0.7 | 1.2 | 1.4 | 0.6 |
| **hivint** | 5KGW-7SK | 0.2 | 0.8 | 0.8 | 0.8 | 0.4 |
| **hivpr** | 1EBZ-BEC | 9.0 | 8.3 | 9.2 | 8.5 | 10.5 |
| **hivrt** | 6ELI-T27 | 3.2 | 1.8 | 0.2 | 0.6 | 0.7 |
| **hmdh** | 2R4F-RIE1 | 12.8 | 14.4 | 13.3 | 13.5 | 14.9 |
| **hs90a** | 1UY9-PU6 | 8.0 | 7.5 | 7.3 | 8.2 | 8.2 |
| **hxk4** | 4IXC-1JD | 10.2 | 7.6 | 4.1 | 8.7 | 4.1 |
| **inha** | 5CPF-53K2 | 5.6 | 5.1 | 5.1 | 6.5 | 5.1 |
| **jak2** | 5WIM-35R | 2.1 | 1.7 | 3.7 | 2.4 | 3.7 |
| **kif11** | 4A5Y-G7X | 2.1 | 1.7 | 1.4 | 2.4 | 4.0 |
| **lck** | 2OF2-547 | 0.8 | 0.5 | 0.4 | 2.5 | 0.6 |
| **lkha4** | 3FTX-BES | 0.2 | 0.0 | 0.0 | 0.7 | 0.4 |
| **mapk2** | 2PZY-B18 | 7.5 | 7.3 | 6.5 | 7.3 | 7.7 |
| **mcr** | 5L7G-6QE | 5.1 | 2.6 | 1.1 | 0.2 | 0.9 |
| **met** | 5HTI-66L | 8.4 | 7.4 | 8.6 | 7.8 | 8.3 |
| **mk01** | 3I5Z-Z48 | 6.3 | 6.8 | 12.2 | 6.3 | 9.4 |
| **mk10** | 4Y5H-519 | 1.9 | 1.9 | 1.7 | 0.8 | 2.1 |
| **mk14** | 3D83-GK6 | 4.8 | 4.6 | 4.5 | 4.5 | 4.9 |
| **mmp13** | 3ELM-24F | 5.4 | 5.2 | 5.3 | 4.7 | 5.7 |
| **mp2k1** | 3DV3-MEK1 | 3.0 | 1.7 | 1.2 | 1.2 | 1.5 |
| **nos1** | 1QWC-H4B | 0.4 | 0.6 | 0.4 | 0.8 | 0.6 |
| **nram** | 1B9V-RA2 | 9.4 | 11.0 | 9.2 | 10.8 | 7.8 |
| **pa2ga** | 1AYP-INB | 7.3 | 5.5 | 4.0 | 3.4 | 4.2 |
| **parp1** | 4R6E-3JD | 6.8 | 6.8 | 6.6 | 5.2 | 7.3 |
| **pde5a** | 3TGE-TGE | 2.6 | 2.7 | 2.3 | 4.0 | 4.1 |
| **pgh1** | 2OYE-IM8 | 1.2 | 1.1 | 0.5 | 1.0 | 0.6 |
| **pgh2** | 5W58-FF81 | 2.3 | 2.3 | 2.0 | 0.6 | 1.2 |
| **plk1** | 4A4L-939 | 1.1 | 1.1 | 4.7 | 4.3 | 3.7 |
| **pnph** | 4EAR-IM52 | 5.4 | 5.8 | 6.0 | 5.2 | 6.6 |
| **ppara** | 1I7G-AZ2 | 8.9 | 9.9 | 0.7 | 10.2 | 10.3 |
| **ppard** | 5U3Z-7UA | 8.9 | 3.4 | 7.6 | 11.7 | 11.7 |
| **pparg** | 5YCP-BRL | 0.8 | 0.2 | 0.4 | 3.4 | 0.7 |
| **prgr** | 2OVM-AS0 | 6.0 | 7.5 | 7.3 | 6.6 | 7.6 |
| **ptn1** | 1NL9-989 | 4.3 | 5.5 | 3.7 | 4.8 | 2.5 |
| **pur2** | 4EW3-DXZ | 16.4 | 13.6 | 16.4 | 15.6 | 17.6 |
| **pygm** | 1WV0-BN41 | 0.0 | 0.0 | 0.0 | 0.0 | 0.0 |
| **pyrd** | 3KVJ-1X5 | 1.8 | 3.4 | 4.3 | 5.4 | 3.6 |
| **reni** | 2V16-C472 | 4.0 | 3.1 | 3.5 | 3.1 | 3.9 |
| **rock1** | 5KKS-6U1 | 0.8 | 0.8 | 2.0 | 1.0 | 1.8 |
| **rxra** | 4RMD-3SW | 16.5 | 15.6 | 16.0 | 15.3 | 15.4 |
| **sahh** | 3NJ4-AFX3 | 10.2 | 10.5 | 14.6 | 10.5 | 15.2 |
| **src** | 2OIQ-STI | 8.9 | 8.9 | 6.0 | 7.8 | 8.4 |
| **tgfr1** | 2X7O-ZOP | 1.1 | 1.1 | 1.4 | 1.5 | 1.2 |
| **thb** | 1Q4X-G24 | 10.5 | 10.7 | 11.7 | 11.7 | 12.4 |
| **thrb** | 2C8Y-C3M | 0.7 | 0.4 | 0.3 | 0.0 | 0.4 |
| **try1** | 1O3D-780 | 1.0 | 1.3 | 1.6 | 1.4 | 1.6 |
| **tryb1** | 5F03-5TA | 2.2 | 1.1 | 1.2 | 2.6 | 1.4 |
| **tysy** | 1TRG-CB3 | 9.4 | 9.7 | 6.4 | 4.3 | 11.6 |
| **urok** | 4FU7-1UP | 2.0 | 1.1 | 1.4 | 10.5 | 1.6 |
| **vgfr2** | 2XIR-00J | 5.8 | 5.4 | 3.0 | 1.4 | 4.1 |
| **wee1** | 5VD4-99J | 4.3 | 4.5 | 1.8 | 4.8 | 2.0 |
| **xiap** | 4HY0-1AQ | 9.4 | 9.6 | 6.2 | 3.7 | 10.8 |

**Table S8.** AUC and ROCe 1% values of the *Q_ENS_* query using MAX score. parallel selection and rank sum fusion algorithms and the hydrophobicity (Hyphar)-based descriptors for the targets in the DUD-E^+^ dataset.

|  | **AUC** | | | **ROCe 1%** | | |
| --- | --- | --- | --- | --- | --- | --- |
| **Target** | **MAX** | **Parallel** | **Sum** | **MAX** | **Parallel** | **Sum** |
| **aa2ar** | 0.64 | 0.64 | 0.62 | 15.6 | 16.8 | 11.8 |
| **abl1** | 0.54 | 0.47 | 0.45 | 1.1 | 1.7 | 1.7 |
| **ace** | 0.73 | 0.73 | 0.75 | 7.8 | 9.2 | 6.0 |
| **aces** | 0.75 | 0.76 | 0.76 | 27.6 | 27.4 | 25.6 |
| **ada** | 0.52 | 0.52 | 0.53 | 1.1 | 1.1 | 0.0 |
| **ada17** | 0.54 | 0.55 | 0.54 | 4.0 | 4.5 | 3.6 |
| **adrb1** | 0.64 | 0.64 | 0.62 | 19.4 | 15.0 | 10.5 |
| **adrb2** | 0.61 | 0.61 | 0.6 | 15.6 | 15.2 | 13.0 |
| **akt1** | 0.54 | 0.55 | 0.53 | 0.7 | 1.0 | 0.0 |
| **akt2** | 0.51 | 0.47 | 0.46 | 4.3 | 2.6 | 4.3 |
| **aldr** | 0.58 | 0.58 | 0.56 | 10.1 | 12.0 | 8.2 |
| **andr** | 0.63 | 0.63 | 0.63 | 6.7 | 8.2 | 4.8 |
| **aofb** | 0.68 | 0.68 | 0.67 | 17.2 | 15.6 | 15.6 |
| **bace1** | 0.75 | 0.75 | 0.74 | 28.3 | 31.5 | 27.6 |
| **braf** | 0.4 | 0.46 | 0.5 | 0.0 | 0.0 | 0.0 |
| **cah2** | 0.68 | 0.68 | 0.62 | 17.7 | 15.9 | 9.2 |
| **casp3** | 0.7 | 0.71 | 0.71 | 8.0 | 8.0 | 4.5 |
| **cdk2** | 0.69 | 0.69 | 0.66 | 4.6 | 7.4 | 4.9 |
| **comt** | 0.63 | 0.63 | 0.63 | 7.3 | 7.3 | 4.9 |
| **csf1r** | 0.72 | 0.73 | 0.7 | 28.9 | 28.9 | 22.3 |
| **def** | 0.39 | 0.39 | 0.39 | 1.0 | 1.0 | 0.0 |
| **dhi1** | 0.72 | 0.72 | 0.73 | 15.8 | 15.2 | 12.4 |
| **dpp4** | 0.61 | 0.61 | 0.61 | 4.1 | 3.9 | 3.4 |
| **dyr** | 0.6 | 0.65 | 0.63 | 6.5 | 8.2 | 3.9 |
| **egfr** | 0.64 | 0.64 | 0.63 | 12.9 | 13.3 | 14.0 |
| **esr1** | 0.68 | 0.68 | 0.67 | 44.4 | 44.4 | 42.6 |
| **esr2** | 0.7 | 0.71 | 0.69 | 40.1 | 42.8 | 38.2 |
| **fa10** | 0.53 | 0.55 | 0.56 | 6.3 | 6.5 | 3.0 |
| **fa7** | 0.8 | 0.8 | 0.78 | 21.1 | 20.2 | 13.2 |
| **fabp4** | 0.63 | 0.63 | 0.63 | 0.0 | 0.0 | 0.0 |
| **fak1** | 0.58 | 0.58 | 0.58 | 5.0 | 5.0 | 5.0 |
| **fgfr1** | 0.7 | 0.7 | 0.7 | 22.3 | 21.6 | 20.1 |
| **fkb1a** | 0.82 | 0.82 | 0.81 | 45.1 | 44.1 | 41.4 |
| **fnta** | 0.63 | 0.65 | 0.67 | 5.2 | 5.7 | 6.3 |
| **gcr** | 0.55 | 0.54 | 0.54 | 3.9 | 4.3 | 4.3 |
| **glcm** | 0.61 | 0.62 | 0.6 | 16.7 | 16.7 | 14.8 |
| **gria2** | 0.52 | 0.52 | 0.48 | 1.9 | 1.3 | 0.6 |
| **grik1** | 0.64 | 0.64 | 0.63 | 5.9 | 7.9 | 5.0 |
| **hdac2** | 0.59 | 0.59 | 0.6 | 2.2 | 2.2 | 3.2 |
| **hdac8** | 0.44 | 0.45 | 0.47 | 0.0 | 0.0 | 0.0 |
| **hivint** | 0.41 | 0.41 | 0.41 | 0.0 | 0.0 | 1.0 |
| **hivpr** | 0.81 | 0.81 | 0.82 | 30.6 | 32.1 | 32.3 |
| **hivrt** | 0.51 | 0.55 | 0.46 | 0.0 | 0.3 | 0.3 |
| **hmdh** | 0.89 | 0.89 | 0.89 | 61.8 | 61.2 | 64.7 |
| **hs90a** | 0.73 | 0.74 | 0.74 | 31.8 | 33.0 | 31.8 |
| **hxk4** | 0.81 | 0.8 | 0.78 | 4.4 | 6.5 | 2.2 |
| **inha** | 0.79 | 0.8 | 0.81 | 0.0 | 2.3 | 9.3 |
| **jak2** | 0.65 | 0.65 | 0.64 | 5.6 | 5.6 | 4.7 |
| **kif11** | 0.7 | 0.7 | 0.69 | 8.6 | 7.8 | 7.8 |
| **lck** | 0.28 | 0.26 | 0.25 | 1.2 | 1.0 | 0.7 |
| **lkha4** | 0.36 | 0.37 | 0.35 | 0.0 | 0.0 | 0.0 |
| **mapk2** | 0.8 | 0.79 | 0.77 | 19.8 | 23.8 | 10.9 |
| **mcr** | 0.51 | 0.54 | 0.54 | 2.1 | 2.1 | 1.1 |
| **met** | 0.76 | 0.76 | 0.76 | 20.5 | 19.9 | 19.9 |
| **mk01** | 0.84 | 0.84 | 0.79 | 34.2 | 36.7 | 31.7 |
| **mk10** | 0.55 | 0.55 | 0.54 | 5.8 | 4.8 | 3.9 |
| **mk14** | 0.63 | 0.65 | 0.64 | 11.4 | 11.8 | 11.1 |
| **mmp13** | 0.72 | 0.71 | 0.72 | 17.1 | 18.9 | 18.0 |
| **mp2k1** | 0.56 | 0.56 | 0.55 | 3.3 | 3.3 | 4.1 |
| **nos1** | 0.44 | 0.44 | 0.44 | 0.0 | 0.0 | 0.0 |
| **nram** | 0.86 | 0.86 | 0.87 | 17.4 | 20.4 | 17.4 |
| **pa2ga** | 0.73 | 0.7 | 0.67 | 3.0 | 3.0 | 5.1 |
| **parp1** | 0.74 | 0.74 | 0.73 | 24.6 | 24.2 | 21.1 |
| **pde5a** | 0.66 | 0.68 | 0.64 | 14.3 | 14.6 | 12.6 |
| **pgh1** | 0.5 | 0.51 | 0.49 | 1.0 | 1.0 | 0.5 |
| **pgh2** | 0.48 | 0.51 | 0.48 | 0.9 | 0.9 | 1.8 |
| **plk1** | 0.67 | 0.69 | 0.64 | 10.3 | 8.4 | 4.7 |
| **pnph** | 0.7 | 0.7 | 0.69 | 24.3 | 24.3 | 25.2 |
| **ppara** | 0.88 | 0.88 | 0.87 | 31.6 | 30.8 | 32.2 |
| **ppard** | 0.89 | 0.89 | 0.88 | 24.6 | 22.5 | 23.3 |
| **pparg** | 0.38 | 0.38 | 0.36 | 1.0 | 1.0 | 0.6 |
| **prgr** | 0.77 | 0.78 | 0.76 | 24.6 | 25.6 | 22.5 |
| **ptn1** | 0.58 | 0.58 | 0.57 | 5.4 | 6.9 | 6.9 |
| **pur2** | 0.95 | 0.95 | 0.92 | 78.0 | 80.0 | 78.0 |
| **pygm** | 0.43 | 0.44 | 0.44 | 0.0 | 0.0 | 0.0 |
| **pyrd** | 0.61 | 0.6 | 0.62 | 7.2 | 7.2 | 11.7 |
| **reni** | 0.61 | 0.6 | 0.62 | 13.5 | 14.4 | 11.5 |
| **rock1** | 0.53 | 0.53 | 0.52 | 1.0 | 1.0 | 1.0 |
| **rxra** | 0.92 | 0.92 | 0.91 | 62.6 | 63.4 | 64.9 |
| **sahh** | 0.88 | 0.88 | 0.87 | 66.7 | 66.7 | 61.9 |
| **src** | 0.74 | 0.74 | 0.73 | 19.3 | 19.9 | 22.9 |
| **tgfr1** | 0.47 | 0.47 | 0.48 | 0.8 | 0.8 | 1.5 |
| **thb** | 0.86 | 0.88 | 0.83 | 43.7 | 41.8 | 27.2 |
| **thrb** | 0.44 | 0.45 | 0.45 | 0.0 | 0.4 | 0.0 |
| **try1** | 0.52 | 0.52 | 0.5 | 2.0 | 2.2 | 2.0 |
| **tryb1** | 0.46 | 0.49 | 0.38 | 2.7 | 2.7 | 0.0 |
| **tysy** | 0.76 | 0.77 | 0.72 | 47.7 | 48.6 | 20.2 |
| **urok** | 0.47 | 0.47 | 0.44 | 3.7 | 4.3 | 4.3 |
| **wee1** | 0.47 | 0.47 | 0.48 | 5.9 | 3.9 | 4.9 |
| **xiap** | 0.88 | 0.87 | 0.85 | 19.0 | 12.0 | 24.0 |

**Table S9.** ROCe 2% and ROCe 5% values of the *Q_ENS_* query using MAX score. parallel selection and rank sum fusion algorithms and the hydrophobicity (Hyphar)-based descriptors for the targets in the DUD-E^+^ dataset.

|  | **ROCe 2%** | | | **ROCe 5%** | | |
| --- | --- | --- | --- | --- | --- | --- |
| **Target** | **MAX** | **Parallel** | **Sum** | **MAX** | **Parallel** | **Sum** |
| **aa2ar** | 10.0 | 10.5 | 7.0 | 5.0 | 5.1 | 4.1 |
| **abl1** | 1.4 | 0.8 | 1.1 | 1.0 | 1.1 | 0.7 |
| **ace** | 6.7 | 7.3 | 6.2 | 5.1 | 5.3 | 6.0 |
| **aces** | 14.9 | 15.1 | 13.9 | 7.4 | 7.7 | 7.2 |
| **ada** | 0.5 | 0.5 | 0.5 | 0.4 | 0.4 | 0.2 |
| **ada17** | 3.0 | 2.8 | 2.7 | 2.0 | 2.0 | 2.0 |
| **adrb1** | 11.7 | 10.5 | 7.9 | 6.7 | 6.2 | 4.8 |
| **adrb2** | 9.5 | 9.1 | 8.0 | 5.0 | 5.0 | 4.5 |
| **akt1** | 1.0 | 1.2 | 0.2 | 1.0 | 1.1 | 0.6 |
| **akt2** | 2.6 | 2.6 | 3.0 | 1.7 | 1.9 | 1.9 |
| **aldr** | 6.9 | 7.9 | 6.3 | 3.8 | 3.7 | 3.4 |
| **andr** | 5.0 | 5.4 | 3.2 | 2.8 | 3.1 | 3.1 |
| **aofb** | 9.8 | 9.8 | 10.3 | 5.6 | 5.3 | 5.1 |
| **bace1** | 18.2 | 18.9 | 18.0 | 9.4 | 9.3 | 9.3 |
| **braf** | 0.0 | 0.3 | 0.0 | 0.3 | 0.3 | 0.0 |
| **cah2** | 10.6 | 10.4 | 6.2 | 6.0 | 5.7 | 4.3 |
| **casp3** | 7.3 | 7.0 | 6.3 | 4.8 | 5.1 | 4.7 |
| **cdk2** | 4.1 | 5.5 | 4.1 | 3.3 | 4.1 | 3.4 |
| **comt** | 3.7 | 3.7 | 4.9 | 3.9 | 3.4 | 4.4 |
| **csf1r** | 16.0 | 15.7 | 13.0 | 8.0 | 8.2 | 7.0 |
| **def** | 1.5 | 0.5 | 0.0 | 1.0 | 0.6 | 0.2 |
| **dhi1** | 9.9 | 9.1 | 8.8 | 6.6 | 6.4 | 4.9 |
| **dpp4** | 3.0 | 2.6 | 2.4 | 2.4 | 2.3 | 1.9 |
| **dyr** | 5.6 | 5.6 | 3.0 | 3.3 | 3.7 | 3.1 |
| **egfr** | 9.0 | 8.5 | 8.7 | 4.7 | 4.7 | 4.6 |
| **esr1** | 23.5 | 23.2 | 22.7 | 9.9 | 9.8 | 9.8 |
| **esr2** | 21.7 | 22.6 | 20.6 | 9.3 | 9.7 | 9.1 |
| **fa10** | 4.2 | 4.3 | 2.3 | 2.2 | 2.5 | 1.8 |
| **fa7** | 12.7 | 12.7 | 8.3 | 6.8 | 6.8 | 5.3 |
| **fabp4** | 0.0 | 0.0 | 0.0 | 0.9 | 1.3 | 0.9 |
| **fak1** | 3.5 | 3.0 | 3.0 | 2.2 | 2.4 | 2.0 |
| **fgfr1** | 13.3 | 12.6 | 13.3 | 6.9 | 6.9 | 6.6 |
| **fkb1a** | 23.9 | 23.4 | 22.5 | 11.2 | 11.4 | 11.2 |
| **fnta** | 3.5 | 3.7 | 4.3 | 2.3 | 2.3 | 3.1 |
| **gcr** | 2.1 | 2.1 | 2.3 | 1.4 | 1.4 | 1.0 |
| **glcm** | 10.2 | 11.1 | 9.3 | 5.2 | 5.2 | 5.2 |
| **gria2** | 1.0 | 1.3 | 1.0 | 0.8 | 0.9 | 0.6 |
| **grik1** | 5.5 | 5.0 | 5.5 | 4.6 | 5.2 | 3.0 |
| **hdac2** | 1.4 | 1.6 | 2.2 | 1.5 | 1.5 | 2.1 |
| **hdac8** | 0.0 | 0.6 | 0.0 | 0.6 | 0.6 | 0.5 |
| **hivint** | 0.5 | 0.5 | 0.5 | 0.6 | 0.4 | 0.8 |
| **hivpr** | 20.0 | 21.0 | 19.8 | 10.4 | 10.5 | 10.3 |
| **hivrt** | 0.4 | 0.9 | 0.4 | 0.7 | 0.7 | 0.7 |
| **hmdh** | 35.3 | 34.7 | 35.0 | 14.9 | 14.9 | 14.6 |
| **hs90a** | 16.5 | 17.1 | 16.5 | 8.0 | 8.2 | 7.3 |
| **hxk4** | 5.4 | 7.1 | 2.7 | 5.0 | 4.1 | 2.0 |
| **inha** | 8.1 | 4.7 | 8.1 | 5.1 | 5.1 | 6.1 |
| **jak2** | 4.7 | 5.6 | 5.1 | 3.4 | 3.7 | 3.2 |
| **kif11** | 5.6 | 5.6 | 4.7 | 3.8 | 4.0 | 2.6 |
| **lck** | 1.0 | 0.8 | 0.4 | 0.7 | 0.6 | 0.3 |
| **lkha4** | 0.0 | 0.0 | 0.6 | 0.4 | 0.4 | 0.5 |
| **mapk2** | 12.4 | 13.9 | 6.4 | 6.5 | 7.7 | 5.2 |
| **mcr** | 1.6 | 1.6 | 1.1 | 0.9 | 0.9 | 0.9 |
| **met** | 13.6 | 13.3 | 11.5 | 7.8 | 8.3 | 7.7 |
| **mk01** | 19.6 | 20.9 | 17.1 | 9.4 | 9.4 | 7.6 |
| **mk10** | 2.9 | 2.9 | 2.4 | 1.9 | 2.1 | 1.4 |
| **mk14** | 7.4 | 7.8 | 8.0 | 5.0 | 4.9 | 5.5 |
| **mmp13** | 9.8 | 10.8 | 10.4 | 5.4 | 5.7 | 5.2 |
| **mp2k1** | 2.5 | 2.5 | 2.5 | 1.3 | 1.5 | 1.8 |
| **nos1** | 0.0 | 0.0 | 0.0 | 0.4 | 0.6 | 0.6 |
| **nram** | 13.3 | 13.3 | 11.7 | 8.2 | 7.8 | 7.8 |
| **pa2ga** | 4.0 | 3.5 | 6.6 | 3.6 | 4.2 | 4.9 |
| **parp1** | 14.0 | 13.9 | 12.6 | 7.2 | 7.3 | 6.6 |
| **pde5a** | 8.2 | 8.2 | 7.7 | 3.9 | 4.1 | 3.7 |
| **pgh1** | 0.5 | 0.5 | 0.8 | 0.5 | 0.6 | 0.5 |
| **pgh2** | 1.5 | 1.4 | 1.5 | 1.2 | 1.2 | 1.3 |
| **plk1** | 6.1 | 6.1 | 3.7 | 3.6 | 3.7 | 2.8 |
| **pnph** | 13.6 | 13.1 | 14.6 | 6.4 | 6.6 | 6.0 |
| **ppara** | 20.8 | 20.2 | 19.0 | 10.5 | 10.3 | 10.4 |
| **ppard** | 18.1 | 17.5 | 19.0 | 12.1 | 11.7 | 12.2 |
| **pparg** | 0.6 | 0.6 | 0.5 | 0.7 | 0.7 | 0.5 |
| **prgr** | 14.5 | 15.0 | 13.7 | 7.6 | 7.6 | 7.0 |
| **ptn1** | 3.5 | 5.4 | 4.6 | 2.3 | 2.5 | 3.4 |
| **pur2** | 41.0 | 41.0 | 40.0 | 17.6 | 17.6 | 16.4 |
| **pygm** | 0.0 | 0.0 | 0.0 | 0.0 | 0.0 | 0.0 |
| **pyrd** | 5.0 | 5.4 | 9.0 | 3.8 | 3.6 | 4.5 |
| **reni** | 8.2 | 7.7 | 6.7 | 4.4 | 3.9 | 3.7 |
| **rock1** | 2.0 | 2.5 | 1.0 | 1.6 | 1.8 | 0.4 |
| **rxra** | 36.3 | 35.1 | 34.4 | 15.7 | 15.4 | 15.0 |
| **sahh** | 35.7 | 35.7 | 34.1 | 15.2 | 15.2 | 14.3 |
| **src** | 15.3 | 15.0 | 14.8 | 8.4 | 8.4 | 7.6 |
| **tgfr1** | 1.1 | 1.9 | 1.9 | 1.4 | 1.2 | 1.2 |
| **thb** | 25.2 | 25.2 | 18.9 | 12.4 | 12.4 | 9.7 |
| **thrb** | 0.2 | 0.2 | 0.0 | 0.3 | 0.4 | 0.1 |
| **try1** | 2.2 | 2.2 | 1.6 | 1.6 | 1.6 | 1.3 |
| **tryb1** | 2.4 | 2.4 | 0.7 | 1.4 | 1.4 | 1.0 |
| **tysy** | 26.2 | 26.2 | 11.5 | 11.4 | 11.6 | 7.7 |
| **urok** | 2.8 | 2.8 | 2.5 | 1.6 | 1.6 | 1.5 |
| **wee1** | 3.4 | 3.9 | 3.4 | 2.0 | 2.0 | 2.4 |
| **xiap** | 19.5 | 15.0 | 14.5 | 12.6 | 10.8 | 9.4 |

**Table S10.** Statistical summary of VS metrics for *Q_XR_* and *Q_LEG_* queries obtained using the Phase shape-based descriptors for the set of targets included in the DUD-E^+^ dataset. The AUC and ROCe values (mean ± standard deviation) correspond to the average determined for the set of targets included in the DUD-E^+^ dataset (see individual values in Table S11)

|  | **AUC** | **ROCe 1%** | **ROCe 2%** | **ROCe 5%** |
| --- | --- | --- | --- | --- |
| Q_XR_ | 0.69 ± 0.13 | 17.4 ± 19.4 | 10.5 ± 10.1 | 5.7 ± 4.3 |
| Q_LEG_ | 0.69 ± 0.13 | 16.6 ± 19.3 | 10.2 ± 10.1 | 5.5 ± 4.3 |

**Table S11.** AUC. ROCe 1%. ROCe2% and ROCe 5% values determined for *Q_XR_* and *Q_LEG_* queries using the Phase shape-based descriptors or the set of targets included in the DUD-E^+^ dataset.

|  |  | **AUC** | | **ROCe 1%** | | **ROCe 2%** | | **ROCe 5%** | |
| --- | --- | --- | --- | --- | --- | --- | --- | --- | --- |
| **Target** | **Reference ID**  Target-Ligand | ***Q_XR_*** | ***Q_LEG_*** | ***Q_XR_*** | ***Q_LEG_*** | ***Q_XR_*** | ***Q_LEG_*** | ***Q_XR_*** | ***Q_LEG_*** |
| **aa2ar** | 5K2D-ZMA | 0.68 | 0.68 | 12.2 | 15.6 | 7.3 | 9.8 | 4.4 | 5.5 |
| **abl1** | 2F4J-VX6 | 0.56 | 0.51 | 3.3 | 1.7 | 3.0 | 1.7 | 2.6 | 1.0 |
| **ace** | 6F9U-D0W | 0.79 | 0.78 | 19.2 | 17.4 | 12.9 | 11.4 | 8.2 | 7.9 |
| **aces** | 2CMF-F11 | 0.74 | 0.78 | 28.0 | 33.3 | 16.9 | 19.8 | 8.8 | 10.1 |
| **ada** | 1WXY-FRK | 0.51 | 0.57 | 0.0 | 0.0 | 0.0 | 0.5 | 0.7 | 0.2 |
| **ada17** | 3EDZ-INN | 0.52 | 0.61 | 8.7 | 4.7 | 5.0 | 3.3 | 2.7 | 2.2 |
| **adrb1** | 4AMJ-CVD | 0.74 | 0.74 | 15.0 | 10.5 | 10.9 | 8.3 | 6.1 | 5.9 |
| **adrb2** | 3P0G-P0G | 0.71 | 0.67 | 9.5 | 12.6 | 6.1 | 7.8 | 3.8 | 4.2 |
| **akt1** | 3QKL-SMR | 0.64 | 0.62 | 1.0 | 0.3 | 0.9 | 0.7 | 1.0 | 1.1 |
| **akt2** | 2JDO-I5S | 0.48 | 0.54 | 1.7 | 3.4 | 2.1 | 3.0 | 1.7 | 1.9 |
| **aldr** | 4LAZ-1WW | 0.77 | 0.77 | 15.7 | 19.5 | 9.8 | 10.7 | 6.2 | 6.0 |
| **andr** | 2AXA-FHM | 0.55 | 0.56 | 1.5 | 2.2 | 1.5 | 3.0 | 1.0 | 1.9 |
| **aofb** | 2V5Z-SAG | 0.76 | 0.77 | 17.2 | 14.8 | 9.8 | 9.8 | 5.7 | 5.4 |
| **bace1** | 2QMD-CS7 | 0.74 | 0.71 | 30.0 | 25.8 | 17.1 | 16.3 | 8.7 | 7.6 |
| **braf** | 3OG7-032 | 0.75 | 0.76 | 7.9 | 2.0 | 5.3 | 2.3 | 4.0 | 2.6 |
| **cah2** | 1BN4-AL9 | 0.59 | 0.57 | 5.3 | 3.3 | 4.4 | 3.1 | 3.2 | 2.5 |
| **casp3** | 4QU9-ACE | 0.60 | 0.65 | 15.6 | 13.1 | 9.8 | 10.6 | 4.7 | 6.3 |
| **cdk2** | 1OGU-ST8 | 0.66 | 0.72 | 4.4 | 9.1 | 3.7 | 6.4 | 2.9 | 5.2 |
| **comt** | 4XUD-43H | 0.61 | 0.61 | 4.9 | 4.9 | 2.4 | 2.4 | 2.0 | 1.5 |
| **csf1r** | 3DPK-8C5 | 0.76 | 0.76 | 22.3 | 20.5 | 14.8 | 13.9 | 8.3 | 7.2 |
| **def** | 1ICJ-2PE | 0.76 | 0.77 | 20.6 | 19.6 | 10.8 | 11.8 | 7.7 | 7.3 |
| **dhi1** | 3FRJ-A491 | 0.73 | 0.73 | 18.5 | 21.8 | 13.3 | 13.8 | 7.5 | 7.1 |
| **dpp4** | 2HHA-3TP | 0.55 | 0.51 | 9.6 | 6.2 | 5.6 | 4.7 | 3.3 | 2.8 |
| **dyr** | 3FS6-DH1 | 0.63 | 0.65 | 3.9 | 3.5 | 3.7 | 3.9 | 2.6 | 3.4 |
| **egfr** | 3BEL-POX | 0.83 | 0.84 | 20.7 | 26.0 | 14.4 | 15.1 | 8.1 | 8.3 |
| **esr1** | 2R6Y-LLC1 | 0.82 | 0.82 | 47.5 | 46.5 | 24.7 | 27.0 | 11.5 | 12.0 |
| **esr2** | 2FSZ-OHT | 0.83 | 0.83 | 42.8 | 46.1 | 24.7 | 25.6 | 12.0 | 12.3 |
| **fa10** | 2XBX-RR8 | 0.77 | 0.78 | 23.1 | 21.0 | 14.3 | 12.6 | 8.0 | 7.6 |
| **fa7** | 2F9B-N1H | 0.83 | 0.84 | 14.9 | 13.2 | 9.7 | 11.0 | 7.0 | 7.0 |
| **fabp4** | 5HZ8-65Z | 0.61 | 0.59 | 0.0 | 0.0 | 0.0 | 1.1 | 1.7 | 1.3 |
| **fak1** | 4GU6-10N1 | 0.92 | 0.89 | 47.0 | 19.0 | 29.0 | 18.5 | 14.0 | 10.8 |
| **fgfr1** | 4RWJ-66T | 0.74 | 0.72 | 7.9 | 7.2 | 6.8 | 6.1 | 3.5 | 4.0 |
| **fkb1a** | 1FKH-SBX | 0.89 | 0.91 | 63.1 | 61.3 | 34.2 | 35.6 | 15.0 | 15.3 |
| **fnta** | 2IEJ-S48 | 0.74 | 0.74 | 8.5 | 14.7 | 6.3 | 10.7 | 5.0 | 6.7 |
| **fpps** | 1YV5-RIS1 | 0.60 | 0.76 | 36.5 | 38.8 | 18.8 | 19.4 | 8.0 | 8.0 |
| **gcr** | 3E7C-866 | 0.69 | 0.66 | 8.1 | 5.0 | 5.0 | 4.3 | 3.6 | 2.9 |
| **glcm** | 2V3E-NND | 0.71 | 0.72 | 13.0 | 14.8 | 7.4 | 10.2 | 4.8 | 4.8 |
| **gria2** | 3H03-UBP1 | 0.43 | 0.43 | 4.4 | 5.1 | 2.2 | 2.5 | 1.1 | 1.1 |
| **grik1** | 1VSO-AT11 | 0.53 | 0.53 | 12.9 | 10.9 | 7.4 | 7.4 | 3.6 | 3.6 |
| **hdac2** | 4LY1-20Y | 0.65 | 0.62 | 14.1 | 12.4 | 8.4 | 7.3 | 4.2 | 4.4 |
| **hdac8** | 3SFF-0DI | 0.60 | 0.55 | 2.9 | 2.4 | 2.9 | 1.5 | 2.2 | 1.3 |
| **hivint** | 5KGW-7SK | 0.63 | 0.63 | 1.0 | 2.0 | 2.0 | 1.5 | 1.6 | 1.4 |
| **hivpr** | 1EBZ-BEC | 0.81 | 0.80 | 25.0 | 24.1 | 17.3 | 16.0 | 9.7 | 9.5 |
| **hivrt** | 6ELI-T27 | 0.62 | 0.61 | 1.8 | 2.1 | 1.9 | 1.8 | 1.8 | 1.9 |
| **hmdh** | 2R4F-RIE1 | 0.82 | 0.82 | 53.5 | 40.6 | 28.8 | 24.1 | 12.5 | 11.3 |
| **hs90a** | 1UY9-PU6 | 0.78 | 0.80 | 36.4 | 34.1 | 21.0 | 18.8 | 10.2 | 10.5 |
| **hxk4** | 4IXC-1JD | 0.64 | 0.63 | 1.1 | 1.1 | 3.3 | 2.2 | 5.0 | 2.4 |
| **inha** | 5CPF-53K2 | 0.72 | 0.73 | 2.3 | 0.0 | 3.5 | 0.0 | 1.9 | 0.9 |
| **jak2** | 5WIM-35R | 0.74 | 0.71 | 6.5 | 7.5 | 4.7 | 4.7 | 3.9 | 3.6 |
| **kif11** | 4A5Y-G7X | 0.68 | 0.69 | 8.6 | 8.6 | 5.2 | 4.7 | 2.8 | 2.6 |
| **lck** | 2OF2-547 | 0.65 | 0.65 | 6.7 | 6.0 | 4.4 | 4.1 | 3.2 | 2.3 |
| **lkha4** | 3FTX-BES | 0.41 | 0.50 | 0.6 | 0.6 | 0.6 | 0.6 | 0.6 | 0.6 |
| **mapk2** | 2PZY-B18 | 0.91 | 0.90 | 38.6 | 43.6 | 22.3 | 22.8 | 11.7 | 12.5 |
| **mcr** | 5L7G-6QE | 0.67 | 0.56 | 3.2 | 1.1 | 5.3 | 1.1 | 4.3 | 2.1 |
| **met** | 5HTI-66L | 0.83 | 0.84 | 65.1 | 61.5 | 33.1 | 31.3 | 13.5 | 13.1 |
| **mk01** | 3I5Z-Z48 | 0.83 | 0.84 | 49.4 | 58.2 | 28.5 | 29.1 | 11.9 | 12.4 |
| **mk10** | 4Y5H-519 | 0.44 | 0.47 | 5.8 | 4.8 | 3.4 | 3.9 | 1.7 | 2.1 |
| **mk14** | 3D83-GK6 | 0.85 | 0.83 | 27.0 | 25.4 | 17.0 | 15.1 | 8.7 | 8.2 |
| **mmp13** | 3ELM-24F | 0.68 | 0.67 | 17.5 | 19.4 | 11.4 | 11.9 | 6.1 | 6.3 |
| **mp2k1** | 3DV3-MEK1 | 0.77 | 0.76 | 10.7 | 9.1 | 5.8 | 6.2 | 3.5 | 3.5 |
| **nos1** | 1QWC-H4B | 0.46 | 0.46 | 3.0 | 3.0 | 1.5 | 2.0 | 1.0 | 1.0 |
| **nram** | 1B9V-RA2 | 0.46 | 0.46 | 1.0 | 1.0 | 1.0 | 1.0 | 0.4 | 0.4 |
| **pa2ga** | 1AYP-INB | 0.53 | 0.55 | 1.0 | 0.0 | 0.5 | 3.0 | 0.6 | 3.2 |
| **parp1** | 4R6E-3JD | 0.75 | 0.76 | 19.7 | 19.3 | 11.2 | 11.6 | 6.1 | 6.3 |
| **pde5a** | 3TGE-TGE | 0.65 | 0.66 | 19.4 | 18.1 | 11.7 | 11.1 | 6.8 | 6.3 |
| **pgh1** | 2OYE-IM8 | 0.72 | 0.71 | 17.4 | 19.5 | 11.0 | 11.5 | 5.9 | 5.3 |
| **pgh2** | 5W58-FF81 | 0.71 | 0.71 | 5.5 | 4.4 | 5.8 | 4.5 | 5.0 | 4.0 |
| **plk1** | 4A4L-939 | 0.70 | 0.78 | 12.2 | 15.0 | 6.1 | 8.9 | 4.3 | 5.2 |
| **pnph** | 4EAR-IM52 | 0.80 | 0.79 | 31.1 | 31.1 | 17.0 | 18.5 | 7.8 | 7.8 |
| **ppara** | 1I7G-AZ2 | 0.65 | 0.49 | 6.7 | 3.5 | 5.1 | 2.3 | 3.7 | 2.0 |
| **ppard** | 5U3Z-7UA | 0.55 | 0.67 | 0.4 | 4.2 | 0.4 | 4.0 | 0.5 | 2.8 |
| **pparg** | 5YCP-BRL | 0.65 | 0.48 | 6.6 | 2.1 | 4.7 | 1.5 | 3.3 | 1.6 |
| **prgr** | 2OVM-AS0 | 0.76 | 0.77 | 14.7 | 15.4 | 8.9 | 9.0 | 5.2 | 5.3 |
| **ptn1** | 1NL9-989 | 0.52 | 0.52 | 4.6 | 3.9 | 2.7 | 1.9 | 1.2 | 1.7 |
| **pur2** | 4EW3-DXZ | 0.99 | 1.00 | 86.0 | 90.0 | 44.0 | 49.0 | 19.2 | 19.6 |
| **pygm** | 1WV0-BN41 | 0.60 | 0.63 | 0.0 | 0.0 | 0.0 | 0.0 | 1.6 | 0.3 |
| **pyrd** | 3KVJ-1X5 | 0.49 | 0.47 | 0.9 | 0.9 | 0.5 | 0.5 | 0.9 | 0.5 |
| **reni** | 2V16-C472 | 0.59 | 0.58 | 3.9 | 7.7 | 3.9 | 4.3 | 3.7 | 4.0 |
| **rock1** | 5KKS-6U1 | 0.76 | 0.74 | 10.0 | 6.0 | 11.0 | 5.5 | 5.6 | 4.6 |
| **rxra** | 4RMD-3SW | 0.93 | 0.93 | 69.5 | 71.8 | 38.2 | 38.9 | 16.0 | 16.2 |
| **sahh** | 3NJ4-AFX3 | 0.97 | 0.97 | 84.1 | 85.7 | 42.1 | 44.4 | 17.8 | 18.4 |
| **src** | 2OIQ-STI | 0.74 | 0.73 | 9.0 | 7.8 | 6.4 | 5.2 | 4.9 | 4.1 |
| **tgfr1** | 2X7O-ZOP | 0.92 | 0.92 | 7.5 | 6.0 | 8.3 | 9.4 | 9.0 | 9.3 |
| **thb** | 1Q4X-G24 | 0.67 | 0.71 | 10.7 | 17.5 | 8.3 | 12.6 | 4.5 | 7.0 |
| **thrb** | 2C8Y-C3M | 0.58 | 0.48 | 0.9 | 0.0 | 1.0 | 0.0 | 1.3 | 0.4 |
| **try1** | 1O3D-780 | 0.62 | 0.61 | 5.6 | 8.2 | 5.5 | 5.5 | 3.2 | 3.7 |
| **tryb1** | 5F03-5TA | 0.51 | 0.45 | 3.4 | 2.7 | 2.4 | 1.7 | 1.6 | 1.0 |
| **tysy** | 1TRG-CB3 | 0.88 | 0.86 | 50.5 | 43.1 | 29.4 | 23.4 | 13.4 | 11.2 |
| **urok** | 4FU7-1UP | 0.61 | 0.61 | 4.9 | 4.9 | 3.4 | 4.0 | 2.4 | 2.4 |
| **vgfr2** | 2XIR-00J | 0.86 | 0.83 | 31.1 | 23.2 | 20.3 | 15.8 | 10.5 | 9.0 |
| **wee1** | 5VD4-99J | 0.79 | 0.79 | 13.7 | 0.0 | 9.3 | 2.5 | 5.7 | 2.2 |
| **xiap** | 4HY0-1AQ | 0.94 | 0.92 | 59.0 | 53.0 | 31.0 | 28.0 | 15.6 | 14.4 |

**Table S12.** Statistical summary of VS metrics for the *Q_XR_* and *Q_LEG_* queries. The AUC and ROCe values (mean ± standard deviation) correspond to the average determined using the hydrophobic (Hyphar)-based descriptors for the set of targets included in the DUD-E^+^-Diverse dataset (see individual values in Table S13)

|  | **AUC** | **ROCe 1%** | **ROCe 2%** | **ROCe 5%** |
| --- | --- | --- | --- | --- |
| Q_XR_ | 0.47 ± 0.14 | 4.6 ± 4.9 | 2.7 ± 4 | 1.7 ± 2 |
| Q_LEG_ | 0.51 ± 0.15 | 2.0 ± 3.1 | 1.6 ± 3.2 | 1.1 ± 1.7 |

**Table S13.** AUC and ROCe 1. 2 and 5% values determined for *Q_XR_* and *Q_LEG_* queries using the hydrophobic (Hyphar)-based descriptors for the set of targets included in the DUD-E^+^-Diverse dataset.

|  |  | **AUC** | | **ROCe 1%** | |
| --- | --- | --- | --- | --- | --- |
| **Target** | **Reference ID**  Target-Ligand | ***Q_XR_*** | ***Q_LEG_*** | ***Q_XR_*** | ***Q_LEG_*** |
| **aces** | 2CMF-F11 | 0.63 | 0.70 | 7.4 | 6.7 |
| **ada17** | 3EDZ-INN | 0.62 | 0.45 | 1.6 | 1.3 |
| **akt2** | 2JDO-I5S | 0.31 | 0.41 | 2.4 | 0 |
| **andr** | 2AXA-FHM | 0.56 | 0.53 | 0 | 0 |
| **casp3** | 4QU9-ACE | 0.48 | 0.46 | 0 | 0 |
| **gcr** | 3E7C-866 | 0.37 | 0.42 | 0 | 0 |
| **hivrt** | 6ELI-T27 | 0.69 | 0.42 | 6.2 | 0 |
| **hs90a** | 1UY9-PU6 | 0.55 | 0.50 | 0 | 0 |
| **mp2k1** | 3DV3-MEK1 | 0.43 | 0.47 | 1.2 | 1.2 |
| **nos1** | 1QWC-H4B | 0.47 | 0.44 | 0 | 0 |
| **pa2ga** | 1AYP-INB | 0.77 | 0.50 | 13.0 | 1.6 |
| **pgh1** | 2OYE-IM8 | 0.42 | 0.42 | 0 | 1.0 |
| **pgh2** | 5W58-FF81 | 0.32 | 0.35 | 0 | 0 |
| **prgr** | 2OVM-AS0 | 0.73 | 0.74 | 15.0 | 18.0 |
| **tgfr1** | 2X7O-ZOP | 0.31 | 0.31 | 0 | 0 |

|  |  | **ROCe 2%** | | **ROCe 5%** | |
| --- | --- | --- | --- | --- | --- |
| **Target** | **Reference ID**  Target-Ligand | ***Q_XR_*** | ***Q_LEG_*** | ***Q_XR_*** | ***Q_LEG_*** |
| **aces** | 2CMF-F11 | 5.2 | 6.3 | 3.9 | 4.4 |
| **ada17** | 3EDZ-INN | 1.5 | 1.2 | 1.3 | 0.9 |
| **akt2** | 2JDO-I5S | 1.2 | 1.2 | 1.0 | 0.5 |
| **andr** | 2AXA-FHM | 1.6 | 0.4 | 1.1 | 0.5 |
| **casp3** | 4QU9-ACE | 0 | 0.6 | 0 | 1.0 |
| **gcr** | 3E7C-866 | 0.4 | 0 | 0.3 | 0 |
| **hivrt** | 6ELI-T27 | 3.6 | 0.5 | 3.3 | 0.3 |
| **hs90a** | 1UY9-PU6 | 0 | 0 | 0.6 | 0.6 |
| **mp2k1** | 3DV3-MEK1 | 1.8 | 0.6 | 0.7 | 0.2 |
| **nos1** | 1QWC-H4B | 0 | 0 | 0.5 | 0.3 |
| **pa2ga** | 1AYP-INB | 13.7 | 0.8 | 7.0 | 0.7 |
| **pgh1** | 2OYE-IM8 | 0.5 | 0.5 | 0.8 | 0.4 |
| **pgh2** | 5W58-FF81 | 0 | 0 | 0 | 0 |
| **prgr** | 2OVM-AS0 | 10.3 | 12.2 | 5.0 | 6.2 |
| **tgfr1** | 2X7O-ZOP | 0 | 0 | 0 | 0 |
